# Publisher Correction: A lamprey neural cell type atlas illuminates the origins of the vertebrate brain

**DOI:** 10.1038/s41559-023-02227-1

**Published:** 2023-09-25

**Authors:** Francesco Lamanna, Francisca Hervas-Sotomayor, A. Phillip Oel, David Jandzik, Daniel Sobrido-Cameán, Gabriel N. Santos-Durán, Megan L. Martik, Jan Stundl, Stephen A. Green, Thoomke Brüning, Katharina Mößinger, Julia Schmidt, Celine Schneider, Mari Sepp, Florent Murat, Jeramiah J. Smith, Marianne E. Bronner, María Celina Rodicio, Antón Barreiro-Iglesias, Daniel M. Medeiros, Detlev Arendt, Henrik Kaessmann

**Affiliations:** 1 Center for Molecular Biology of Heidelberg University (ZMBH), DKFZ-ZMBH Alliance, Heidelberg, Germany; 2 Developmental Biology Unit, European Molecular Biology Laboratory, Heidelberg, Germany; 3 Department of Ecology and Evolutionary Biology, University of Colorado Boulder, Boulder, CO USA; 4 Department of Zoology, Comenius University, Bratislava, Slovakia; 5 Department of Functional Biology, CIBUS, Faculty of Biology, Universidade de Santiago de Compostela, Santiago de Compostela, Spain; 6 Division of Biology and Biological Engineering, California Institute of Technology, Pasadena, CA USA; 7 Department of Biology, University of Kentucky, Lexington, KY USA; 8 Present Address: Department of Molecular and Cell Biology, University of California Berkeley, Berkeley, CA USA; 9 Present Address: INRAE, LPGP, Rennes, France

**Keywords:** Molecular evolution, Evolutionary genetics

Correction to: *Nature Ecology & Evolution* 10.1038/s41559-023-02170-1, published online 14 September 2023.

In the version of the article initially published, the online Peer Review File mistakenly contained comments pertaining to another article. The correct Peer Review File has now been uploaded.

