# Supplementary material for: A lamprey neural cell type atlas illuminates the origins of the vertebrate brain
Source: Nat Ecol Evol. 2023 Sep 14;7(10):1714–28. doi: 10.1038/s41559-023-02170-1 (PMC10555824; doi:10.1038/s41559-023-02170-1)
Supplement: Supplementary file 1 — Supplementary Figs. 1 and 2. [file 41559_2023_2170_MOESM1_ESM.pdf]

nature ecology & evolution

Article https://doi.org/10.1038/s41559-023-02170-1

A lamprey neural cell type atlas illuminates the origins of the vertebrate brain

In the format provided by the authors and unedited

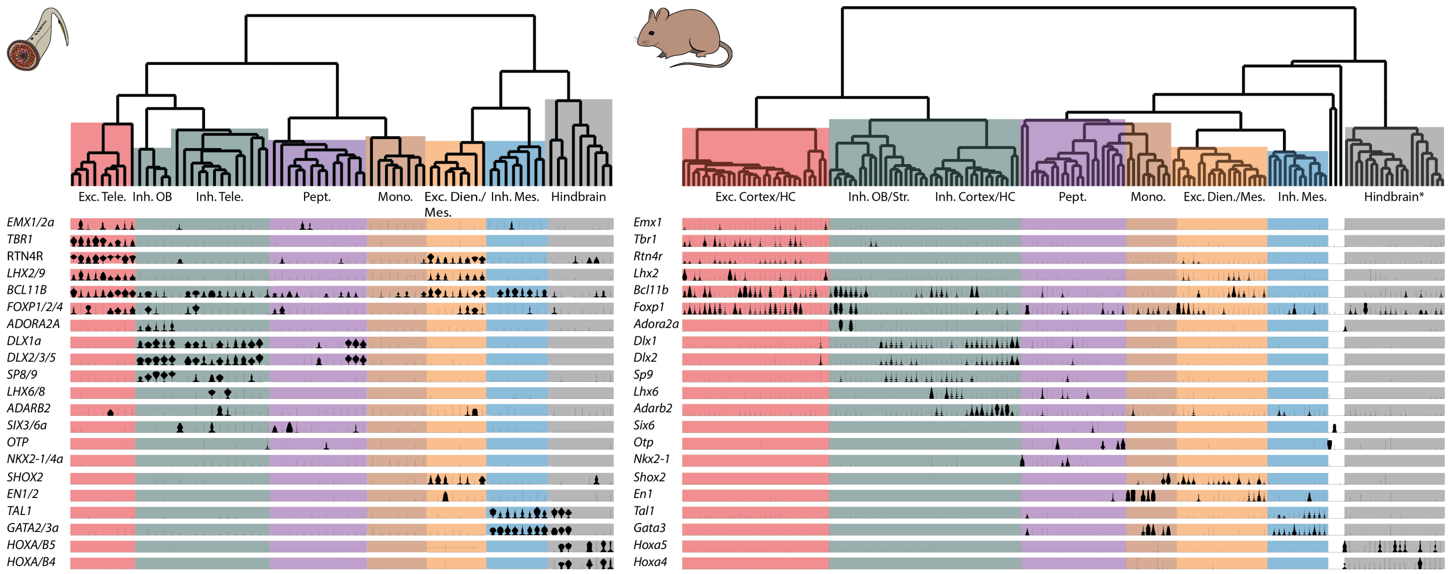

Supplementary Fig. 1 | Conservation of neuronal markers between lamprey and mouse. Upper panel: lamprey and mouse dendrograms of selected homologous neuronal families obtained based on correlations of expression levels of TF genes only. *Cerebellum excluded. Lower panel: violin plots showing the expression of selected TF genes for each cell type. Color code as in Fig. 1c.

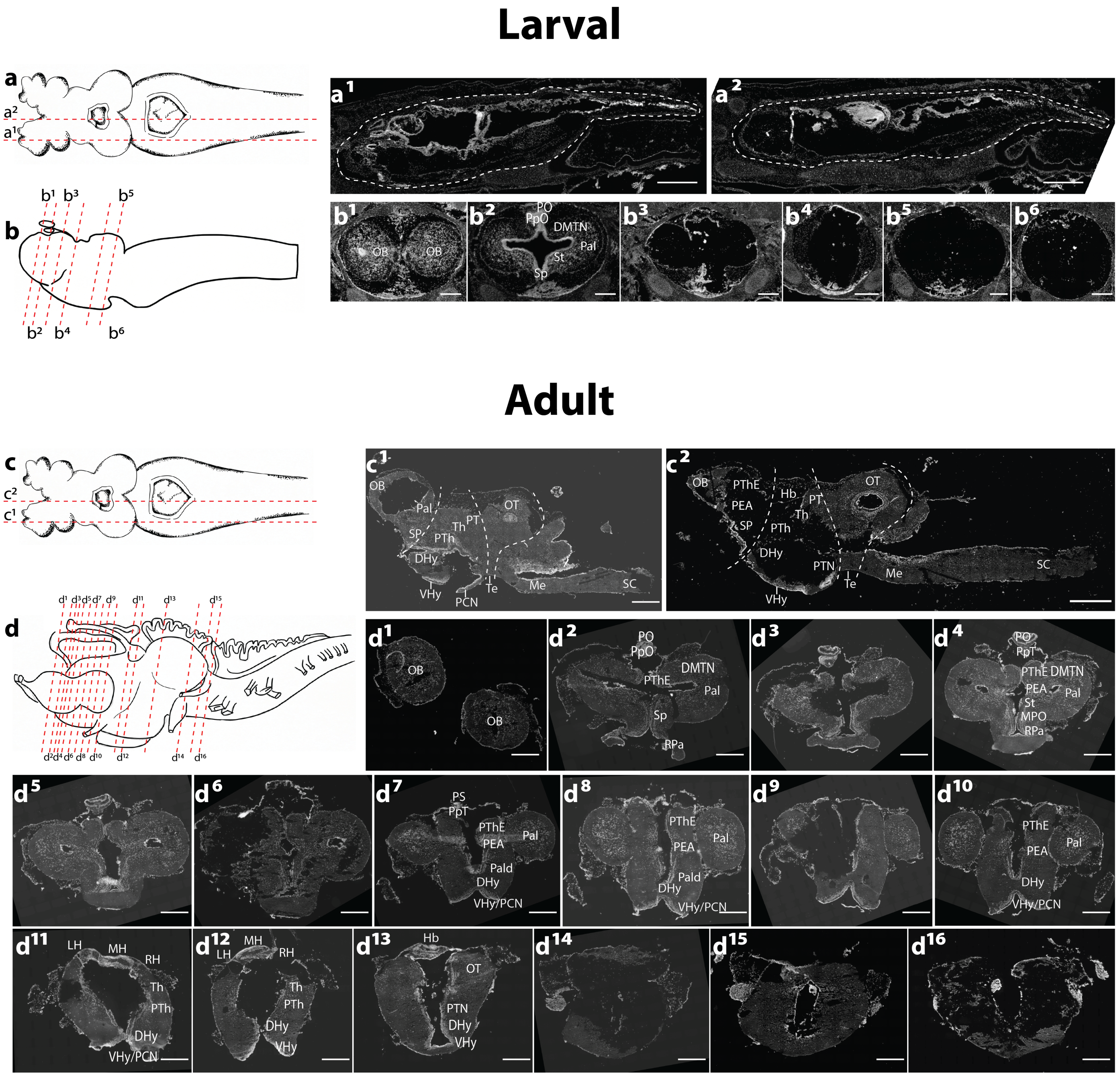

Supplementary Fig. 2 | ISS dissection schemes. a-d, ISS dissection schemes (DAPI) of sagittal (a, c) and coronal (b, d) sections of lamprey larval and adult brains. Dashed lines on adult sagittal sections separate the main brain regions. DHy, dorsal hypothalamus; DMTN, dorsomedial telencephalic nucleus; Hb, habenula; LH, left habenula; Me, medulla; MH, medial habenula; MPO, medial preoptic nucleus; OB, olfactory bulb; OT, optic tectum; Pal, pallium; Pald, pallidum; PEA, pallial extended amygdala; PCN, postoptic commissure nucle-us; PO, pineal organ; PpO, parapineal organ; PpT, parapineal tract; PS, pineal stalk; PT, pre-tectum; PTh, pre-thalamus; PThE, pre-thalamic eminence; PTN, posterior tubercle nucleus; RH, right habenula; RPa, rostral paraventricular area; SC, spinal cord; Sp, septum; St, striatum; Te, tegmentum; Th, thalamus; VHy, ventral hypothalamus. Scale bars, 500um.
