# Supplementary material for: A lamprey neural cell type atlas illuminates the origins of the vertebrate brain
Source: Nat Ecol Evol. 2023 Sep 14;7(10):1714–28. doi: 10.1038/s41559-023-02170-1 (PMC10555824; doi:10.1038/s41559-023-02170-1)
Supplement: Supplementary file 2 — Reporting Summary [file 41559_2023_2170_MOESM2_ESM.pdf]

Corresponding author(s): Francesco Lamanna, Francisca Hervas-Sotomayor, Henrik Kaessmann

Last updated by author(s): July 5, 2023

Reporting Summary

Nature Portfolio wishes to improve the reproducibility of the work that we publish. This form provides structure for consistency and transparency in reporting. For further information on Nature Portfolio policies, see our Editorial Policies and the Editorial Policy Checklist.

Statistics

For all statistical analyses, confirm that the following items are present in the figure legend, table legend, main text, or Methods section.

n/a	Confirmed	
☐	☒	The exact sample size (*n*) for each experimental group/condition, given as a discrete number and unit of measurement
☐	☒	A statement on whether measurements were taken from distinct samples or whether the same sample was measured repeatedly
☐	☒	The statistical test(s) used AND whether they are one- or two-sided<br>*Only common tests should be described solely by name; describe more complex techniques in the Methods section.*
☒	☐	A description of all covariates tested
☐	☒	A description of any assumptions or corrections, such as tests of normality and adjustment for multiple comparisons
☐	☒	A full description of the statistical parameters including central tendency (e.g. means) or other basic estimates (e.g. regression coefficient) AND variation (e.g. standard deviation) or associated estimates of uncertainty (e.g. confidence intervals)
☒	☐	For null hypothesis testing, the test statistic (e.g. *F*, *t*, *r*) with confidence intervals, effect sizes, degrees of freedom and *P* value noted<br>*Give P values as exact values whenever suitable.*
☒	☐	For Bayesian analysis, information on the choice of priors and Markov chain Monte Carlo settings
☒	☐	For hierarchical and complex designs, identification of the appropriate level for tests and full reporting of outcomes
☐	☒	Estimates of effect sizes (e.g. Cohen's *d*, Pearson's *r*), indicating how they were calculated

Our web collection on statistics for biologists contains articles on many of the points above.

Software and code

Policy information about availability of computer code

Data collection	No software was used.
Data analysis	Open source software including CellRanger v3, GSNAP (version: 2018-03-01), StringTie (v1.3.4d), GffRead (v0.9.9), TransDecoder (v5.3.0), BlastP (v2.5.0+), HMMER (v3.2), BUSCO (v3), OrthoFinder (v2.3.11), MAFFT (v7.455), IQ-TREE (v1.6.12), Seurat (v3.1.5), LIGER (v0.5.0), SAMap (v0.2.3), pvclust (v2.2.0), Fiji (v2.0)

For manuscripts utilizing custom algorithms or software that are central to the research but not yet described in published literature, software must be made available to editors and reviewers. We strongly encourage code deposition in a community repository (e.g. GitHub). See the Nature Portfolio guidelines for submitting code & software for further information.

Data

Policy information about availability of data

All manuscripts must include a data availability statement. This statement should provide the following information, where applicable:

- Accession codes, unique identifiers, or web links for publicly available datasets
- A description of any restrictions on data availability
- For clinical datasets or third party data, please ensure that the statement adheres to our policy

Raw and processed bulk and single-cell RNA-seq data have been deposited to ArrayExpress with the accession numbers E-MTAB-11085 (bulk) and E-MTAB-11087 (single cell) (https://www.ebi.ac.uk/arrayexpress/).

Field-specific reporting

Please select the one below that is the best fit for your research. If you are not sure, read the appropriate sections before making your selection.

☒ Life sciences ☐ Behavioural & social sciences ☐ Ecological, evolutionary & environmental sciences

For a reference copy of the document with all sections, see nature.com/documents/nr-reporting-summary-flat.pdf

Life sciences study design

All studies must disclose on these points even when the disclosure is negative.

Sample size	No statistical methods were used to determine sample size. Sample size was based on the number of individuals available (see Supplementary Tables 1 and 2).
Data exclusions	Low quality cells were excluded as described in Methods.
Replication	We generated 2 biological replicates for all collected brain samples, with the only exception of the larval diencephalon for which we generated 3 biological replicates (see Supplementary Table 2)
Randomization	Randomization was not used in this study.
Blinding	Blinding was not relevant to our study. Both data collection and analyses required an understanding of the nature of the sample being collected/analyzed.

Reporting for specific materials, systems and methods

We require information from authors about some types of materials, experimental systems and methods used in many studies. Here, indicate whether each material, system or method listed is relevant to your study. If you are not sure if a list item applies to your research, read the appropriate section before selecting a response.

Materials & experimental systems

n/a	Involved in the study	
☒	☐	Antibodies
☒	☐	Eukaryotic cell lines
☒	☐	Palaeontology and archaeology
☐	☒	Animals and other organisms
☒	☐	Human research participants
☒	☐	Clinical data
☒	☐	Dual use research of concern

Methods

n/a	Involved in the study	
☒	☐	ChIP-seq
☒	☐	Flow cytometry
☒	☐	MRI-based neuroimaging

Animals and other organisms

Policy information about studies involving animals; ARRIVE guidelines recommended for reporting animal research

Laboratory animals	Not used
Wild animals	Sea lamprey (Petromyzon marinus)
Field-collected samples	Sea lamprey (Petromyzon marinus)
Ethics oversight	All procedures involving animal care and experimentation were approved by: University of Colorado, Boulder Institutional Animal Care and Use Committee as described in protocol 2392; Regierungspräsidium Karlsruhe; Bioethics Committee of the University of Santiago de Compostela and the Xunta de Galicia Government; California Institute of Technology Institutional Animal Care and Use Committee (IACUC) protocol 1436.

Note that full information on the approval of the study protocol must also be provided in the manuscript.
