# Supplementary figures and images for: A lamprey neural cell type atlas illuminates the origins of the vertebrate brain
Source: Nat Ecol Evol. 2023 Sep 14;7(10):1714–28. doi: 10.1038/s41559-023-02170-1 (PMC10555824; doi:10.1038/s41559-023-02170-1)

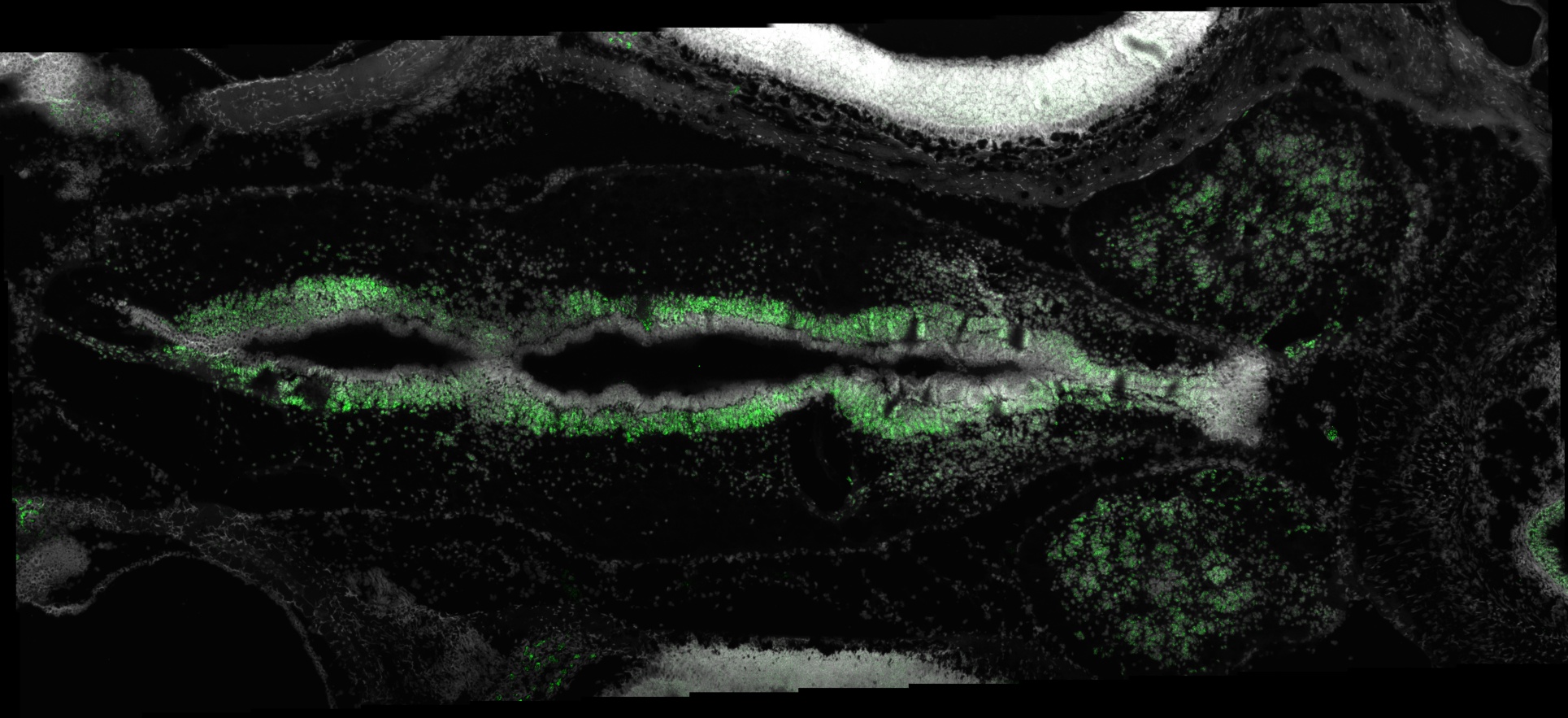

Supplement: Supplementary file 6 — In situ images produced in this study. [file 41559_2023_2170_MOESM6_ESM.zip › smFISH/Hulu_zfp704.jpg]

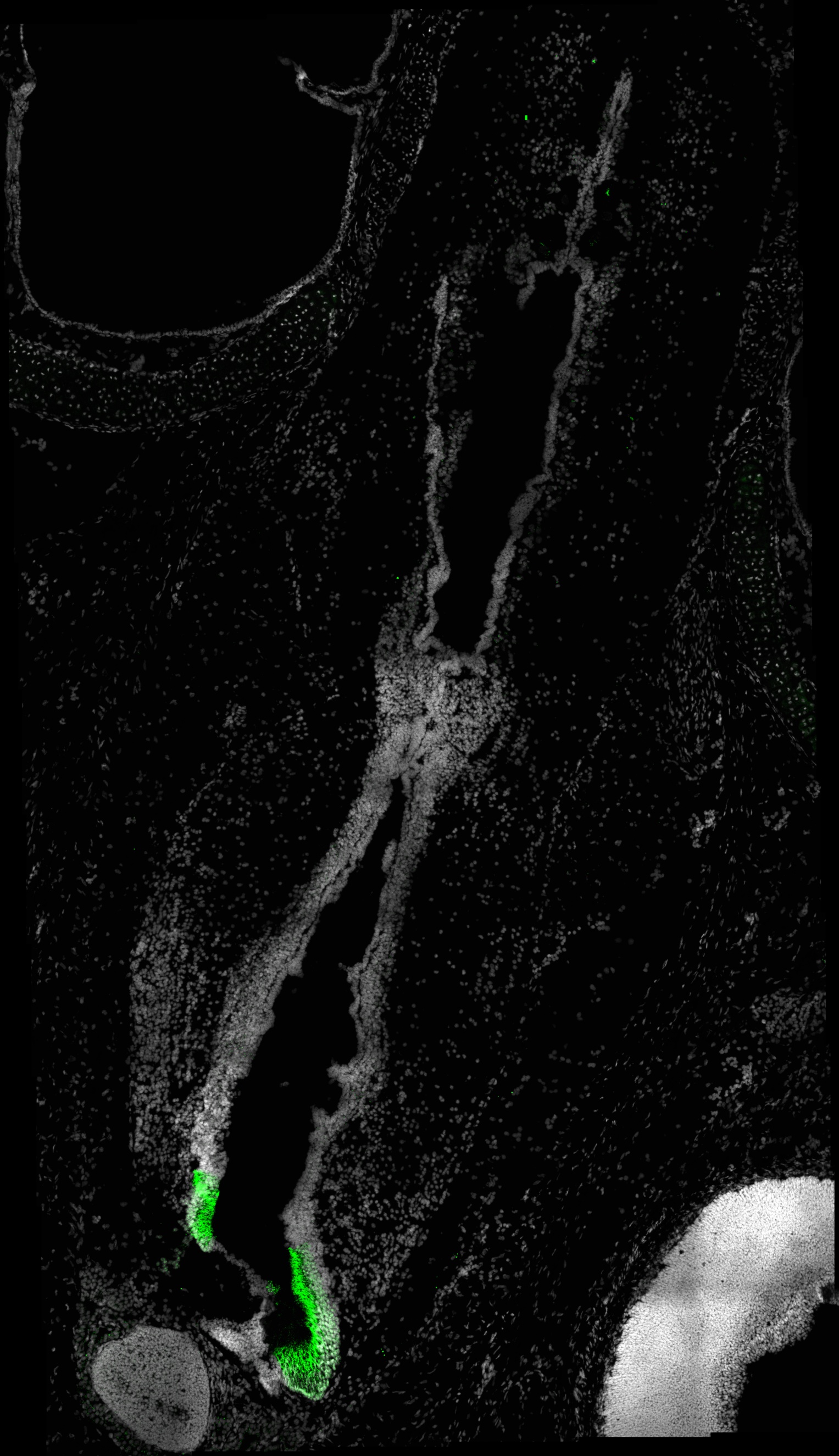

Supplement: Supplementary file 6 — In situ images produced in this study. [file 41559_2023_2170_MOESM6_ESM.zip › smFISH/Hulu_sspo.jpg]

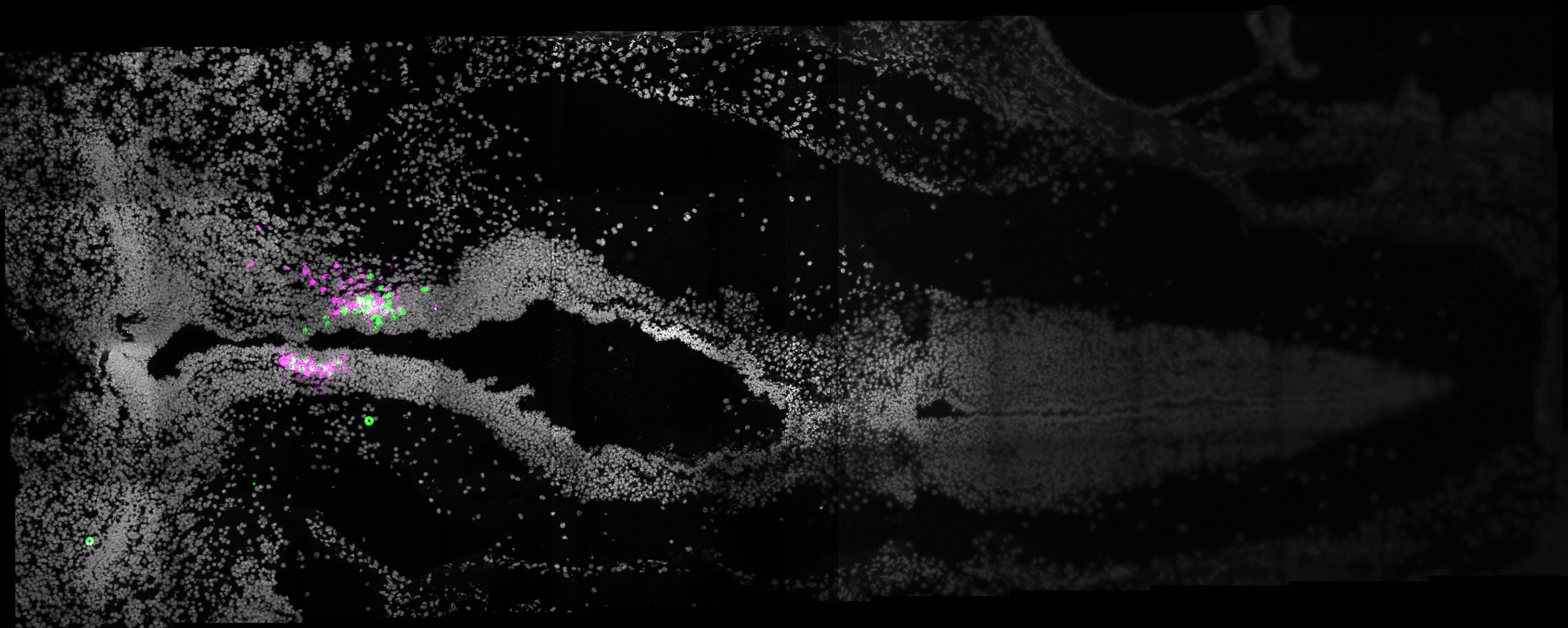

Supplement: Supplementary file 6 — In situ images produced in this study. [file 41559_2023_2170_MOESM6_ESM.zip › smFISH/Hulu_vat_gnrh1a.jpg]

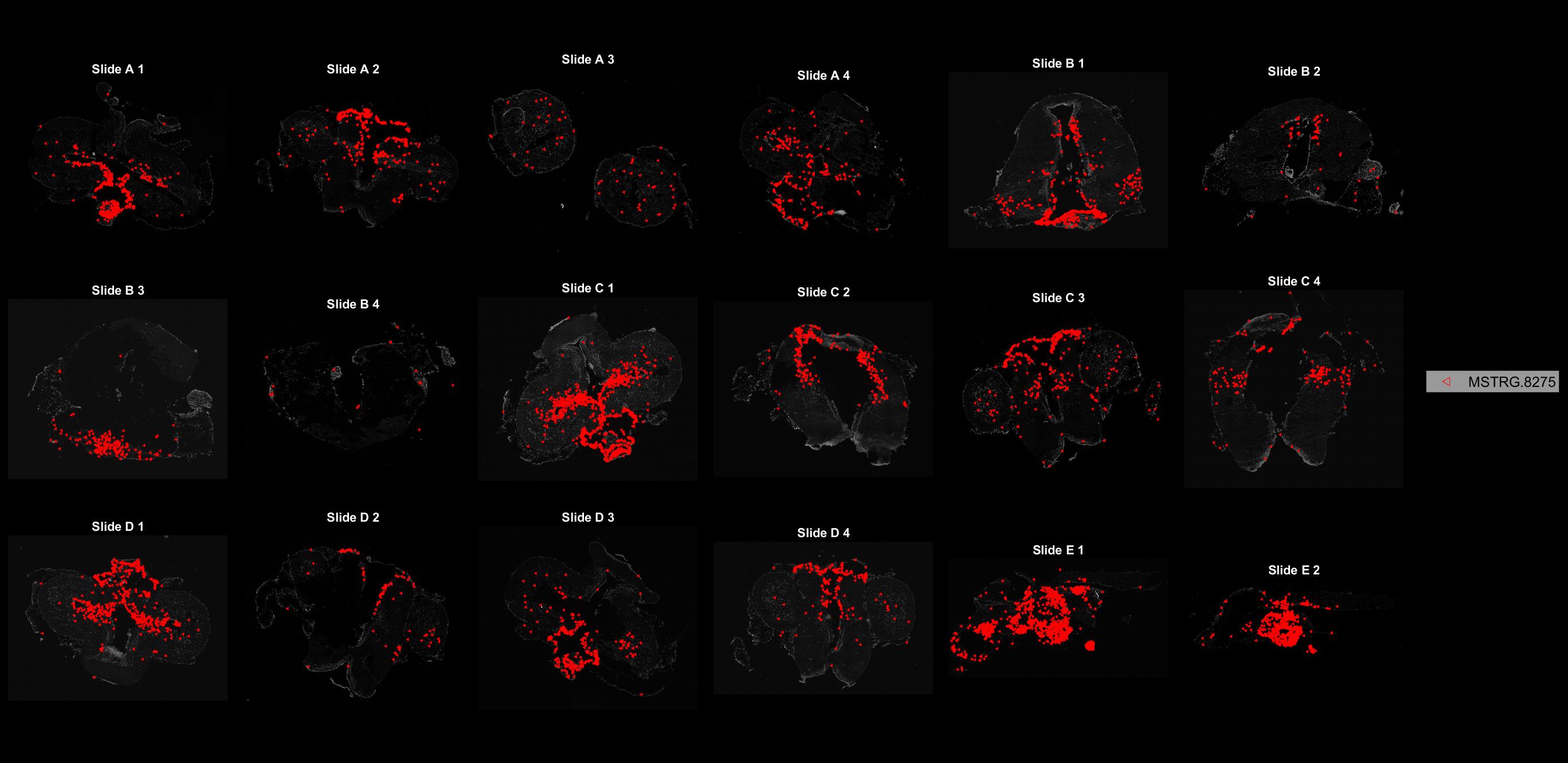

Supplement: Supplementary file 6 — In situ images produced in this study. [file 41559_2023_2170_MOESM6_ESM.zip › ISS/MSTRG.8275.jpg]

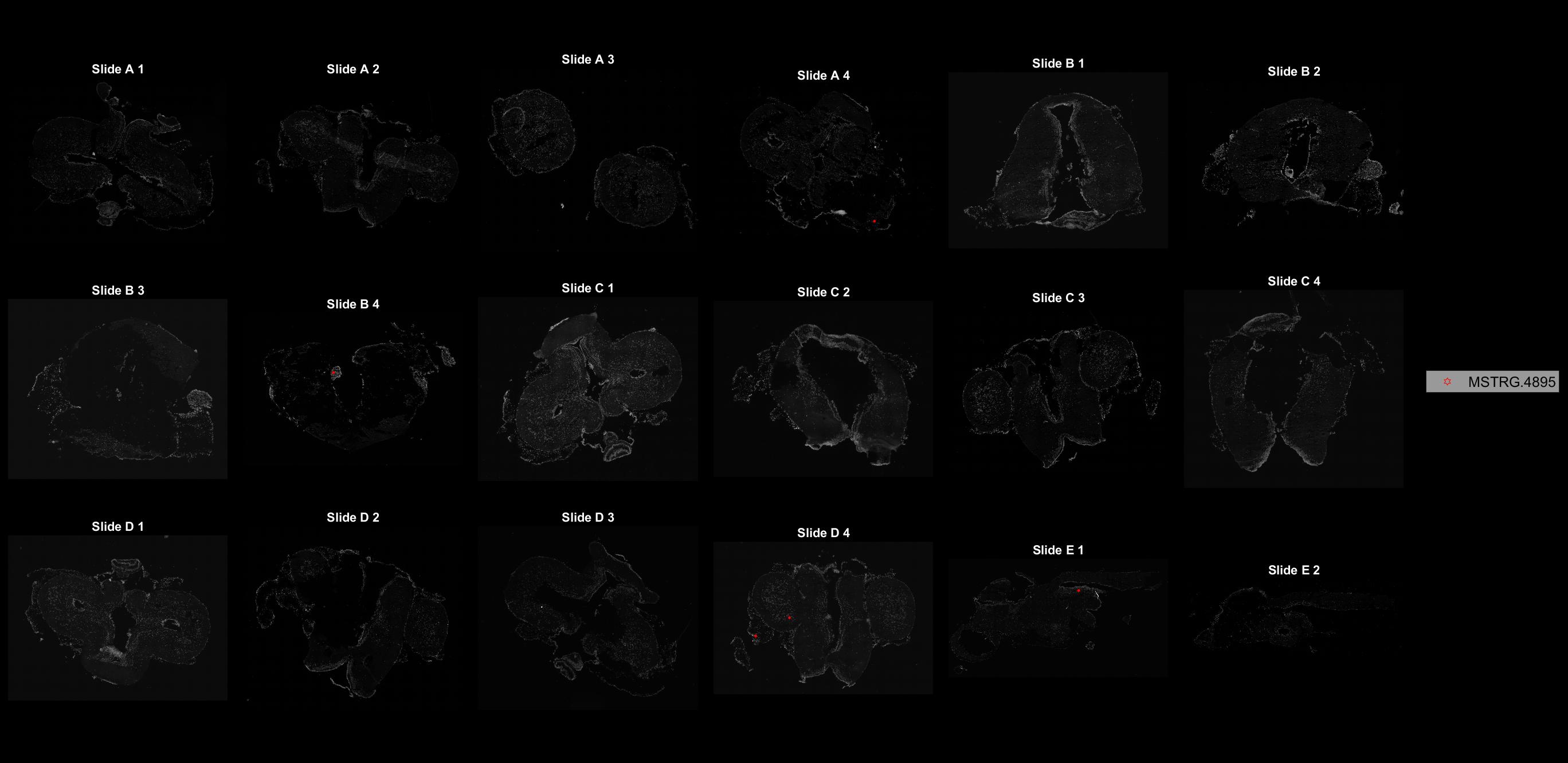

Supplement: Supplementary file 6 — In situ images produced in this study. [file 41559_2023_2170_MOESM6_ESM.zip › ISS/MSTRG.4895.jpg]

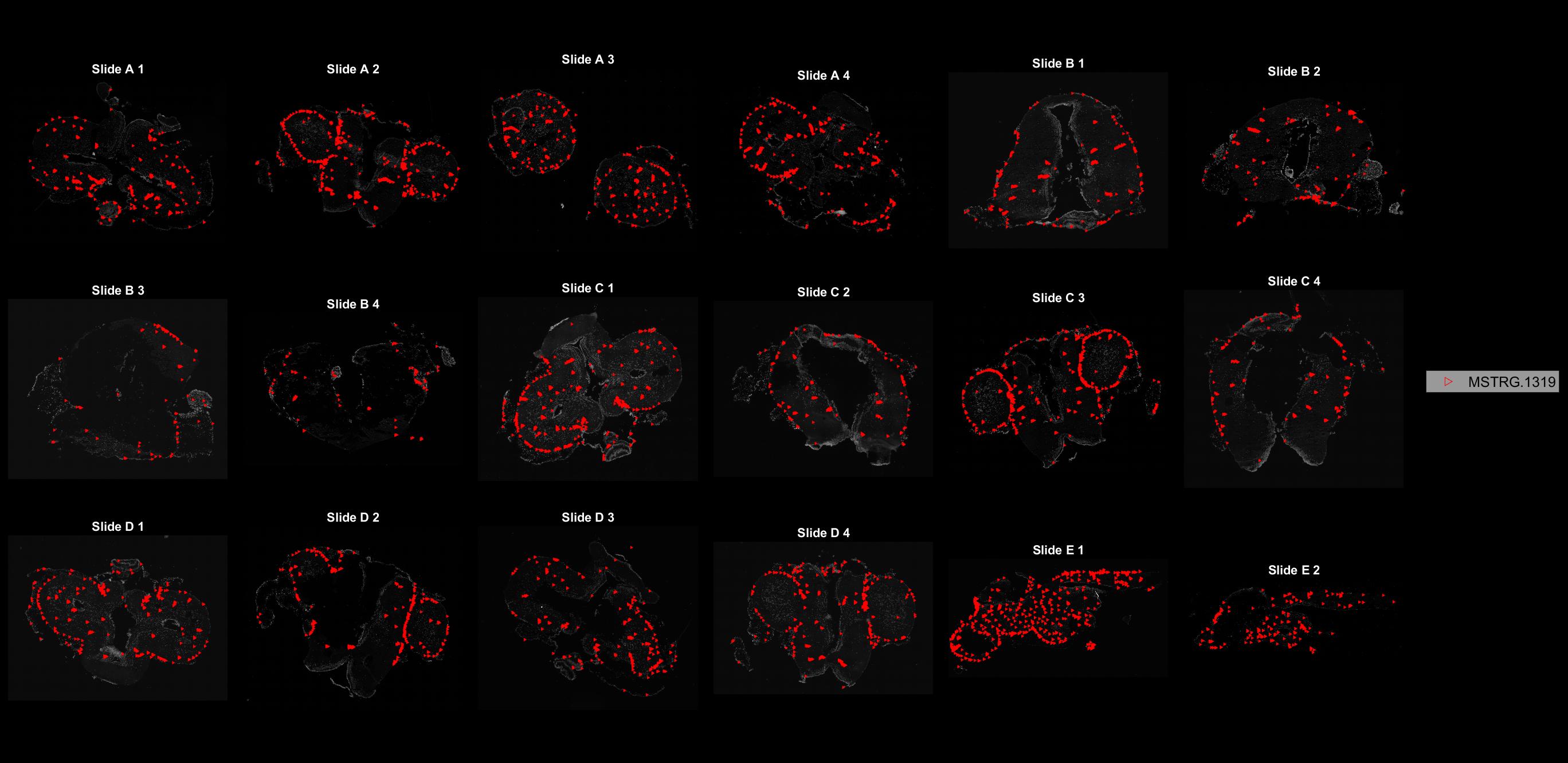

Supplement: Supplementary file 6 — In situ images produced in this study. [file 41559_2023_2170_MOESM6_ESM.zip › ISS/MSTRG.1319.jpg]

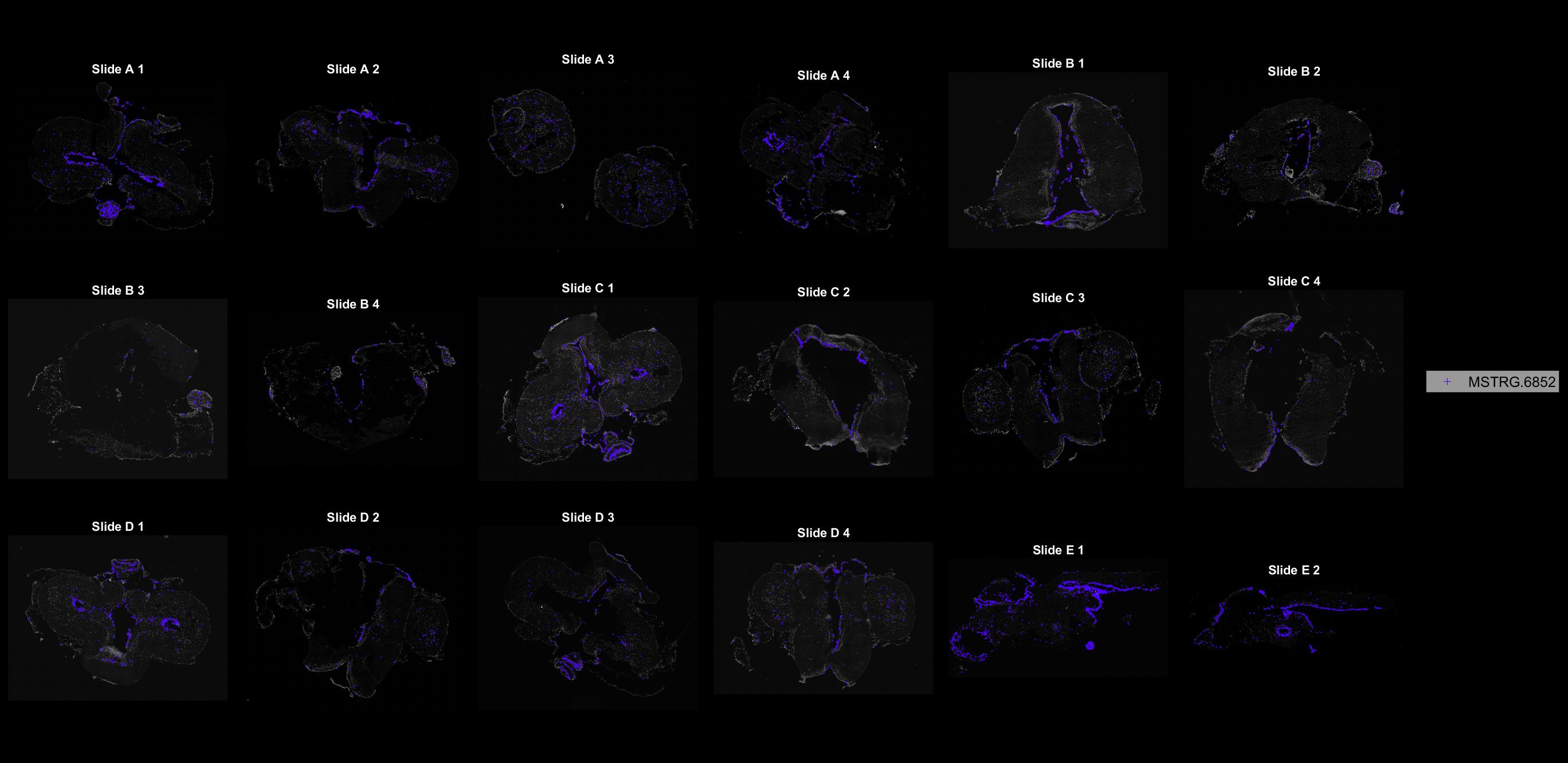

Supplement: Supplementary file 6 — In situ images produced in this study. [file 41559_2023_2170_MOESM6_ESM.zip › ISS/MSTRG.6852.jpg]

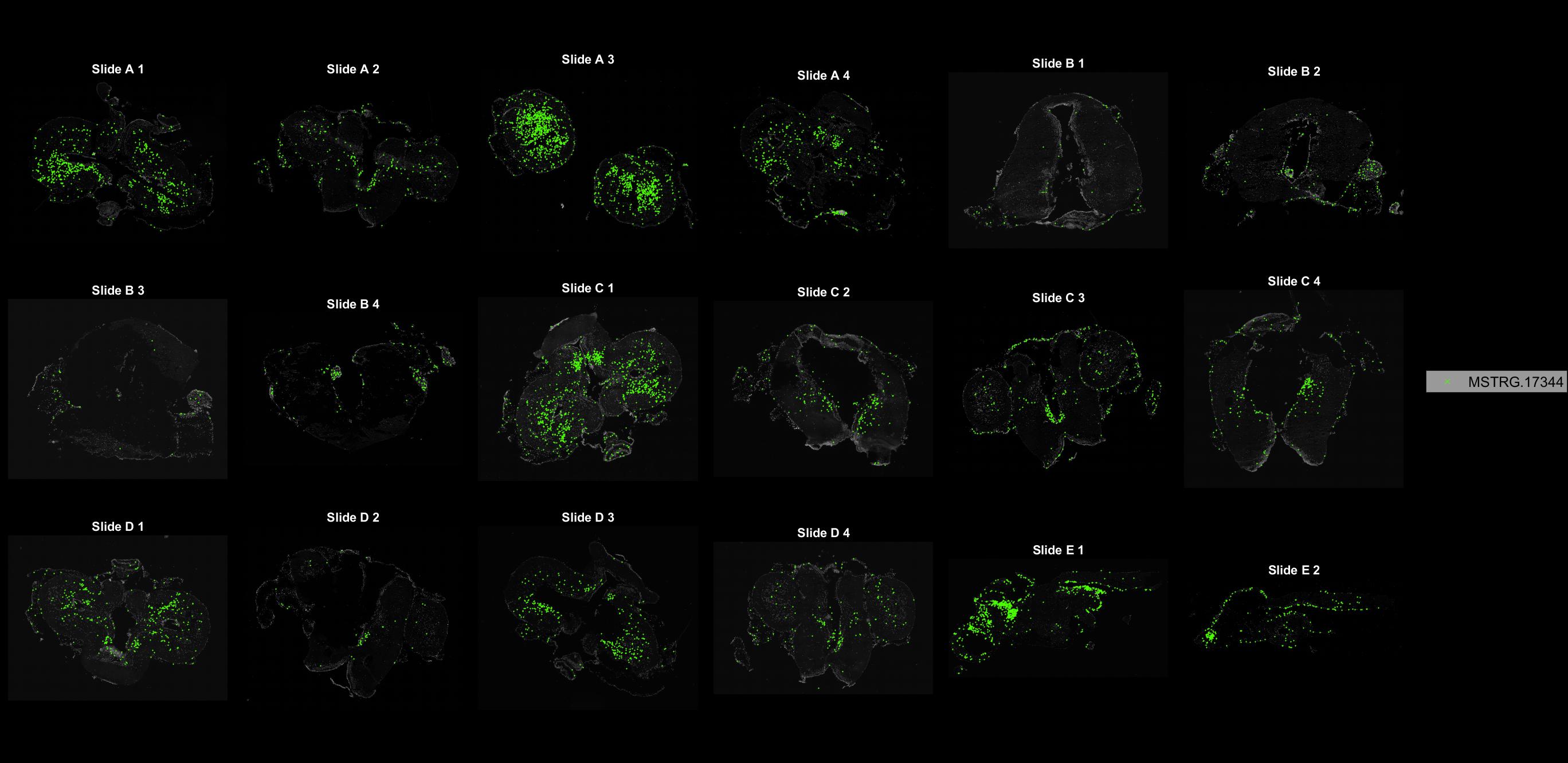

Supplement: Supplementary file 6 — In situ images produced in this study. [file 41559_2023_2170_MOESM6_ESM.zip › ISS/MSTRG.17344.jpg]

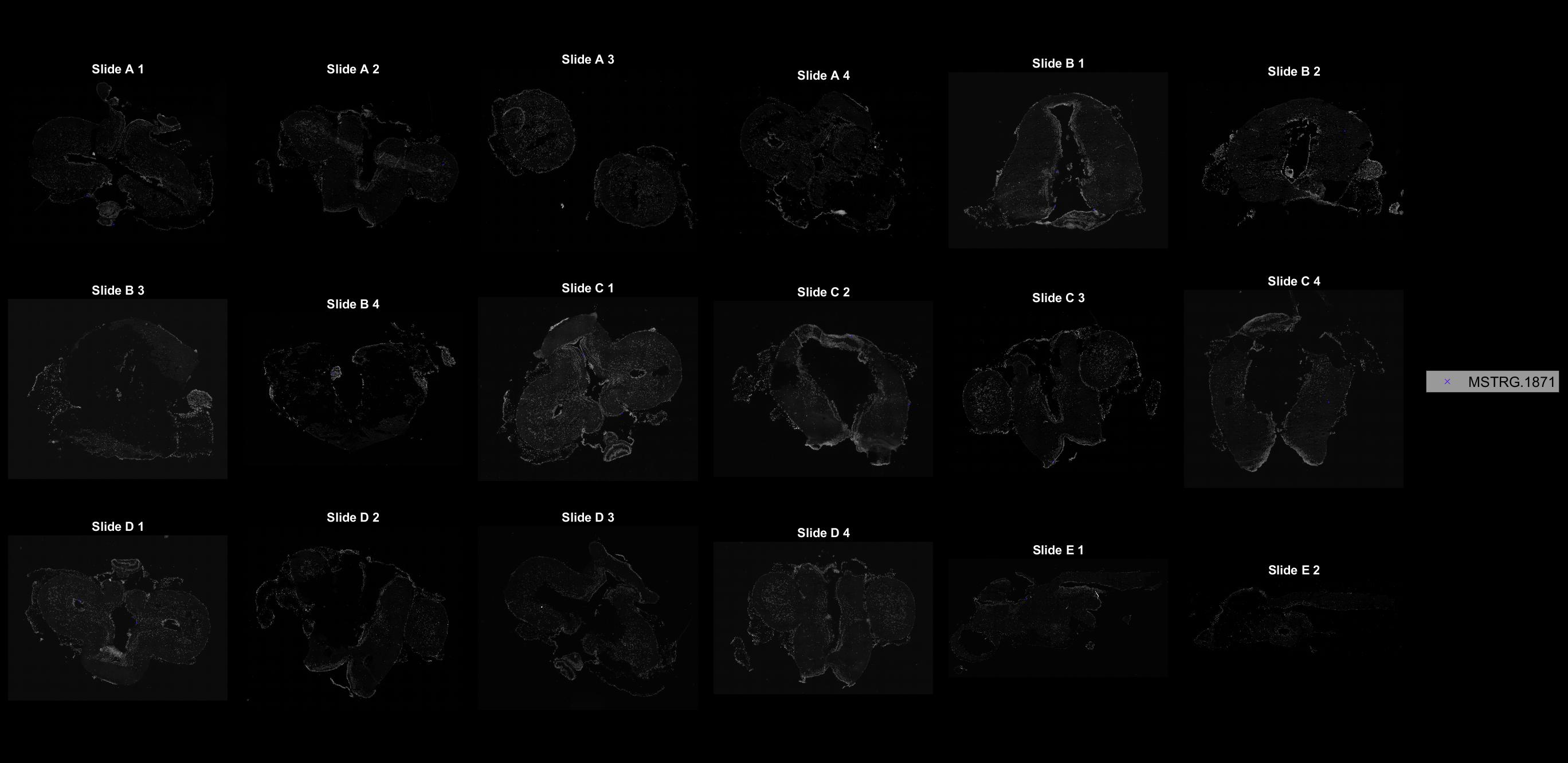

Supplement: Supplementary file 6 — In situ images produced in this study. [file 41559_2023_2170_MOESM6_ESM.zip › ISS/MSTRG.1871.jpg]

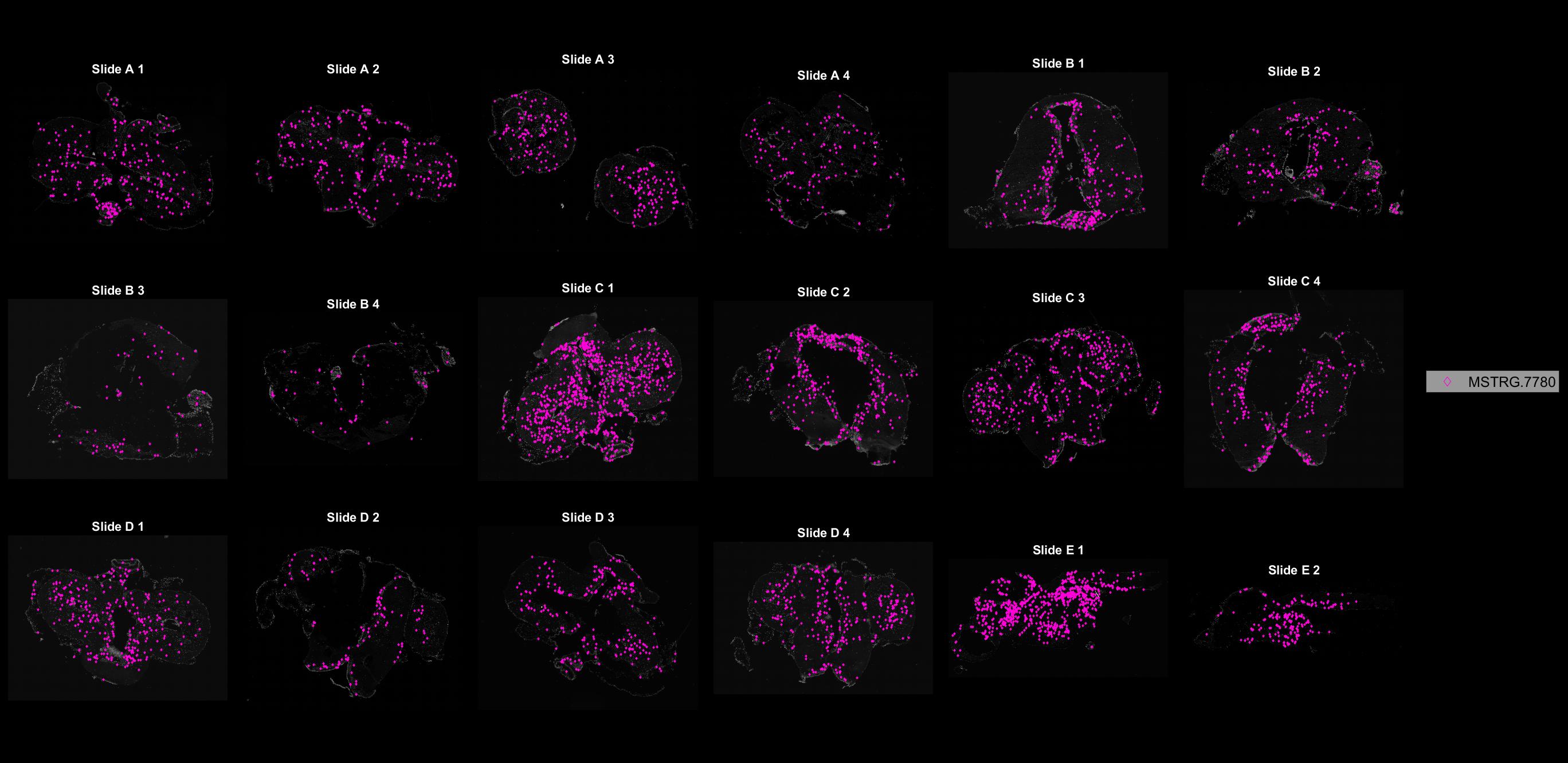

Supplement: Supplementary file 6 — In situ images produced in this study. [file 41559_2023_2170_MOESM6_ESM.zip › ISS/MSTRG.7780.jpg]

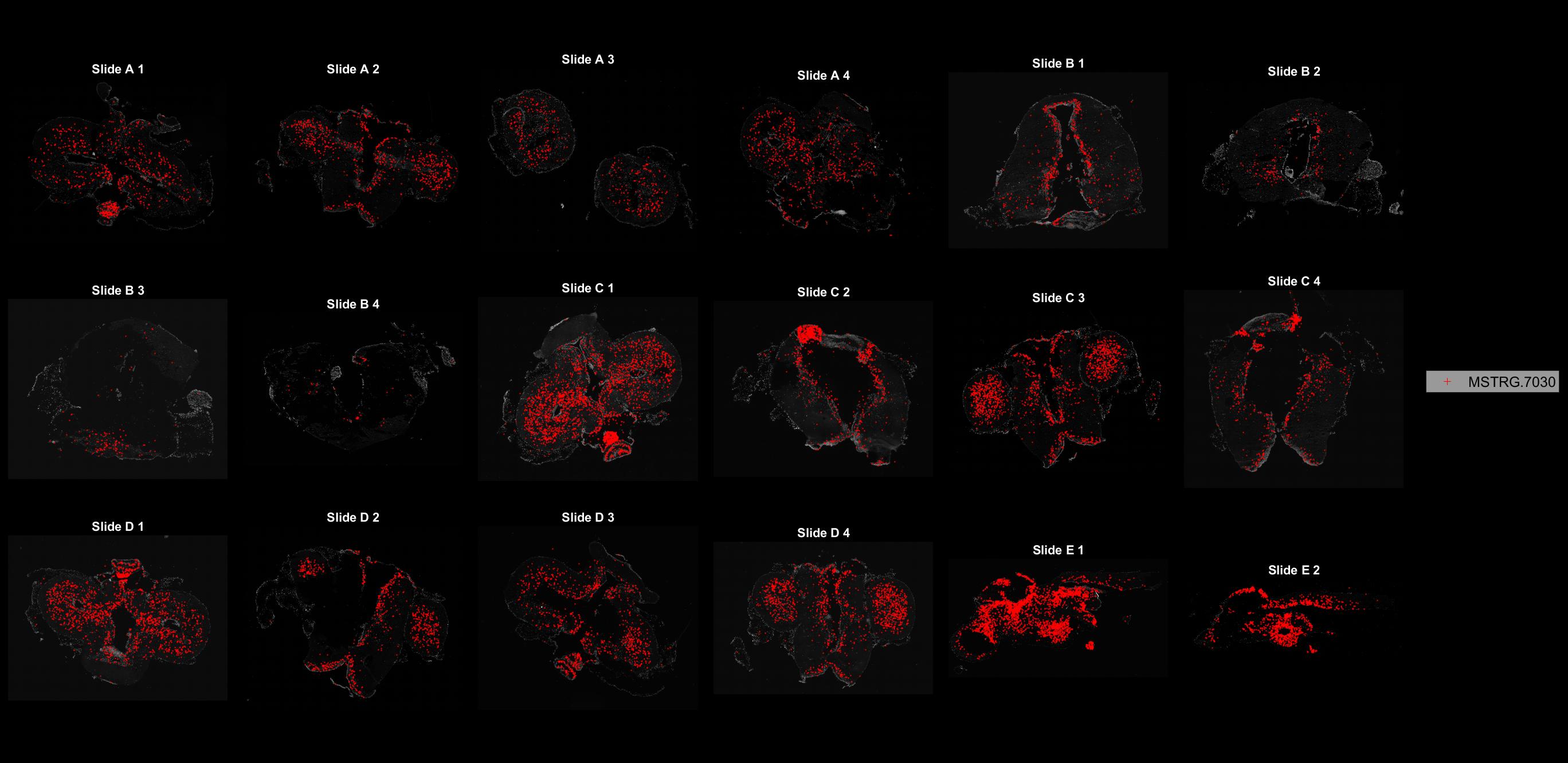

Supplement: Supplementary file 6 — In situ images produced in this study. [file 41559_2023_2170_MOESM6_ESM.zip › ISS/MSTRG.7030.jpg]

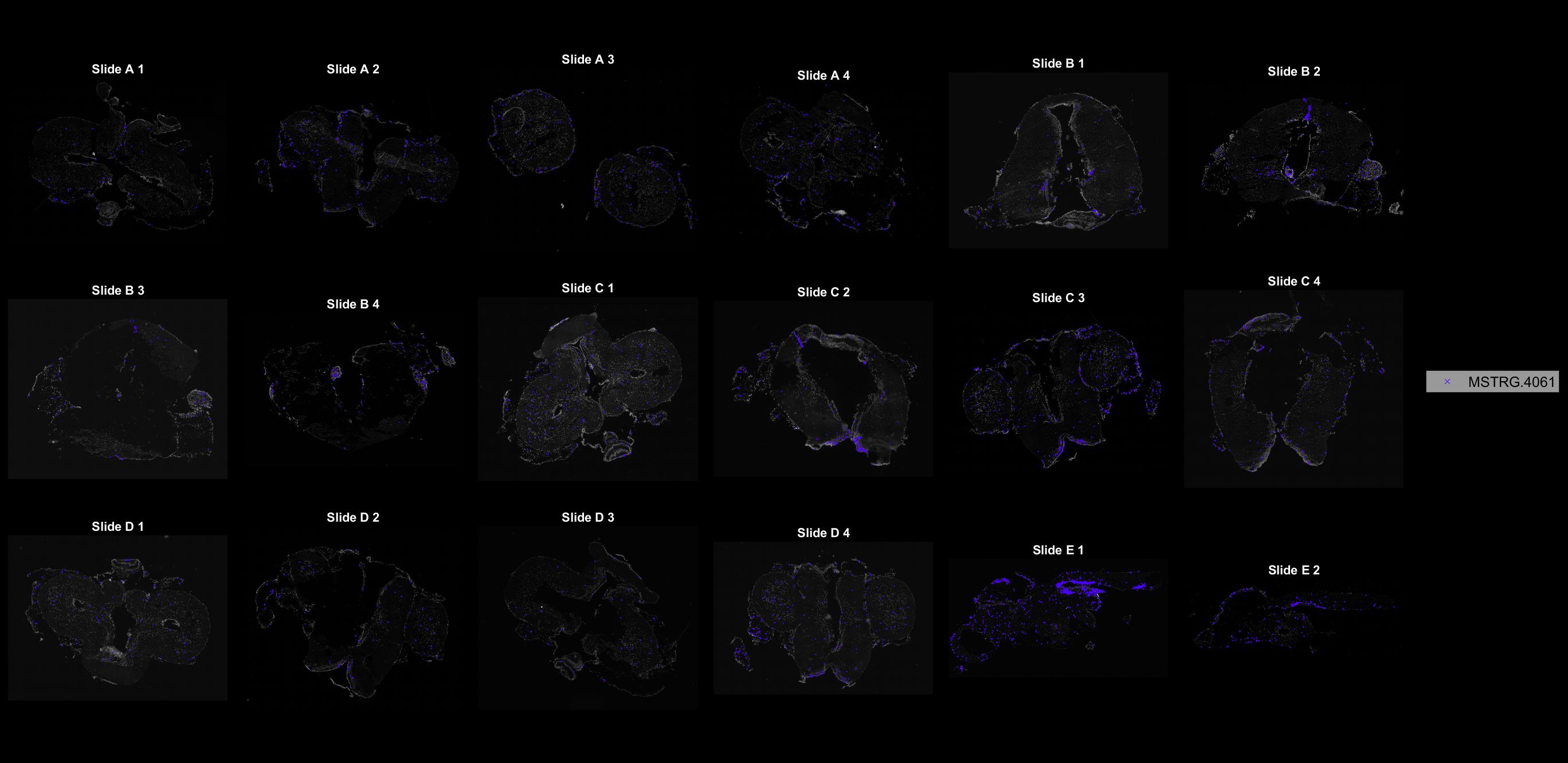

Supplement: Supplementary file 6 — In situ images produced in this study. [file 41559_2023_2170_MOESM6_ESM.zip › ISS/MSTRG.4061.jpg]

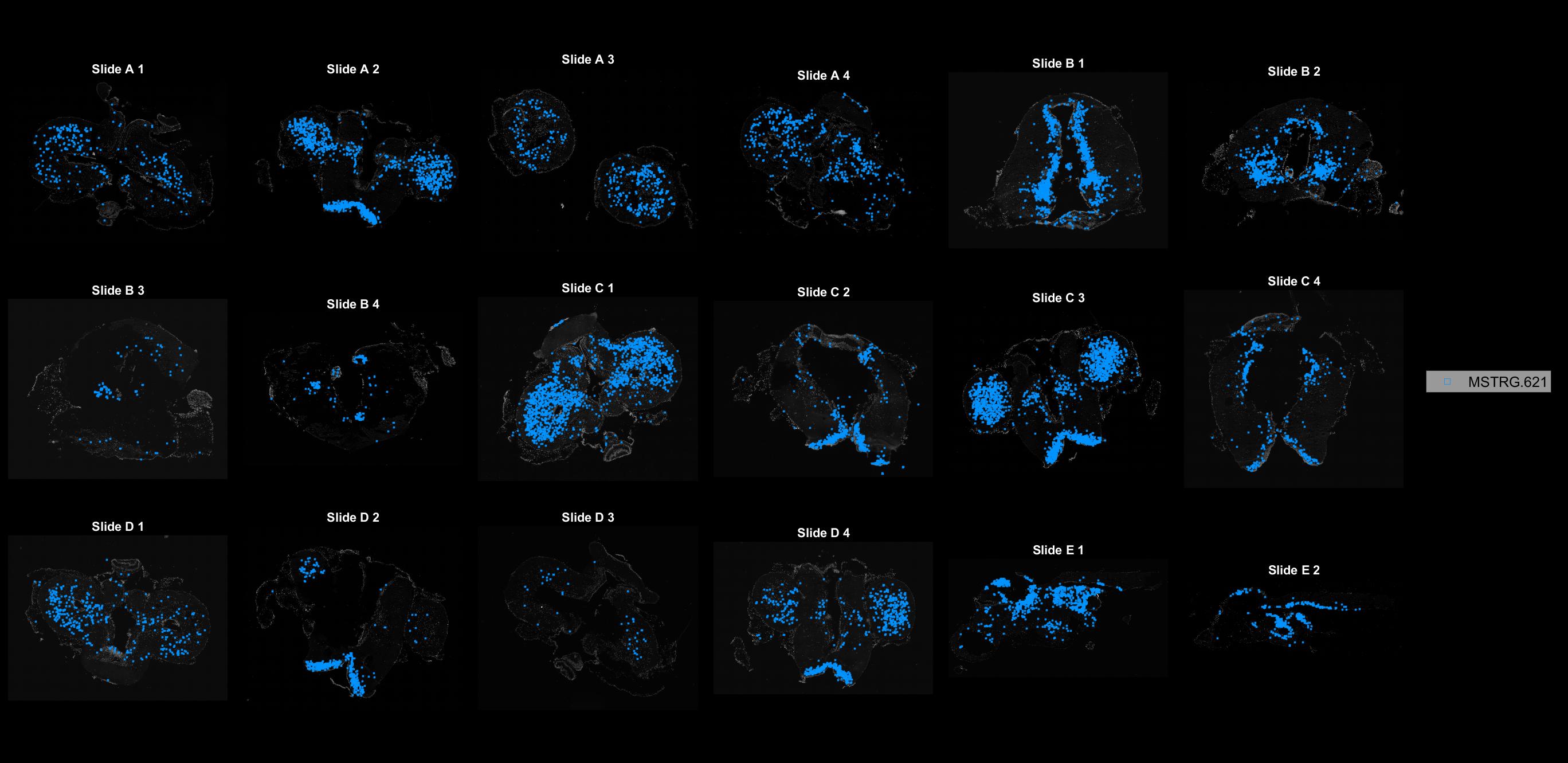

Supplement: Supplementary file 6 — In situ images produced in this study. [file 41559_2023_2170_MOESM6_ESM.zip › ISS/MSTRG.621.jpg]

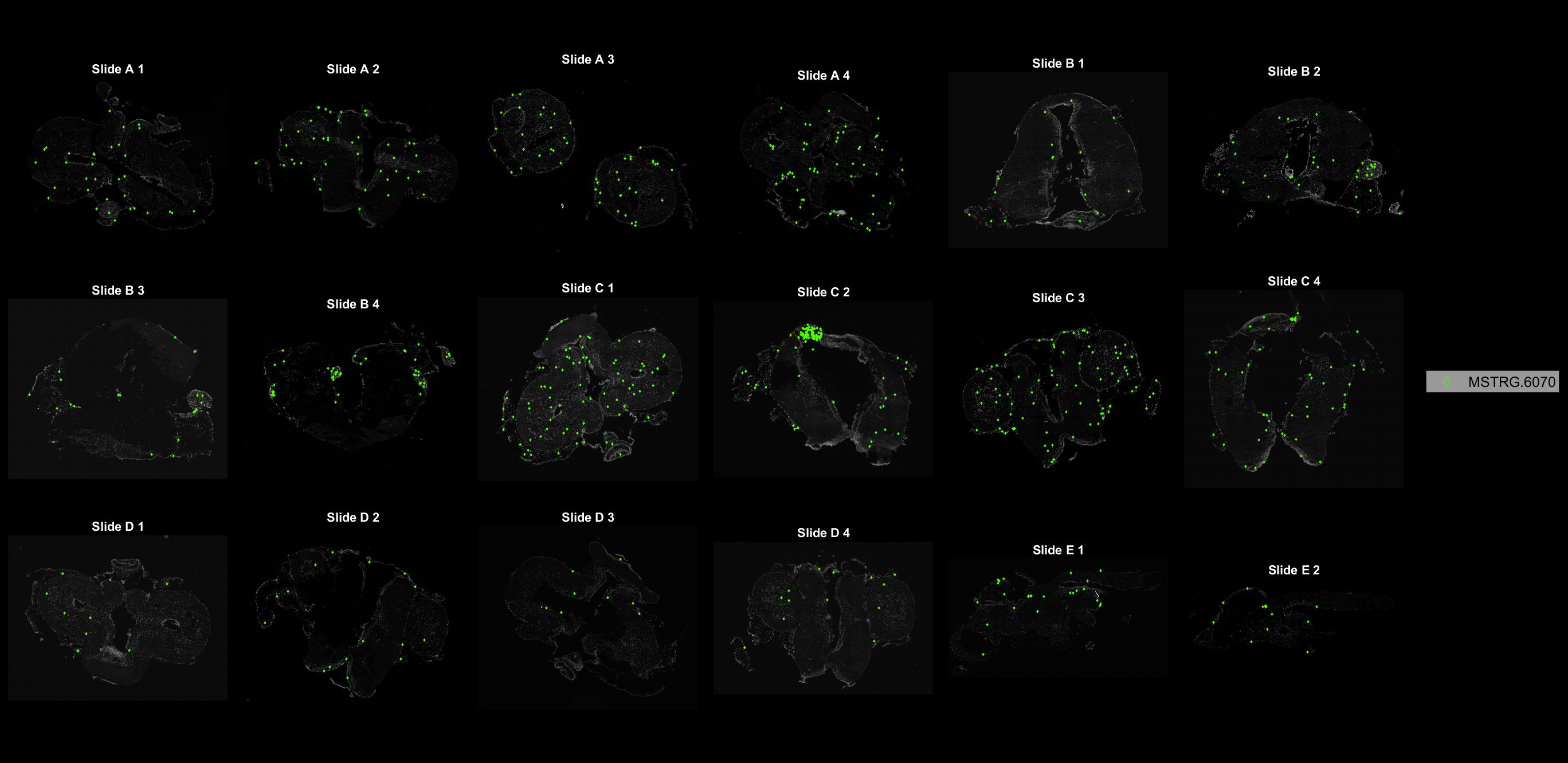

Supplement: Supplementary file 6 — In situ images produced in this study. [file 41559_2023_2170_MOESM6_ESM.zip › ISS/MSTRG.6070.jpg]

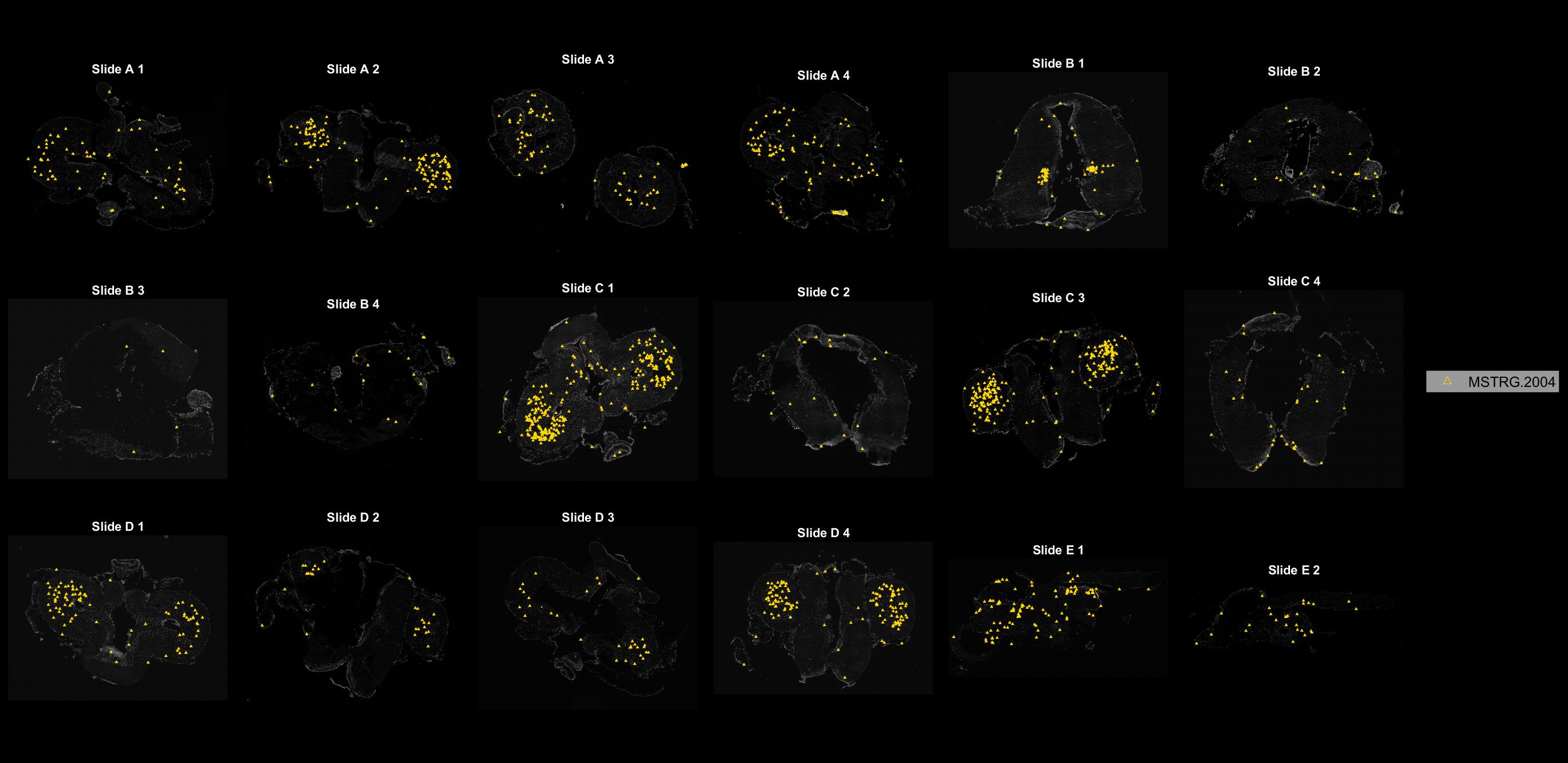

Supplement: Supplementary file 6 — In situ images produced in this study. [file 41559_2023_2170_MOESM6_ESM.zip › ISS/MSTRG.2004.jpg]

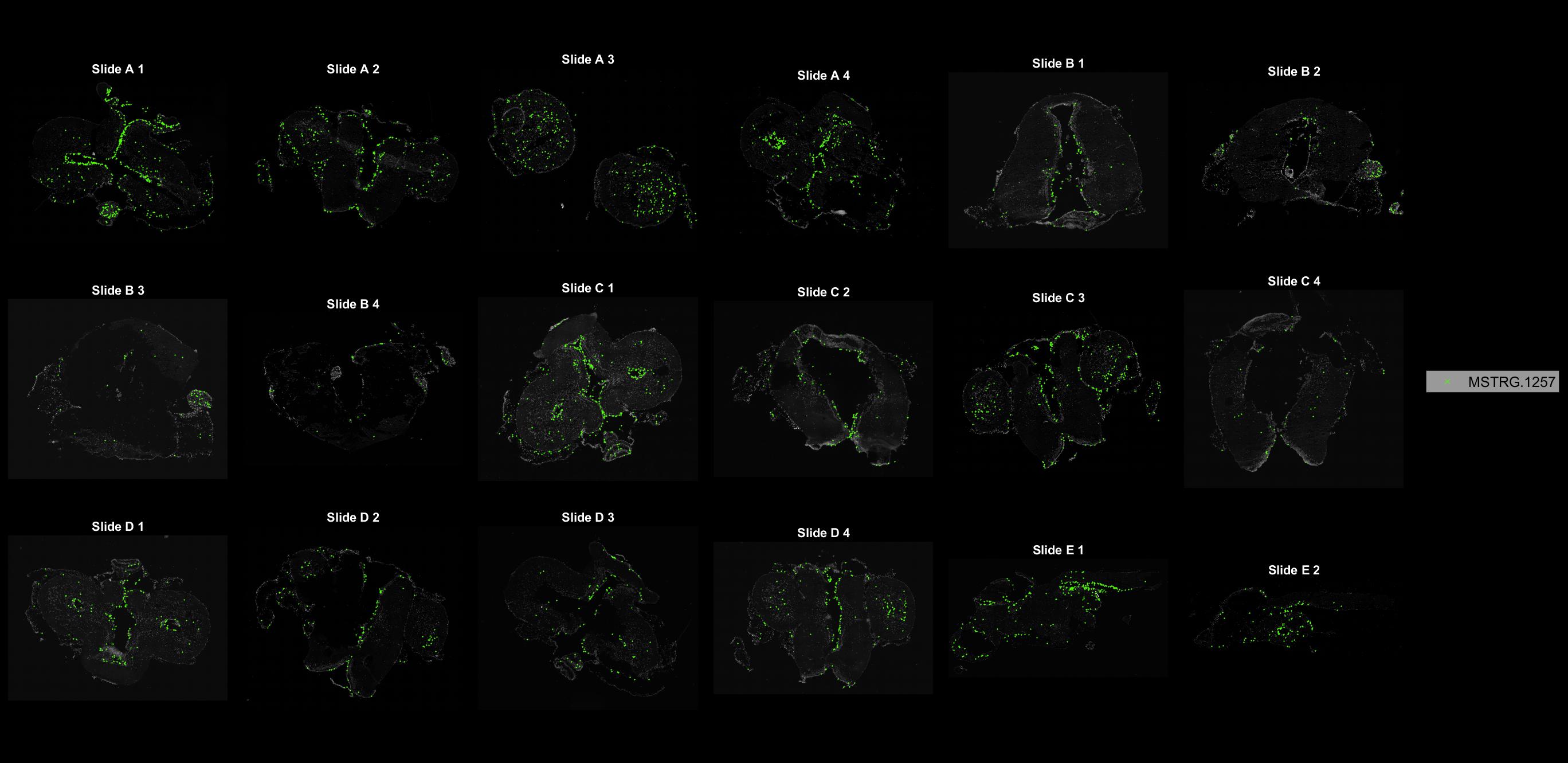

Supplement: Supplementary file 6 — In situ images produced in this study. [file 41559_2023_2170_MOESM6_ESM.zip › ISS/MSTRG.1257.jpg]

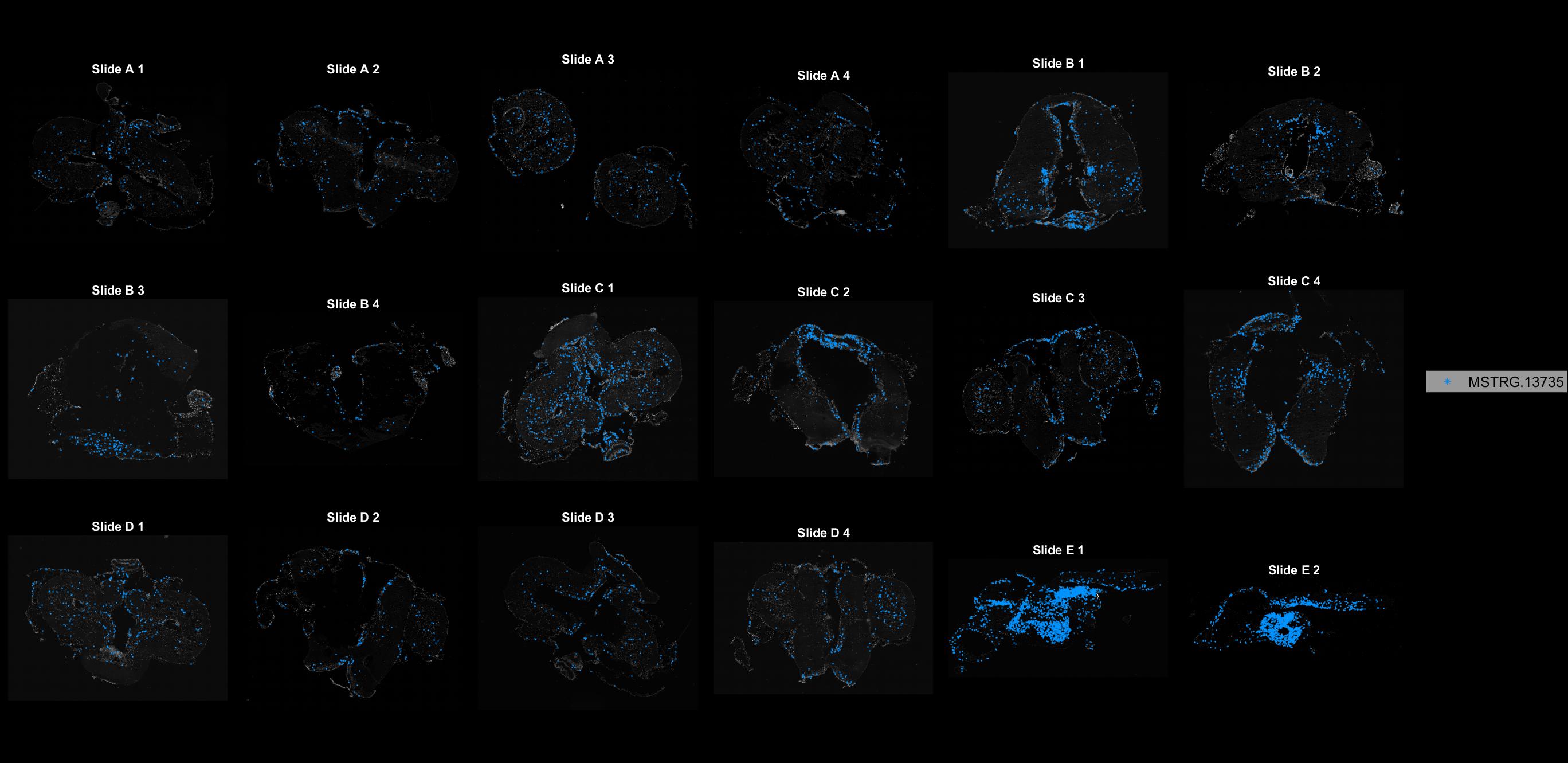

Supplement: Supplementary file 6 — In situ images produced in this study. [file 41559_2023_2170_MOESM6_ESM.zip › ISS/MSTRG.13735.jpg]

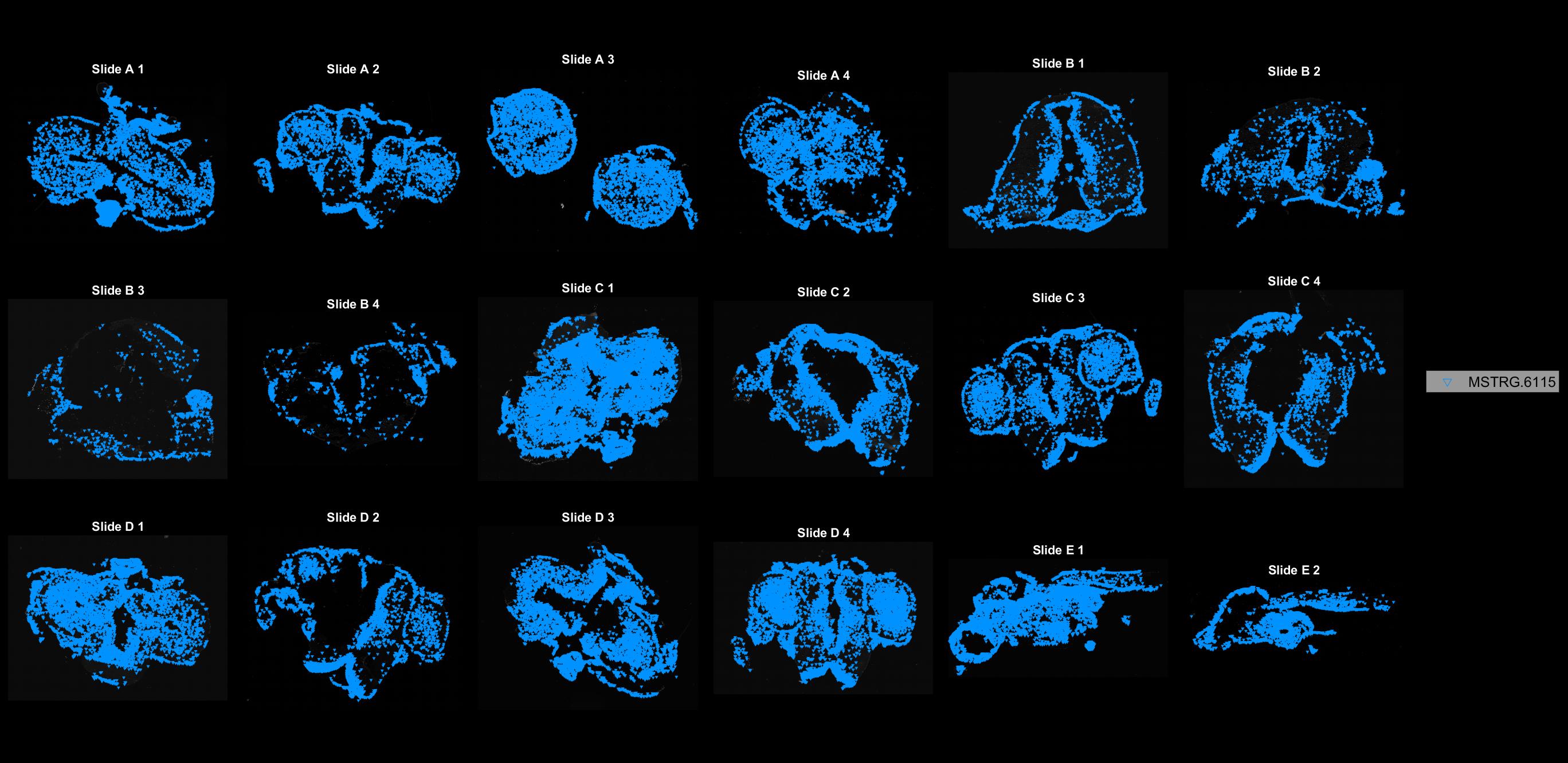

Supplement: Supplementary file 6 — In situ images produced in this study. [file 41559_2023_2170_MOESM6_ESM.zip › ISS/MSTRG.6115.jpg]

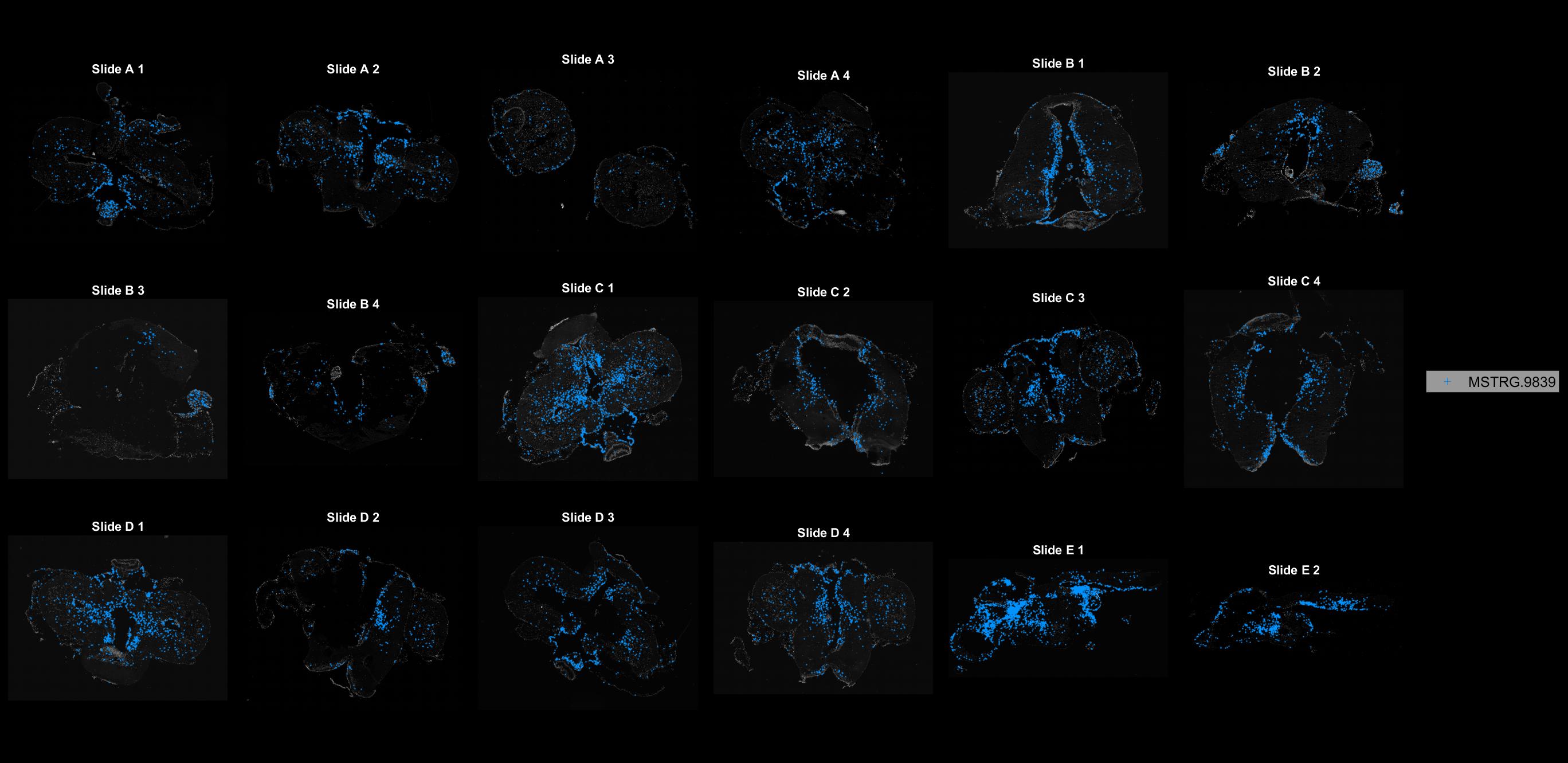

Supplement: Supplementary file 6 — In situ images produced in this study. [file 41559_2023_2170_MOESM6_ESM.zip › ISS/MSTRG.9839.jpg]

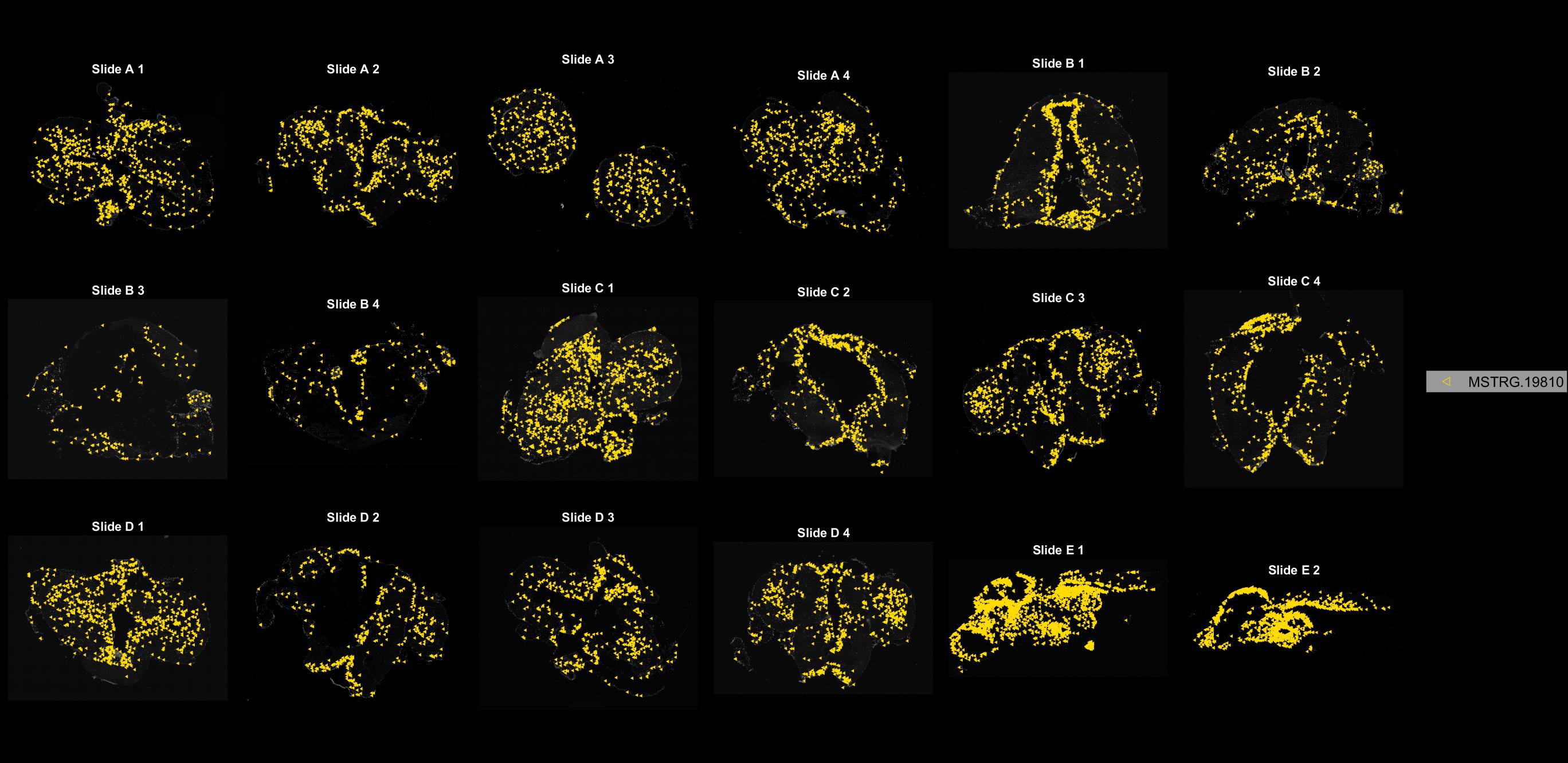

Supplement: Supplementary file 6 — In situ images produced in this study. [file 41559_2023_2170_MOESM6_ESM.zip › ISS/MSTRG.19810.jpg]

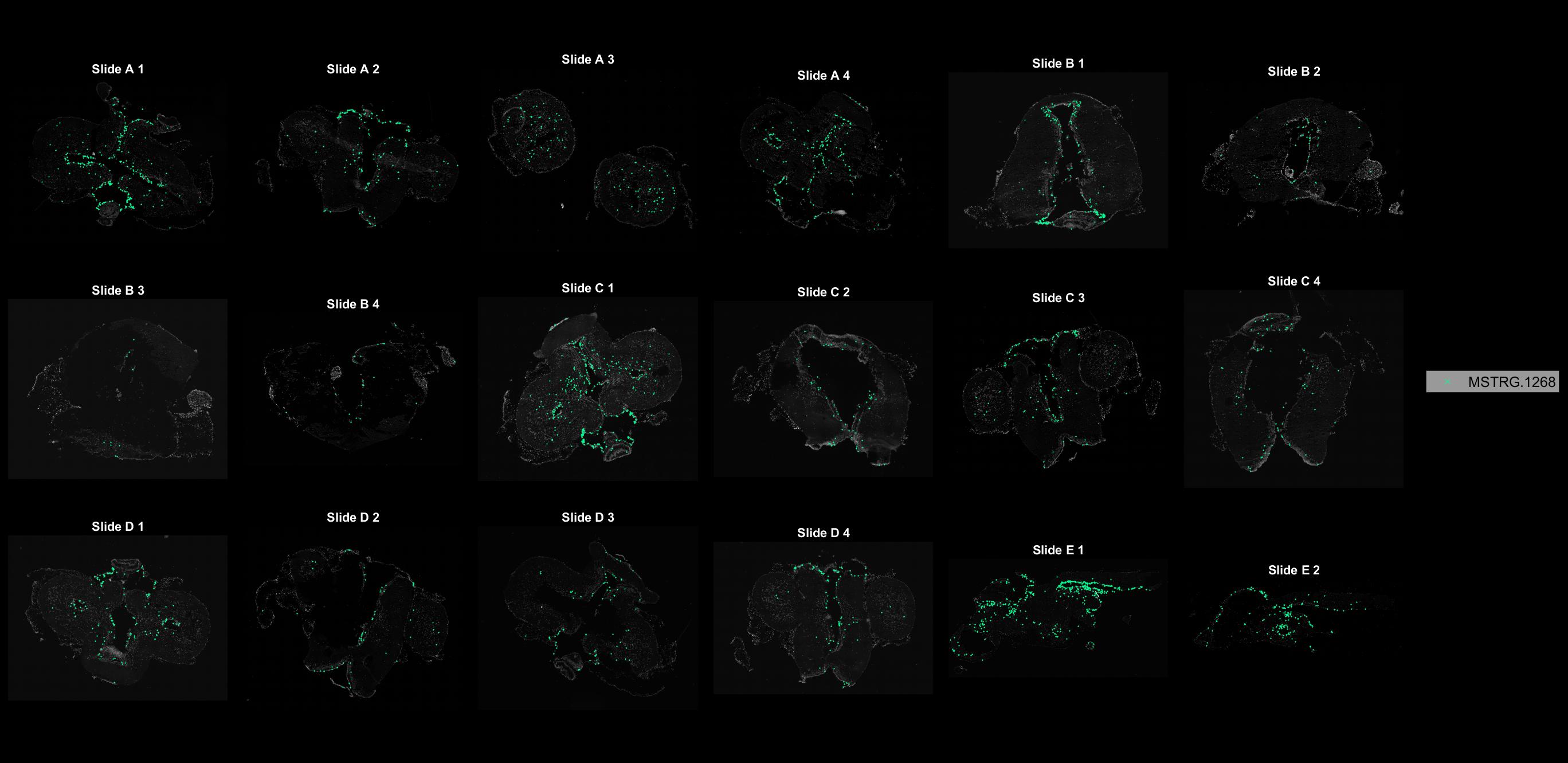

Supplement: Supplementary file 6 — In situ images produced in this study. [file 41559_2023_2170_MOESM6_ESM.zip › ISS/MSTRG.1268.jpg]

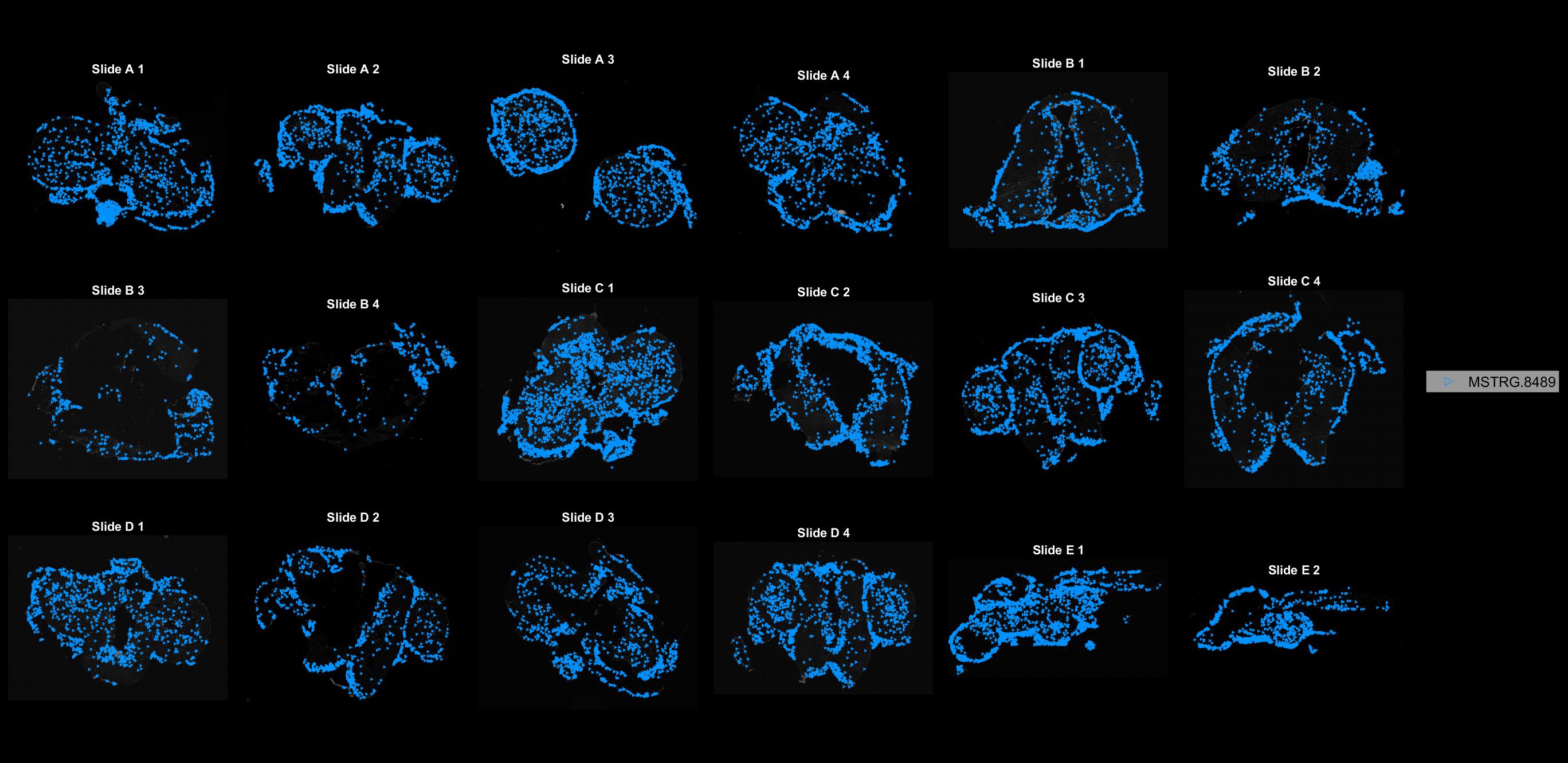

Supplement: Supplementary file 6 — In situ images produced in this study. [file 41559_2023_2170_MOESM6_ESM.zip › ISS/MSTRG.8489.jpg]

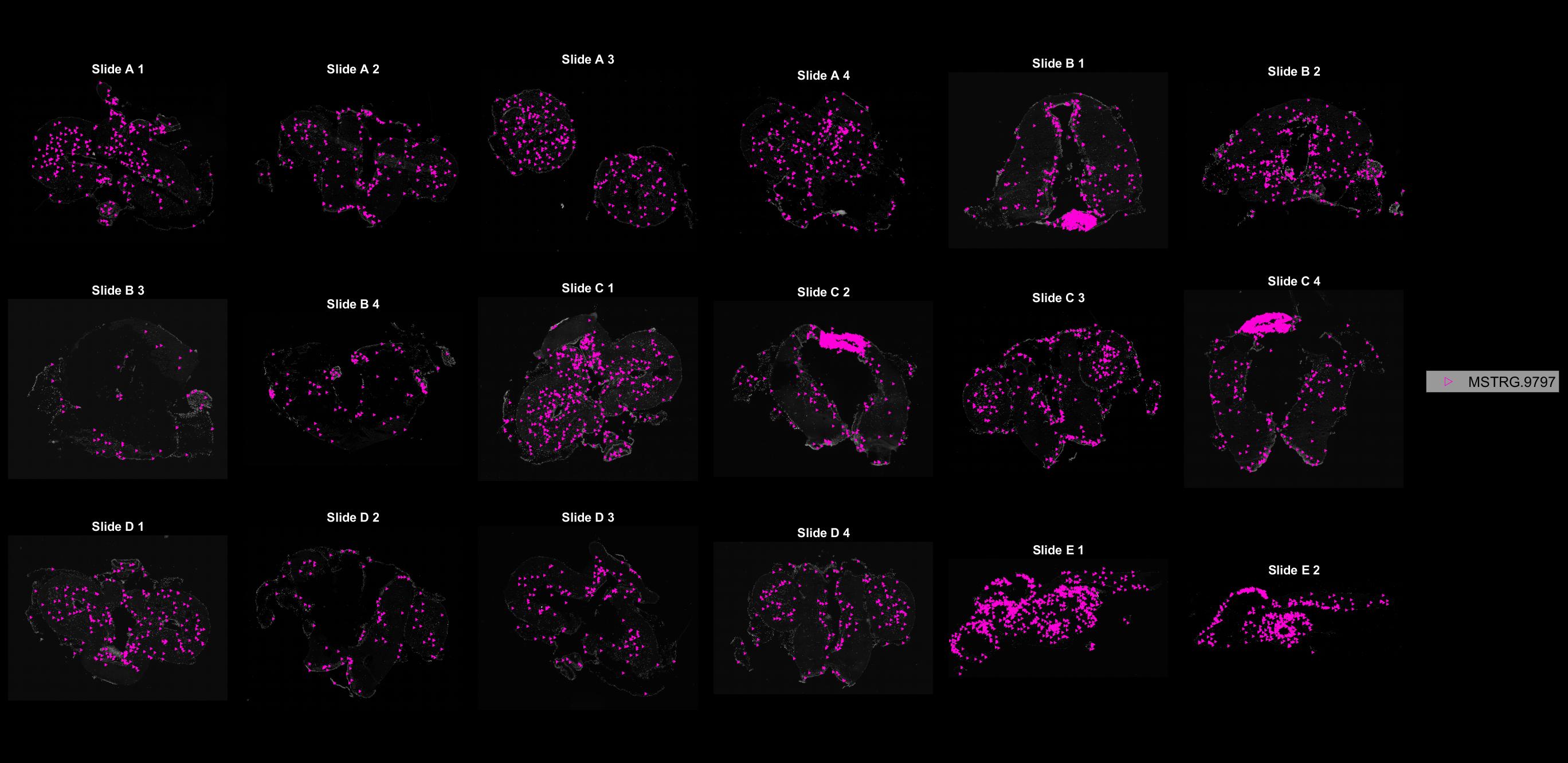

Supplement: Supplementary file 6 — In situ images produced in this study. [file 41559_2023_2170_MOESM6_ESM.zip › ISS/MSTRG.9797.jpg]

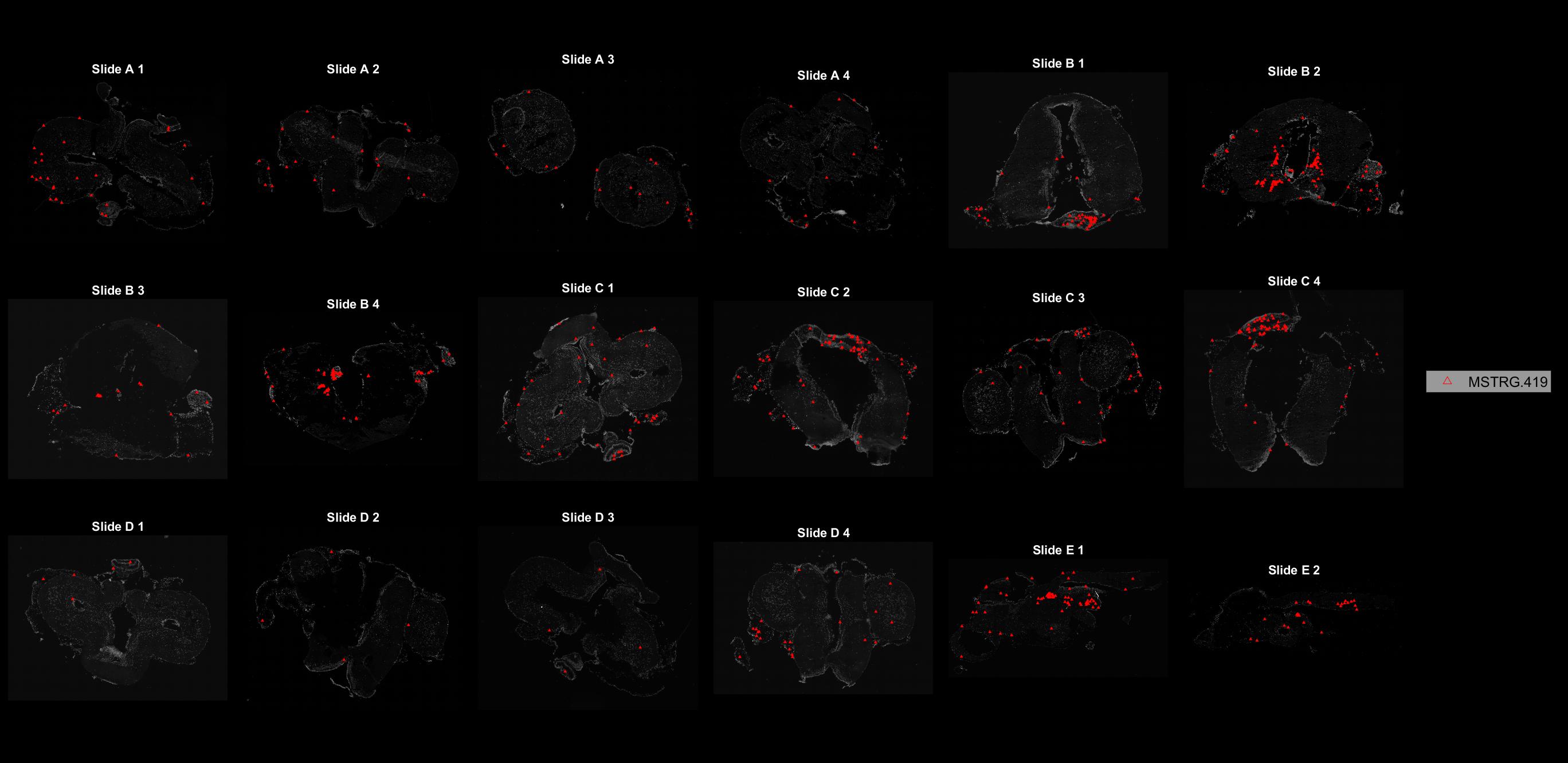

Supplement: Supplementary file 6 — In situ images produced in this study. [file 41559_2023_2170_MOESM6_ESM.zip › ISS/MSTRG.419.jpg]

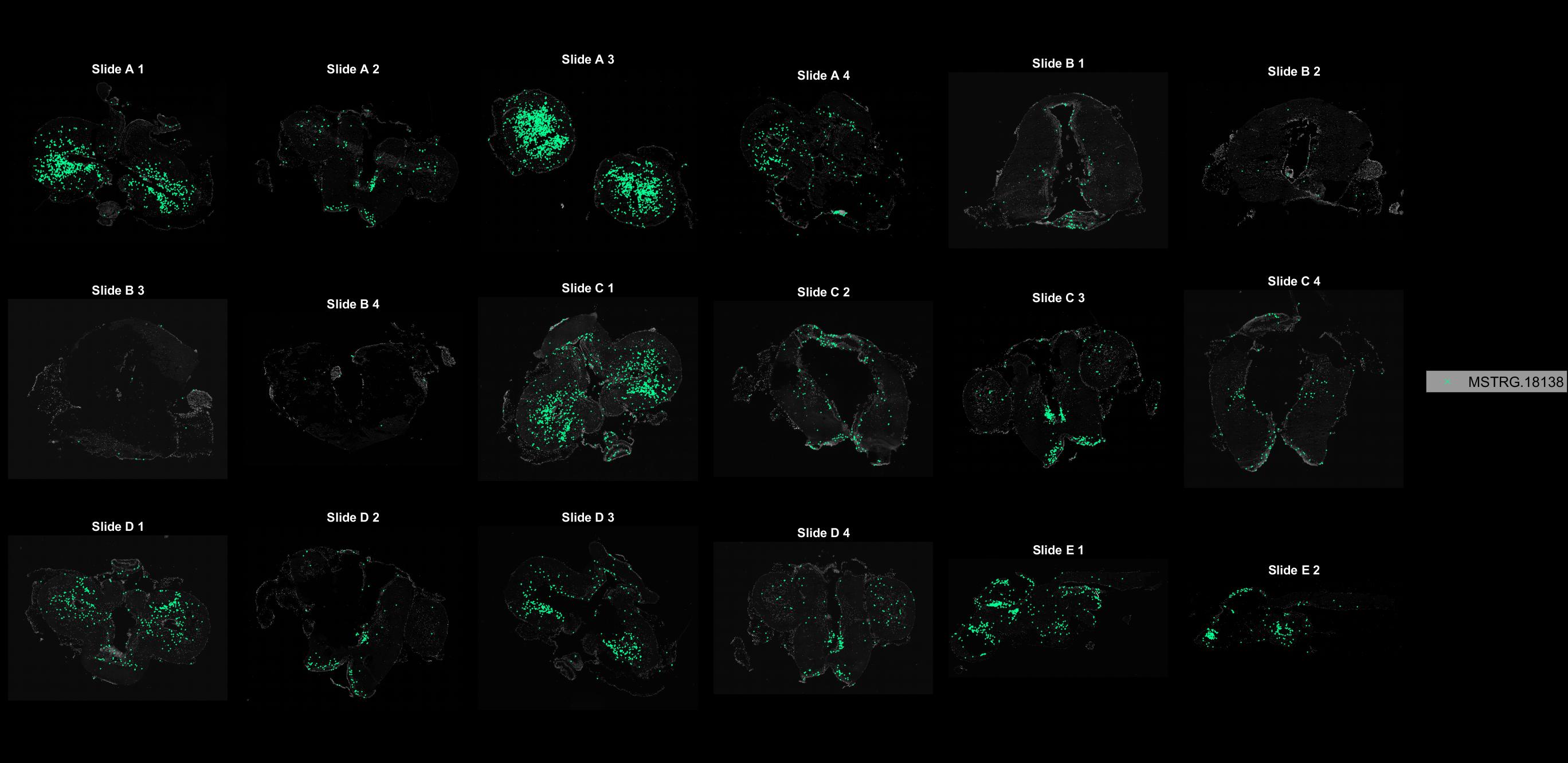

Supplement: Supplementary file 6 — In situ images produced in this study. [file 41559_2023_2170_MOESM6_ESM.zip › ISS/MSTRG.18138.jpg]

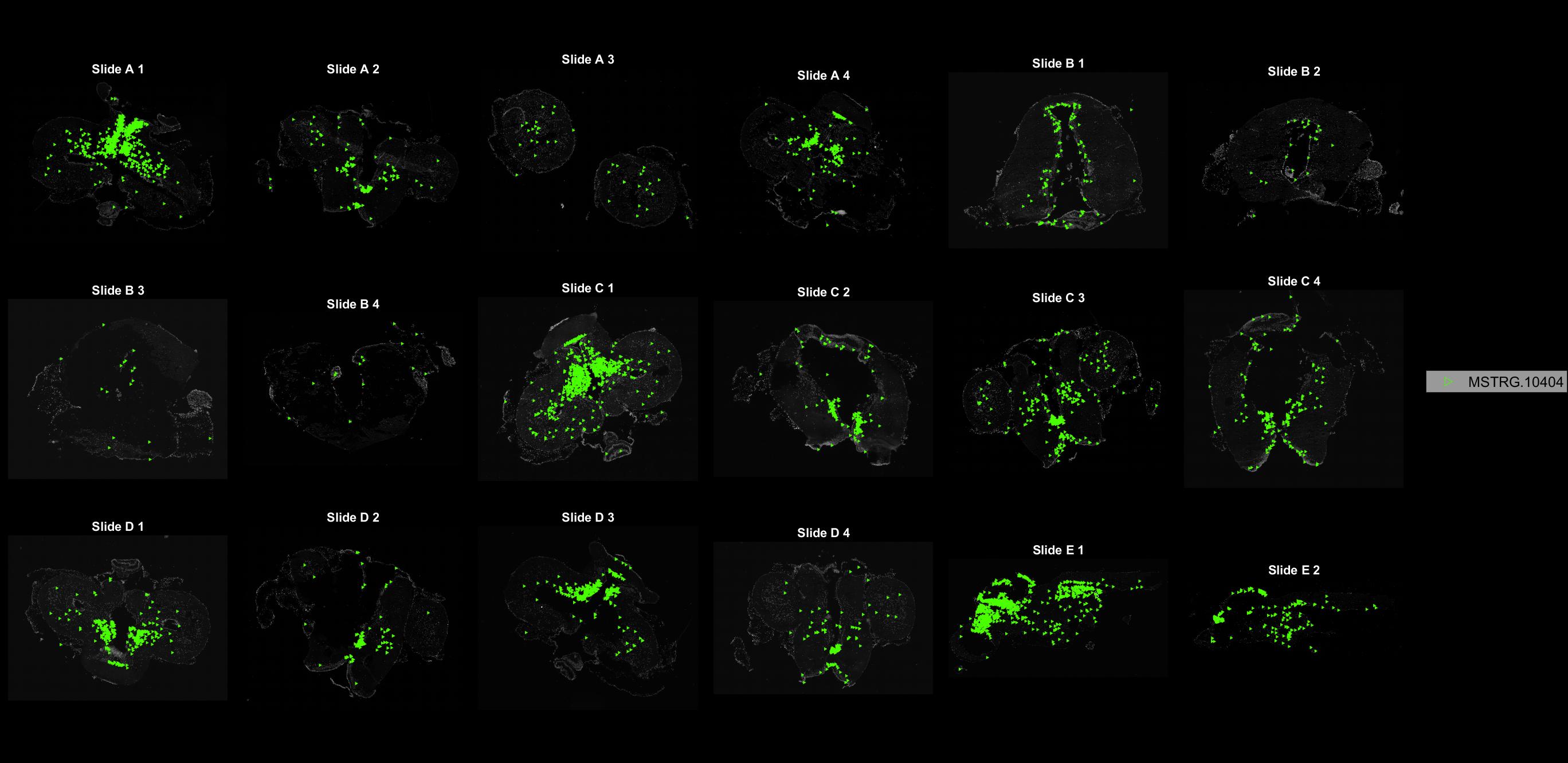

Supplement: Supplementary file 6 — In situ images produced in this study. [file 41559_2023_2170_MOESM6_ESM.zip › ISS/MSTRG.10404.jpg]

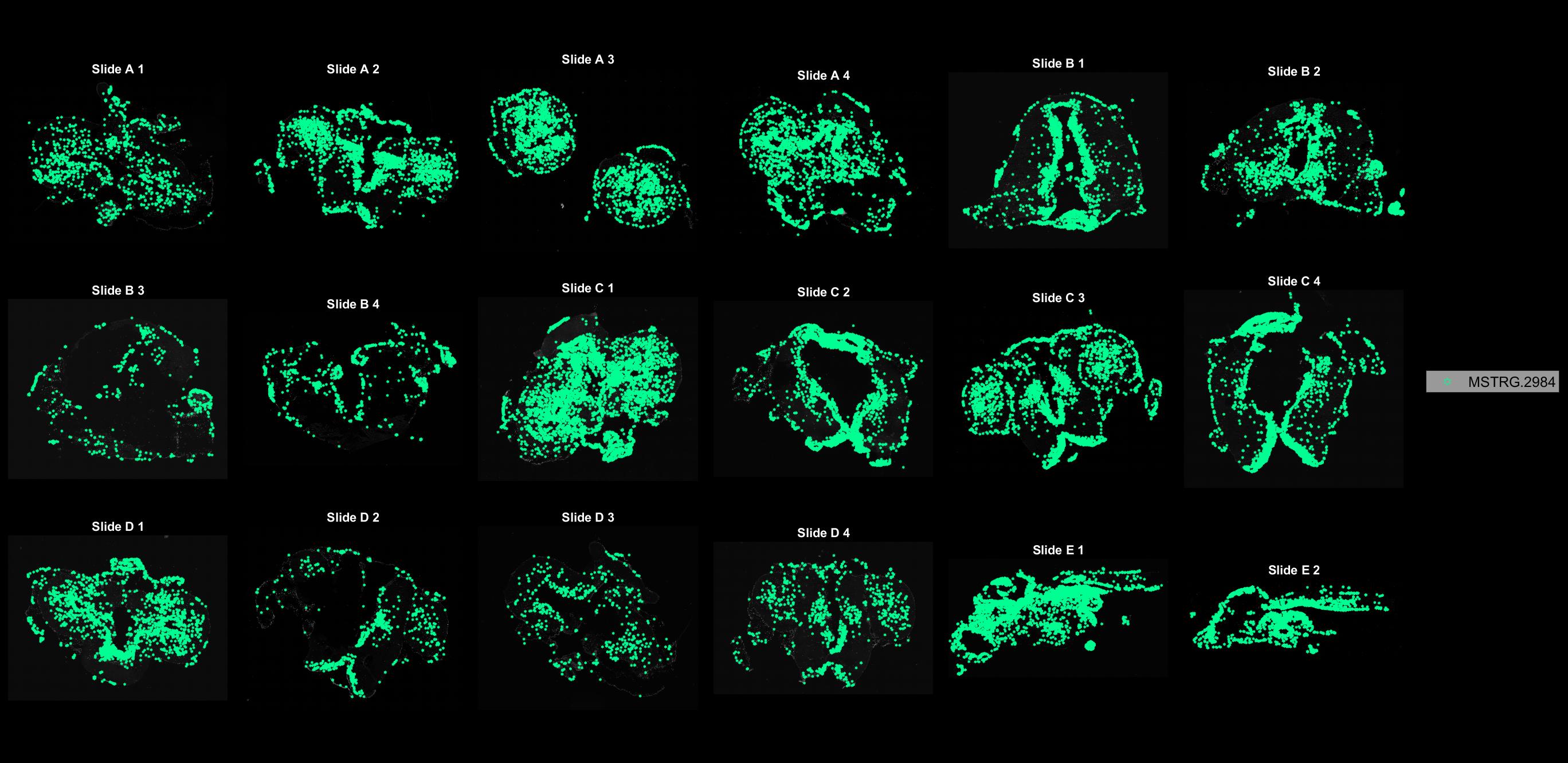

Supplement: Supplementary file 6 — In situ images produced in this study. [file 41559_2023_2170_MOESM6_ESM.zip › ISS/MSTRG.2984.jpg]

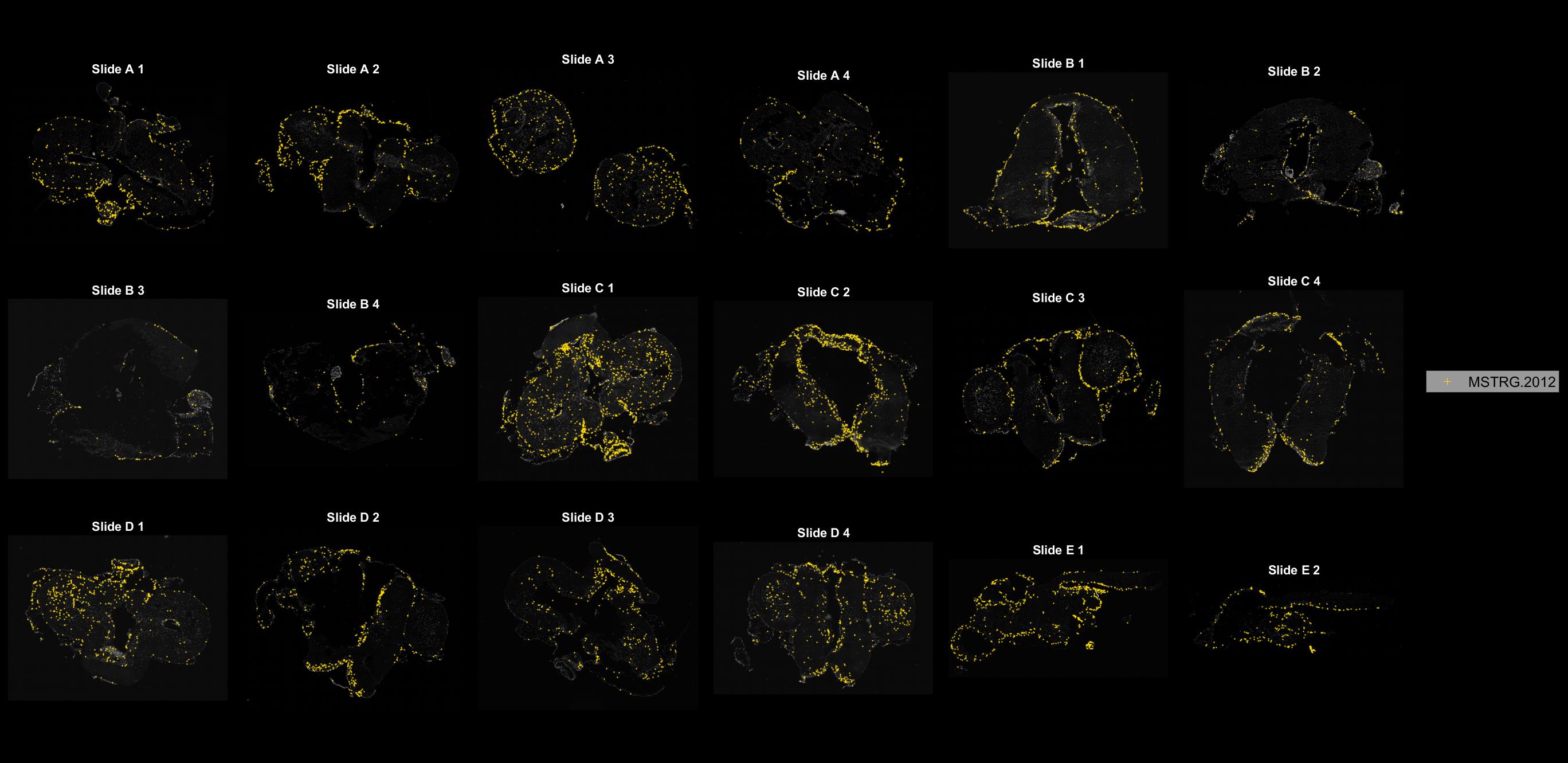

Supplement: Supplementary file 6 — In situ images produced in this study. [file 41559_2023_2170_MOESM6_ESM.zip › ISS/MSTRG.2012.jpg]

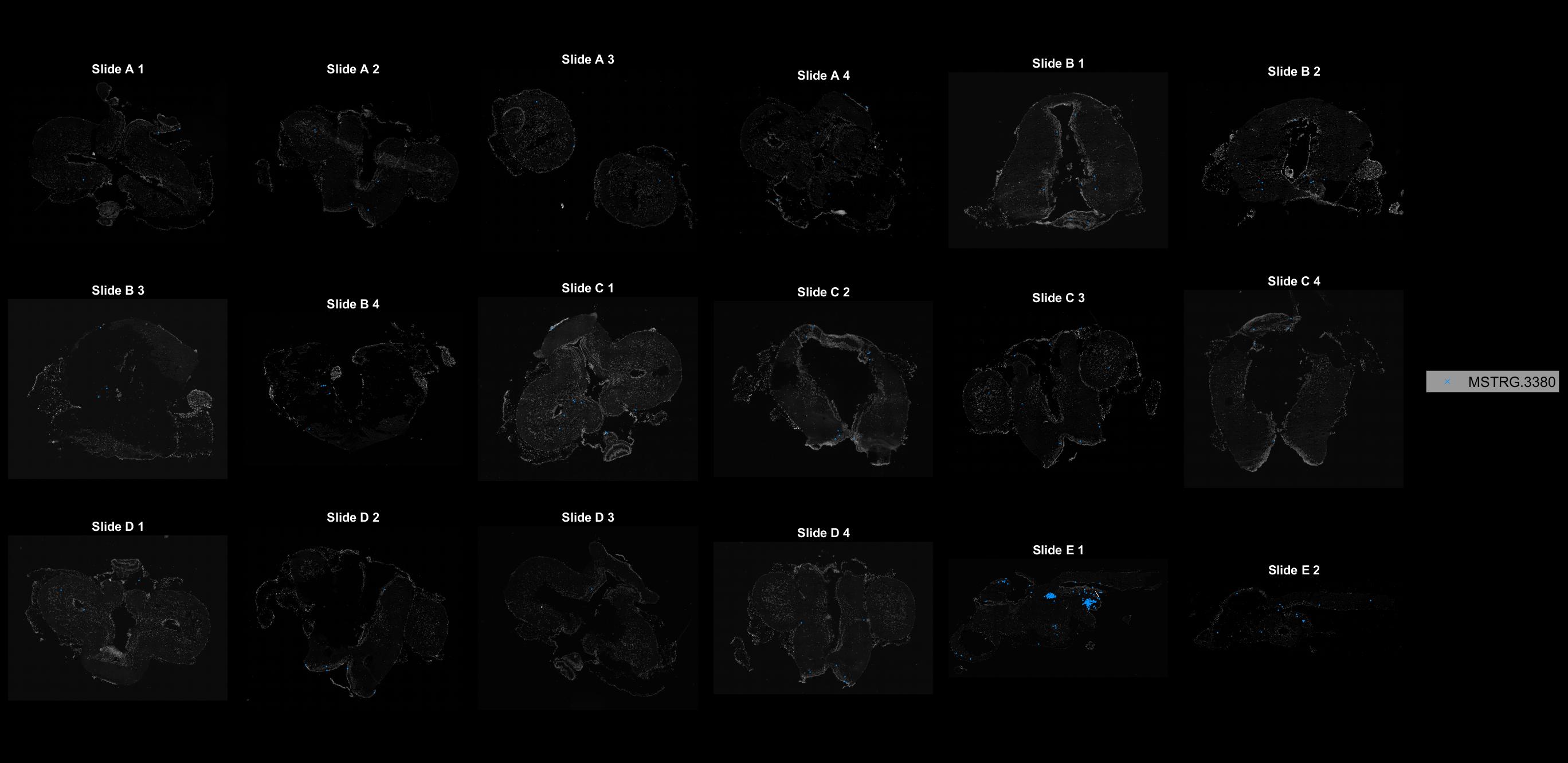

Supplement: Supplementary file 6 — In situ images produced in this study. [file 41559_2023_2170_MOESM6_ESM.zip › ISS/MSTRG.3380.jpg]

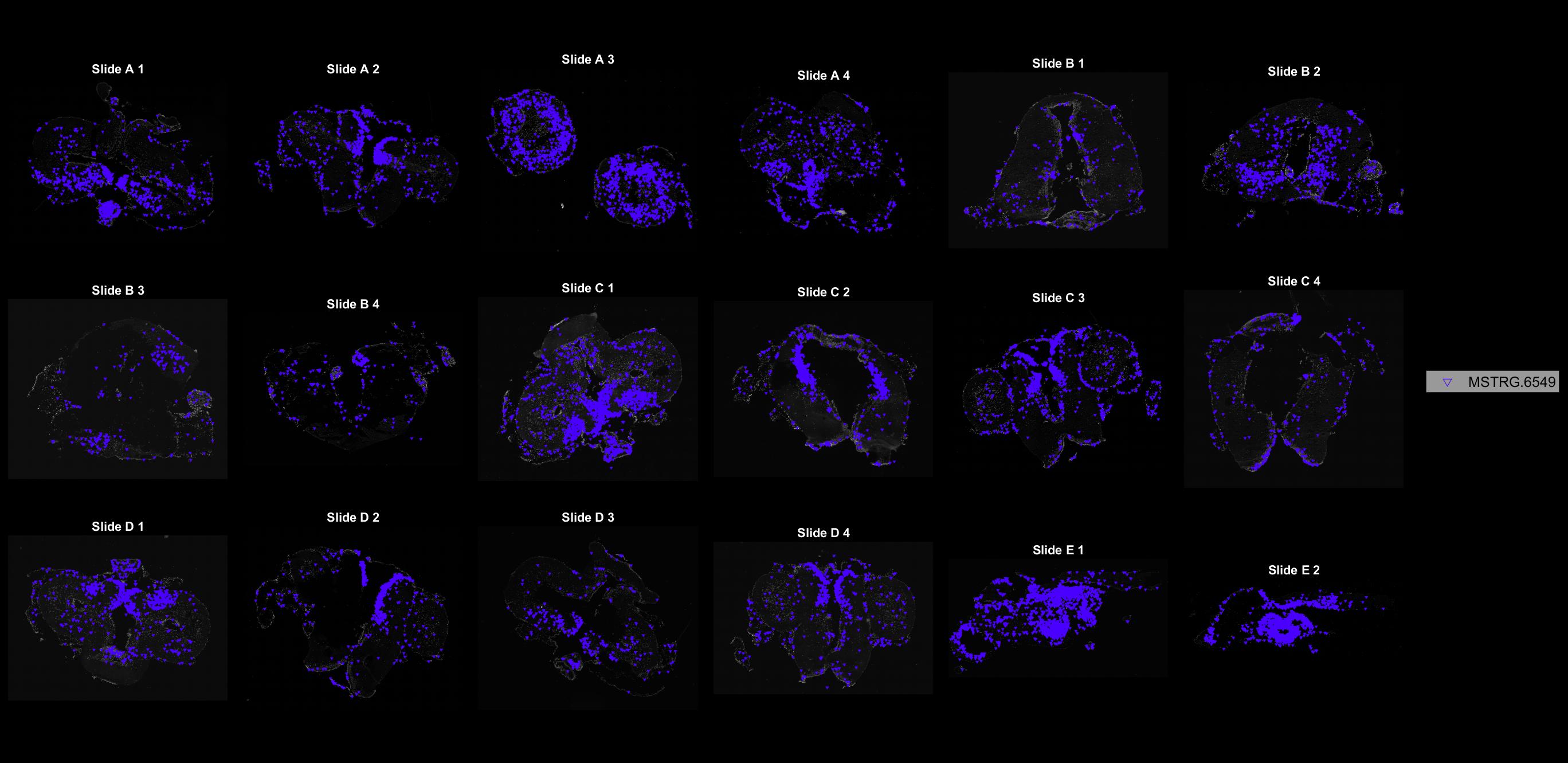

Supplement: Supplementary file 6 — In situ images produced in this study. [file 41559_2023_2170_MOESM6_ESM.zip › ISS/MSTRG.6549.jpg]

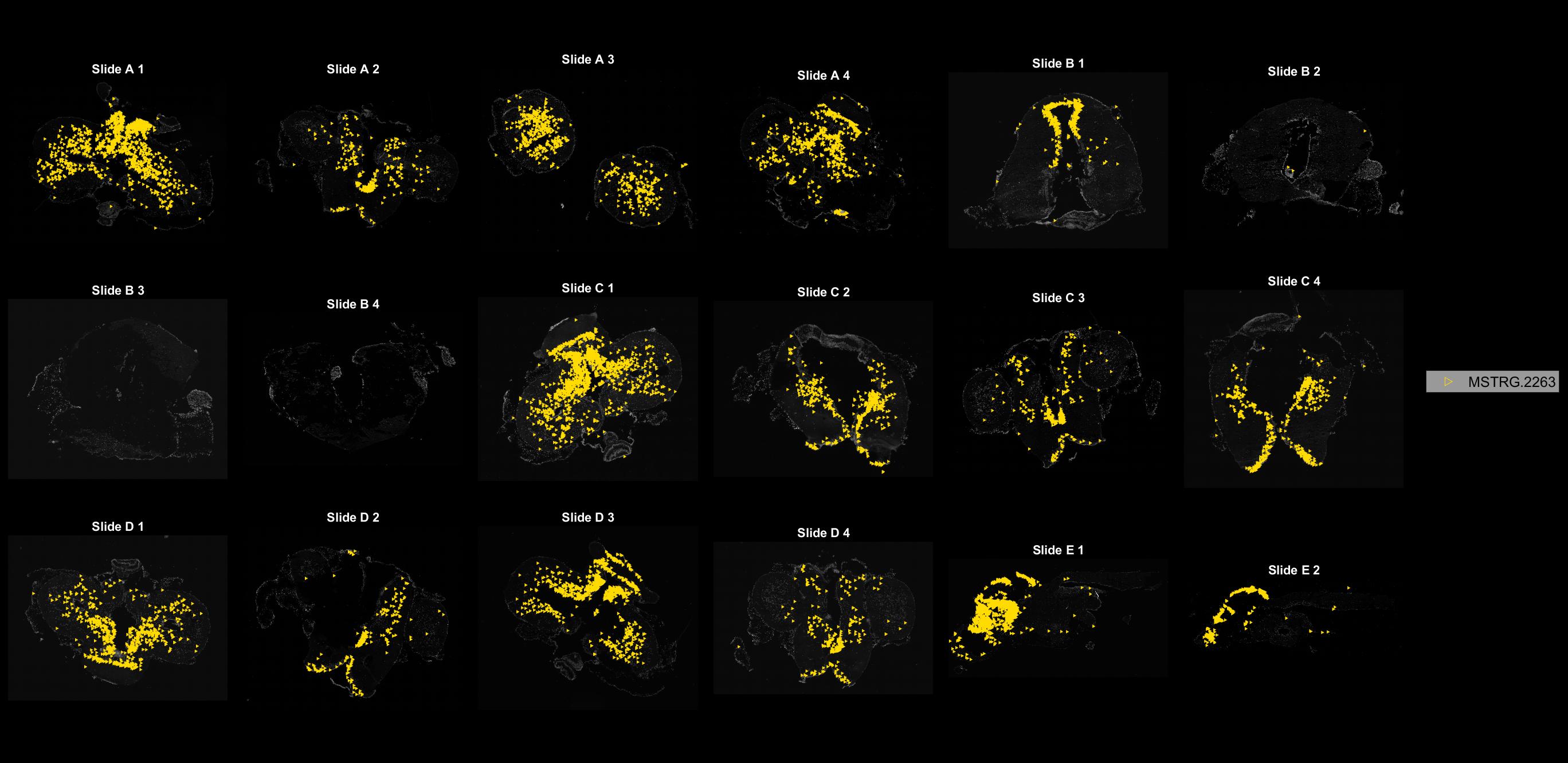

Supplement: Supplementary file 6 — In situ images produced in this study. [file 41559_2023_2170_MOESM6_ESM.zip › ISS/MSTRG.2263.jpg]

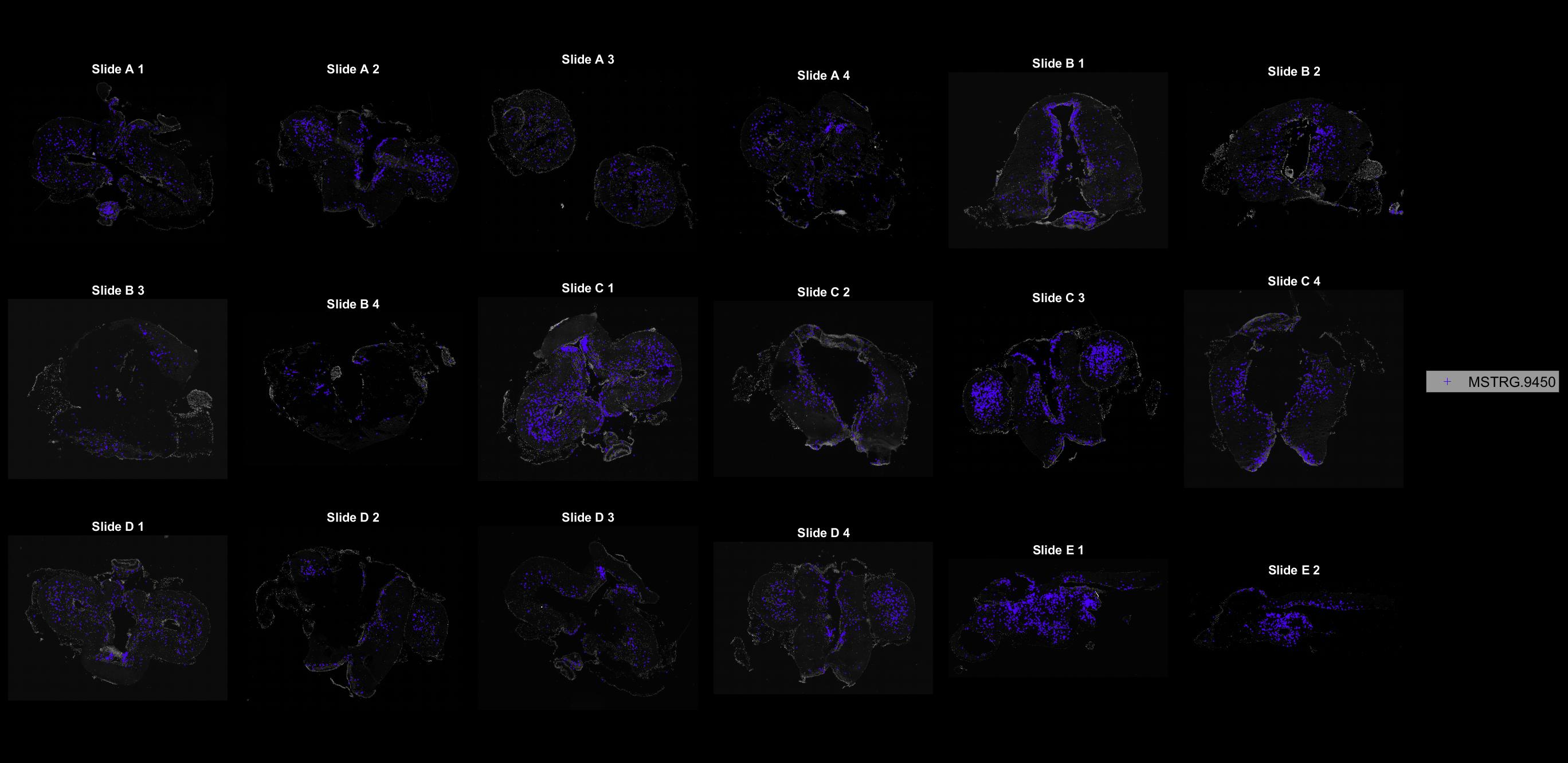

Supplement: Supplementary file 6 — In situ images produced in this study. [file 41559_2023_2170_MOESM6_ESM.zip › ISS/MSTRG.9450.jpg]

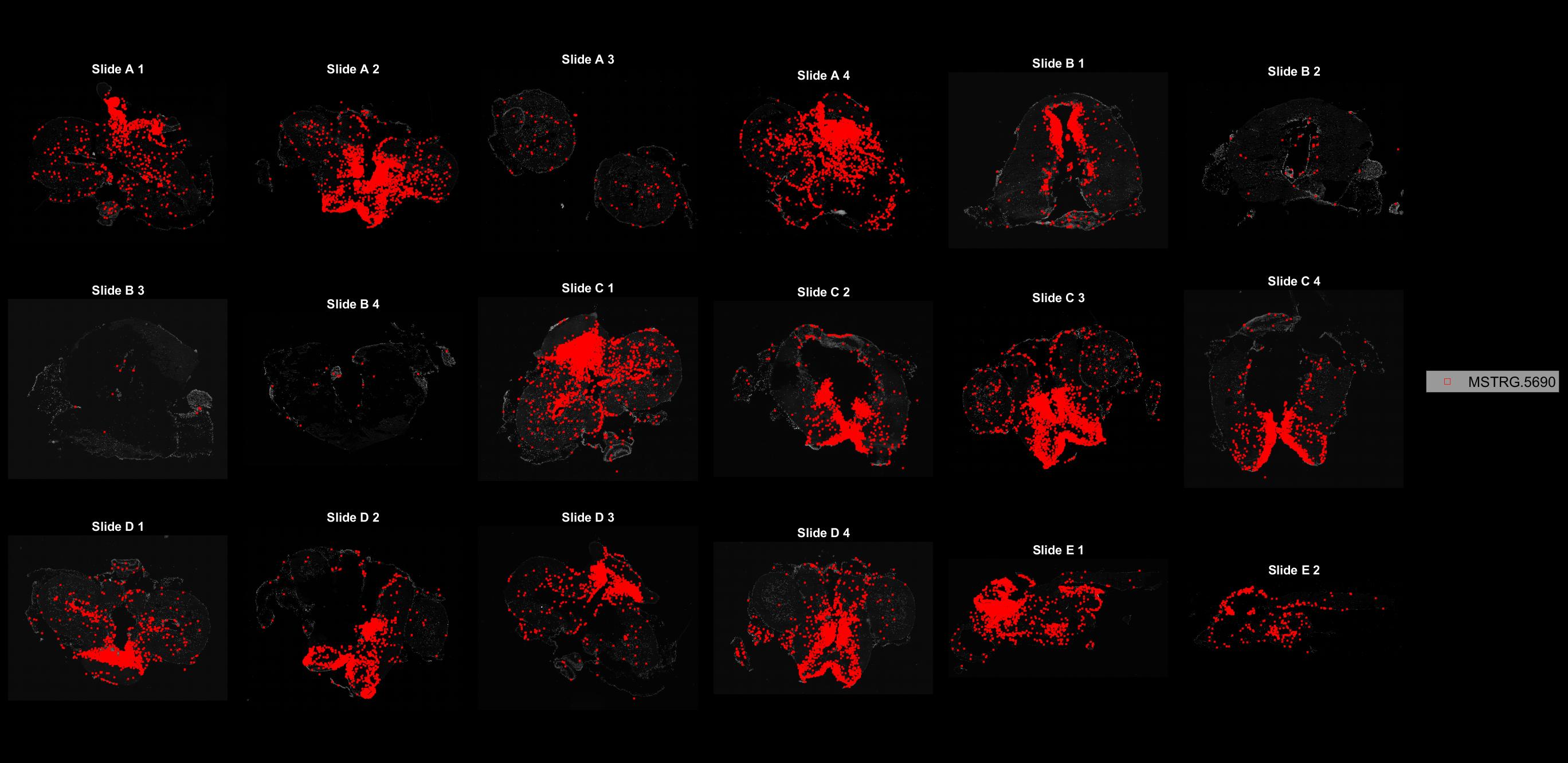

Supplement: Supplementary file 6 — In situ images produced in this study. [file 41559_2023_2170_MOESM6_ESM.zip › ISS/MSTRG.5690.jpg]

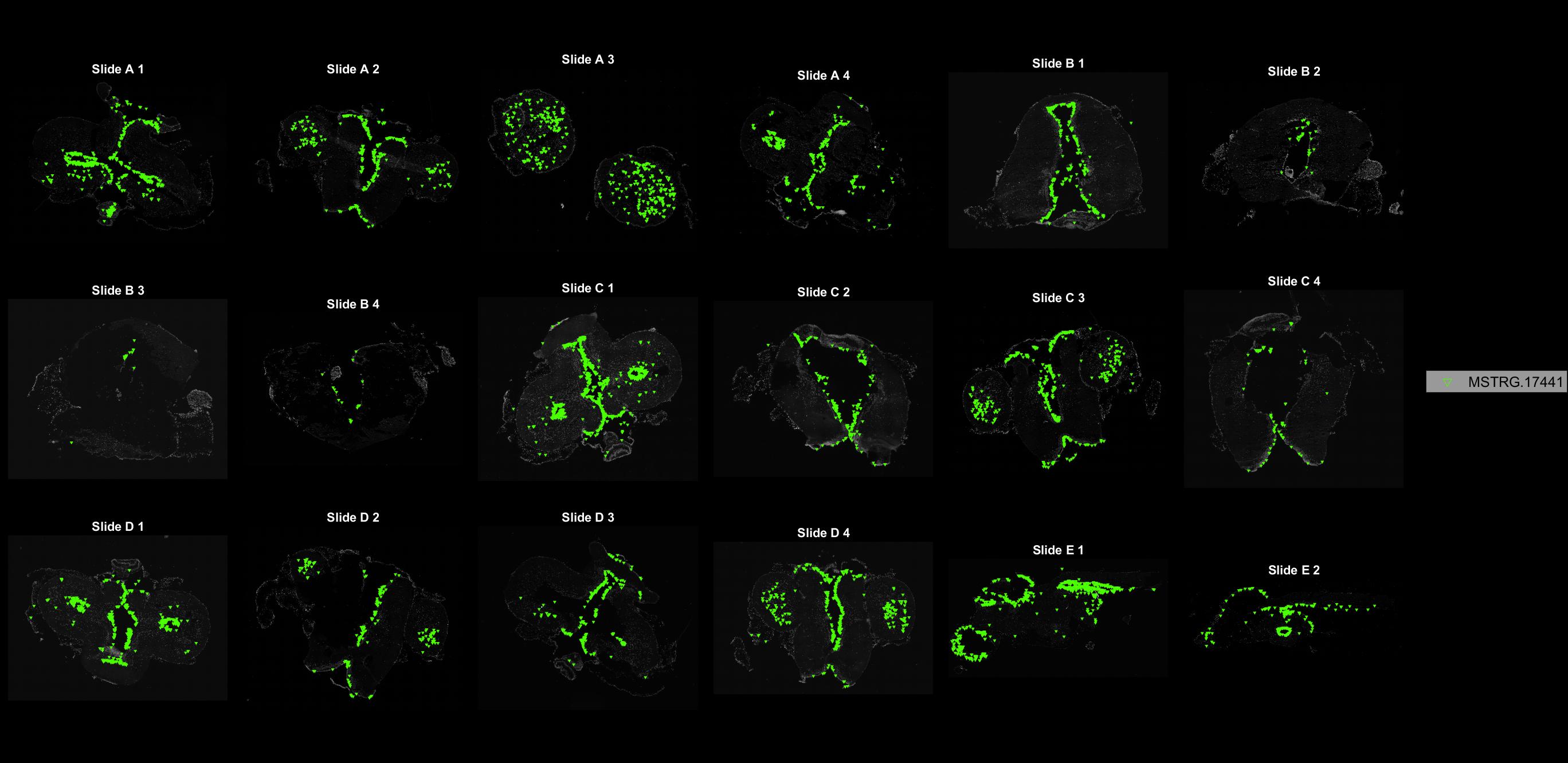

Supplement: Supplementary file 6 — In situ images produced in this study. [file 41559_2023_2170_MOESM6_ESM.zip › ISS/MSTRG.17441.jpg]

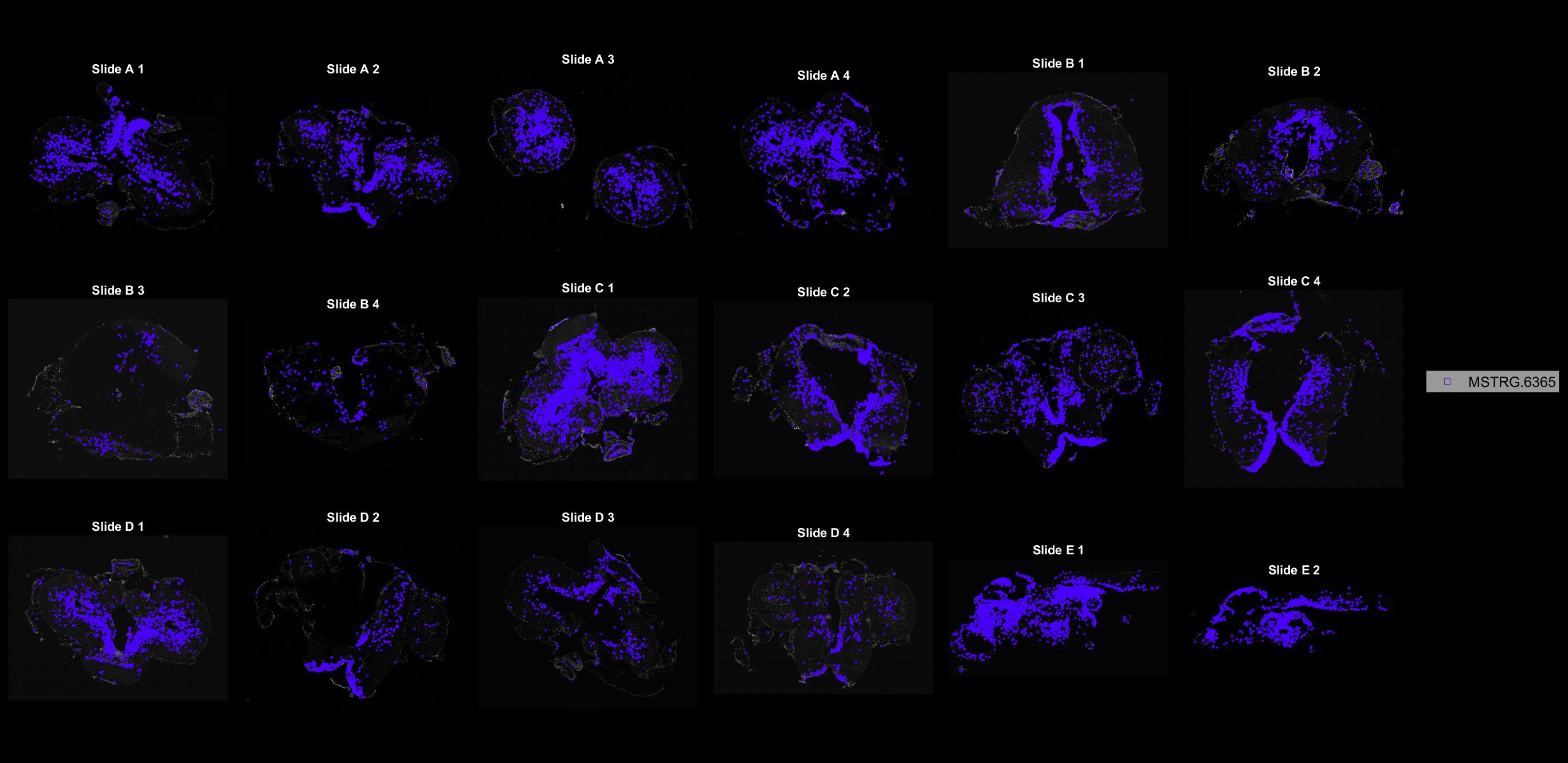

Supplement: Supplementary file 6 — In situ images produced in this study. [file 41559_2023_2170_MOESM6_ESM.zip › ISS/MSTRG.6365.jpg]

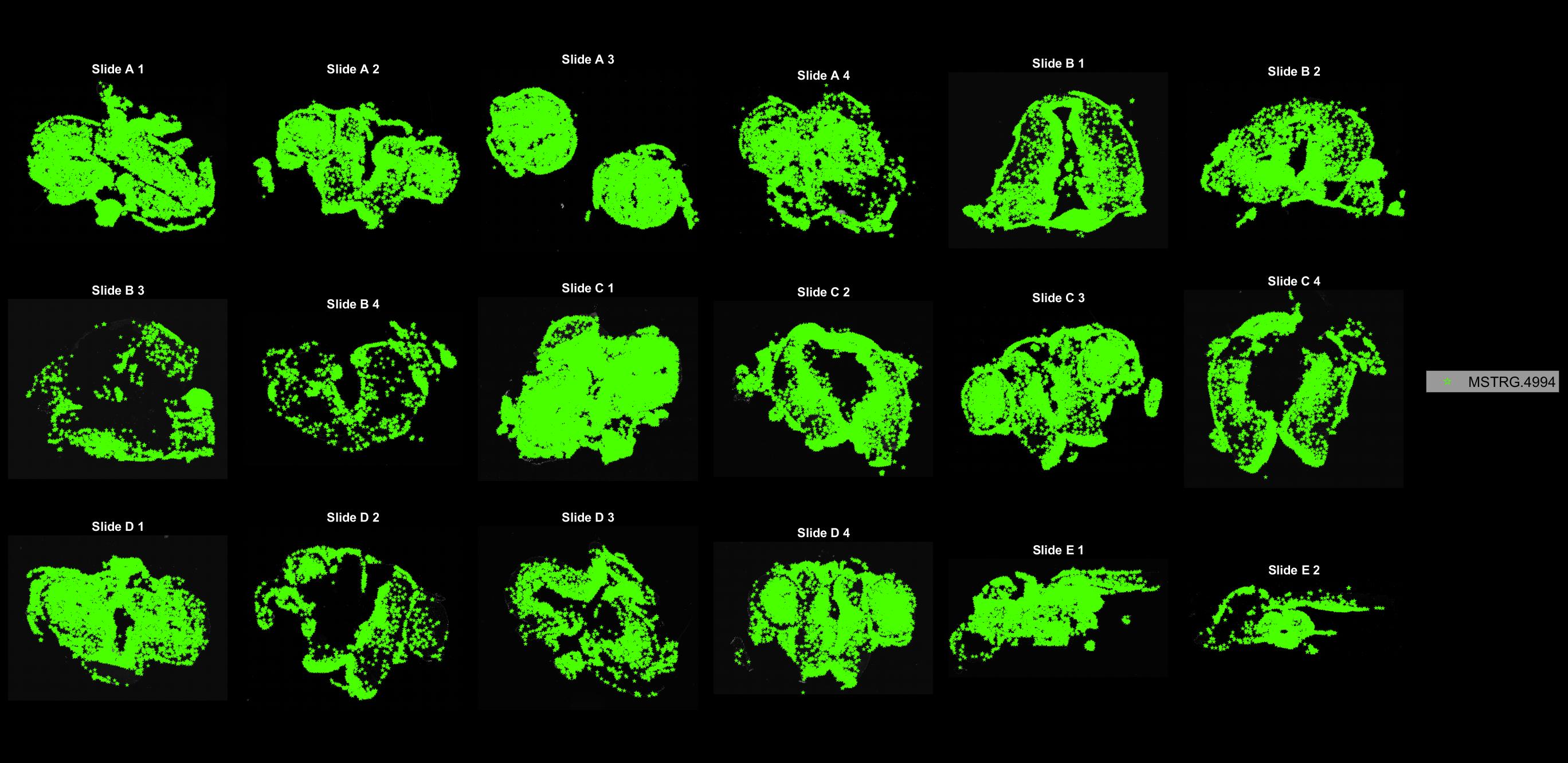

Supplement: Supplementary file 6 — In situ images produced in this study. [file 41559_2023_2170_MOESM6_ESM.zip › ISS/MSTRG.4994.jpg]

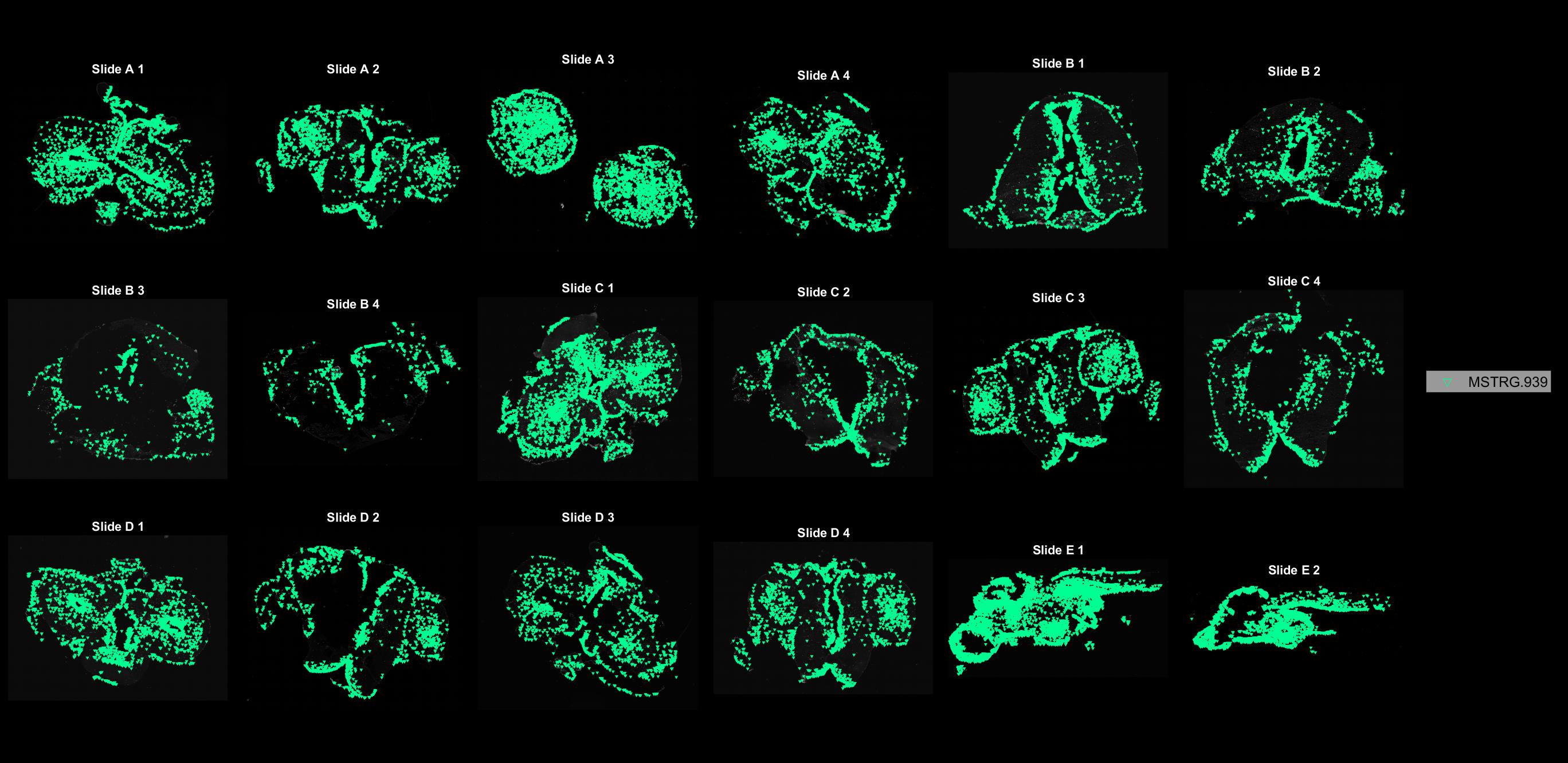

Supplement: Supplementary file 6 — In situ images produced in this study. [file 41559_2023_2170_MOESM6_ESM.zip › ISS/MSTRG.939.jpg]

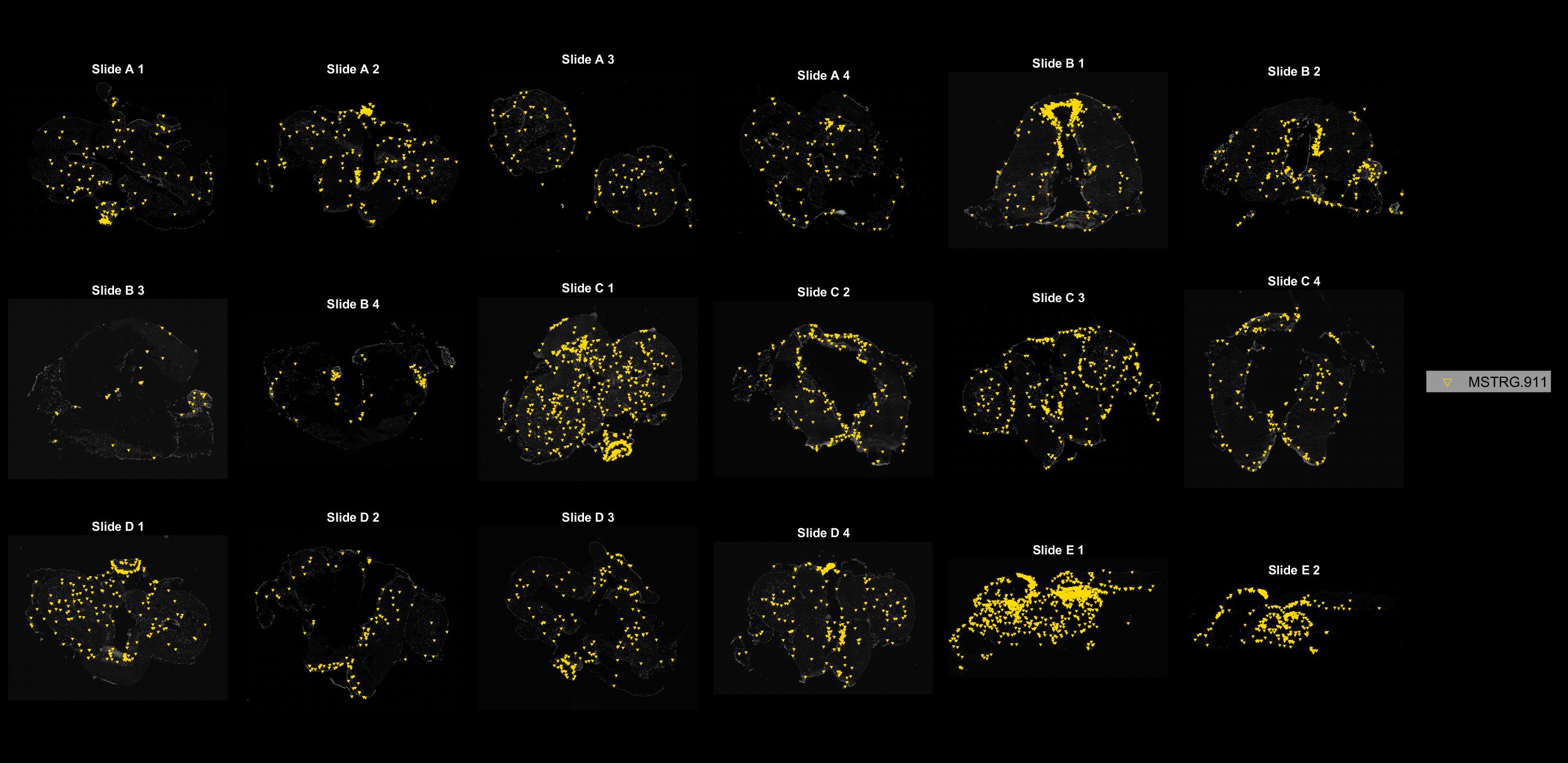

Supplement: Supplementary file 6 — In situ images produced in this study. [file 41559_2023_2170_MOESM6_ESM.zip › ISS/MSTRG.911.jpg]

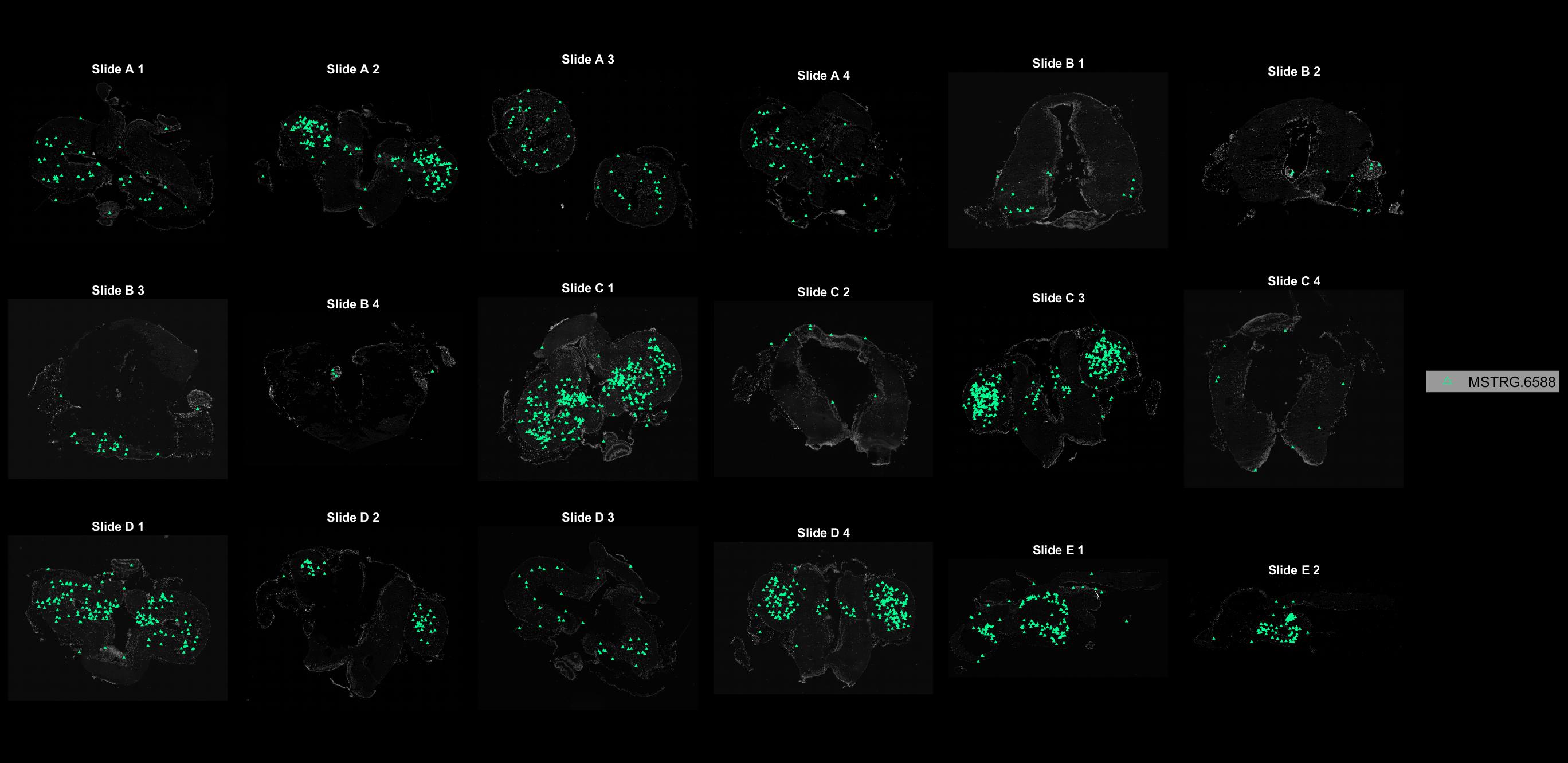

Supplement: Supplementary file 6 — In situ images produced in this study. [file 41559_2023_2170_MOESM6_ESM.zip › ISS/MSTRG.6588.jpg]

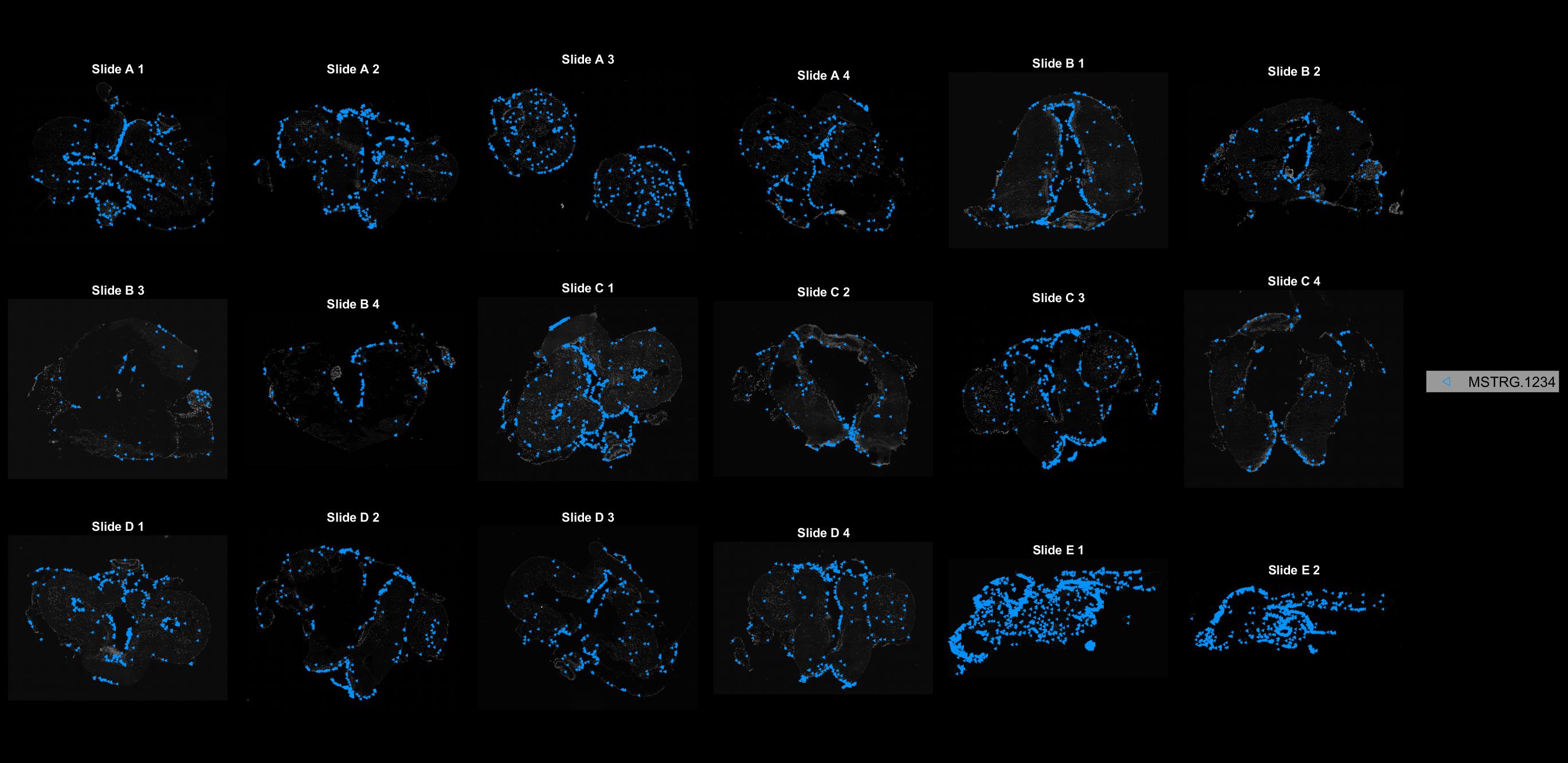

Supplement: Supplementary file 6 — In situ images produced in this study. [file 41559_2023_2170_MOESM6_ESM.zip › ISS/MSTRG.1234.jpg]

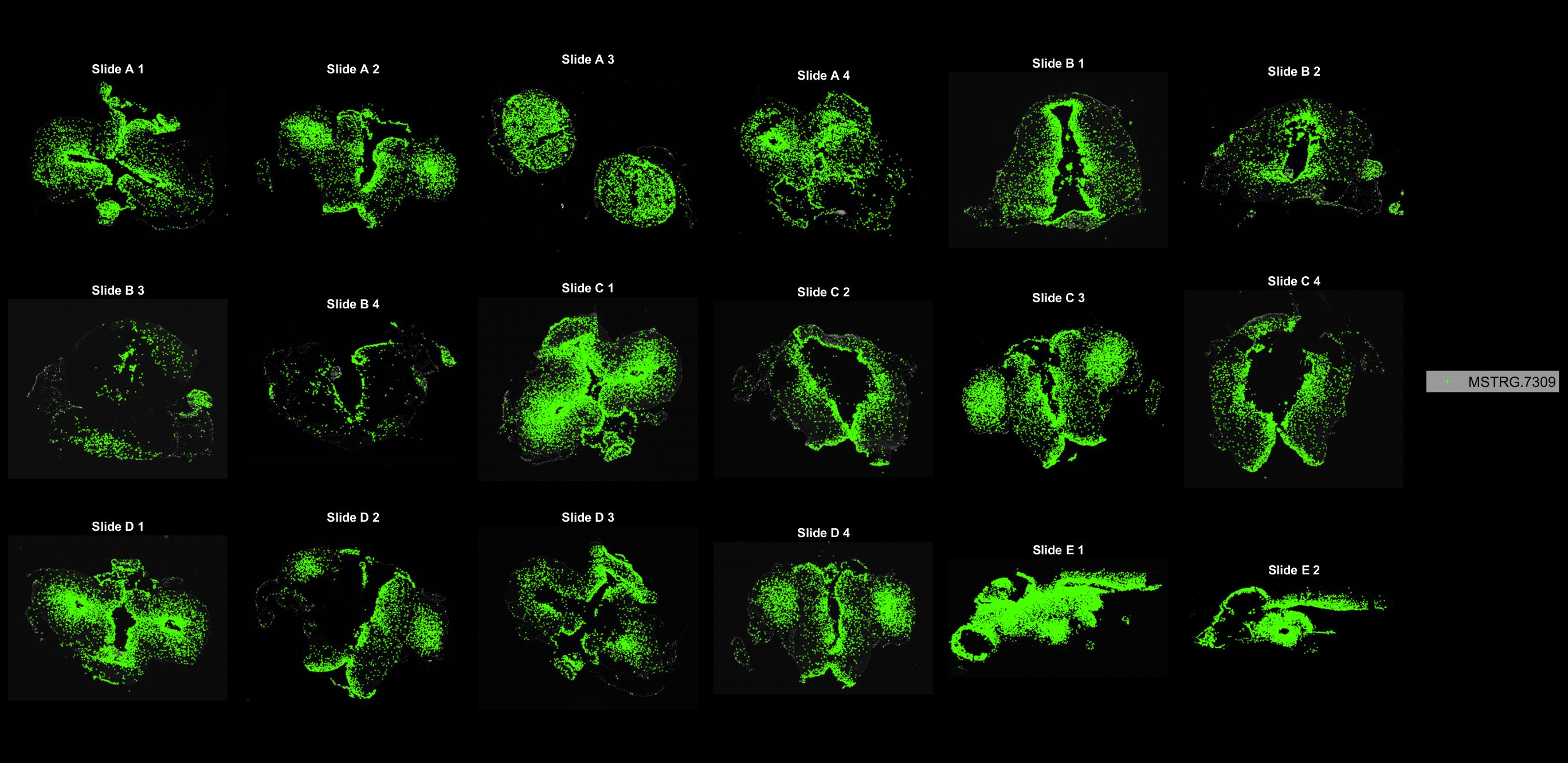

Supplement: Supplementary file 6 — In situ images produced in this study. [file 41559_2023_2170_MOESM6_ESM.zip › ISS/MSTRG.7309.jpg]

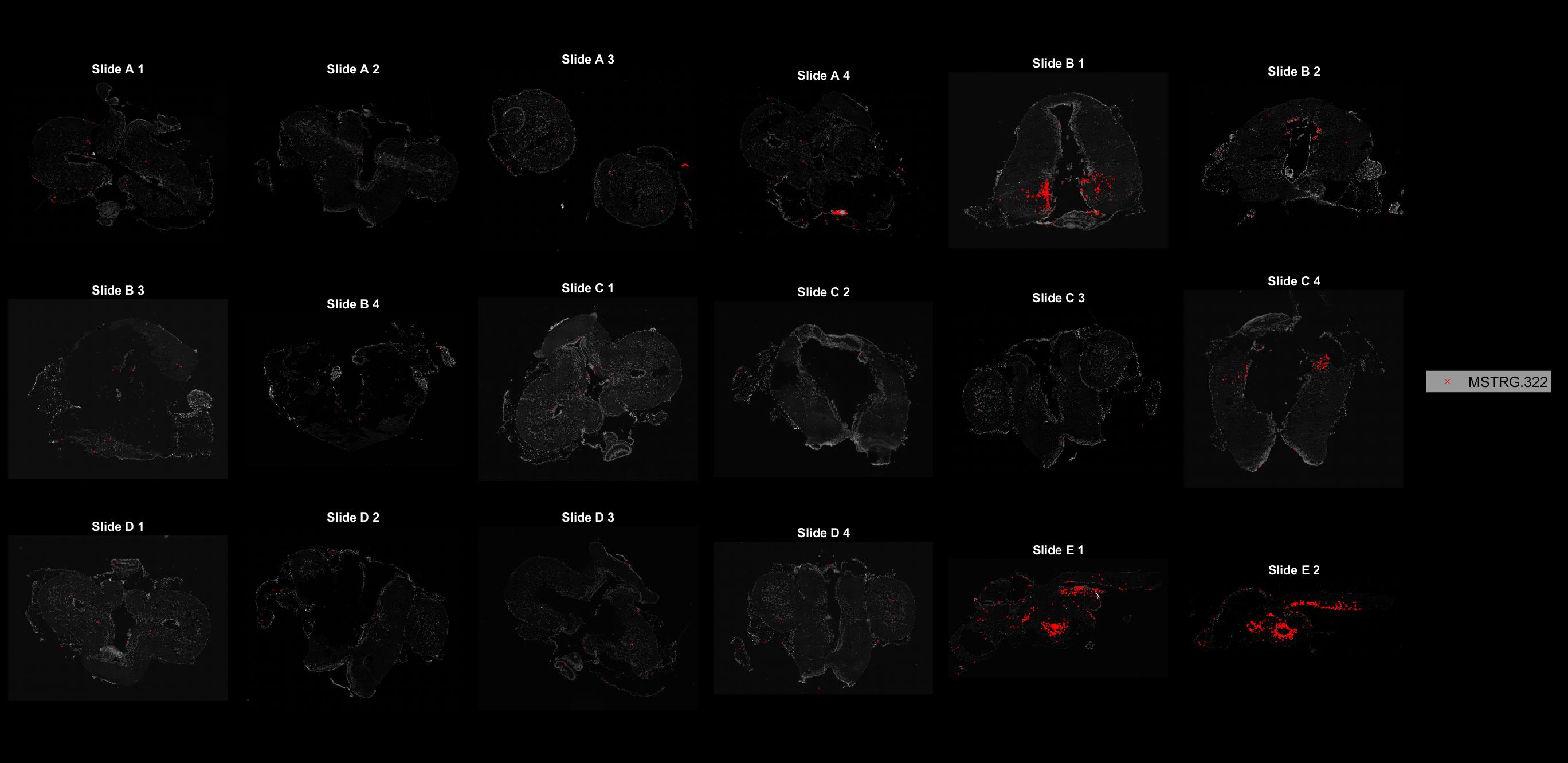

Supplement: Supplementary file 6 — In situ images produced in this study. [file 41559_2023_2170_MOESM6_ESM.zip › ISS/MSTRG.322.jpg]

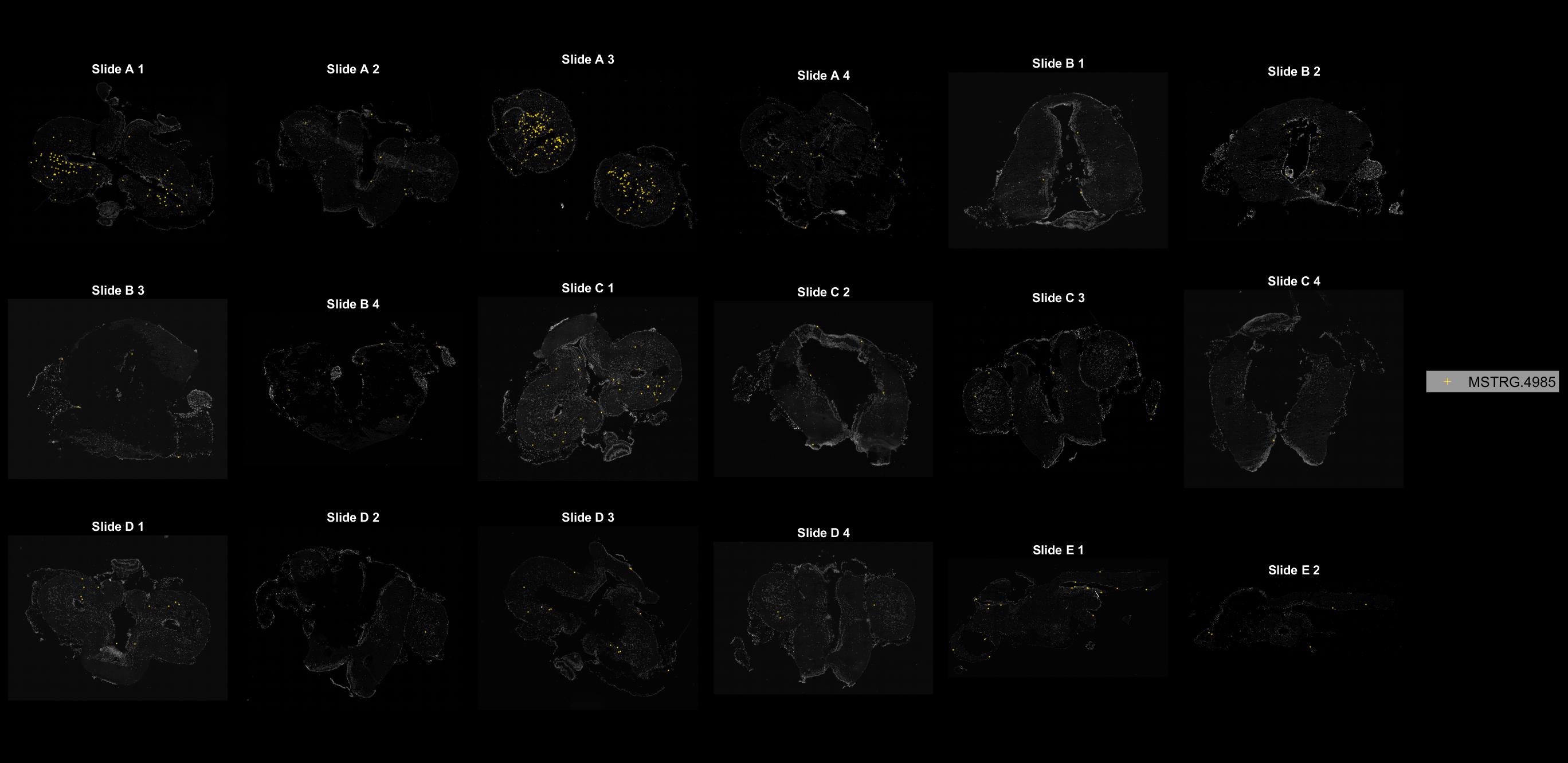

Supplement: Supplementary file 6 — In situ images produced in this study. [file 41559_2023_2170_MOESM6_ESM.zip › ISS/MSTRG.4985.jpg]

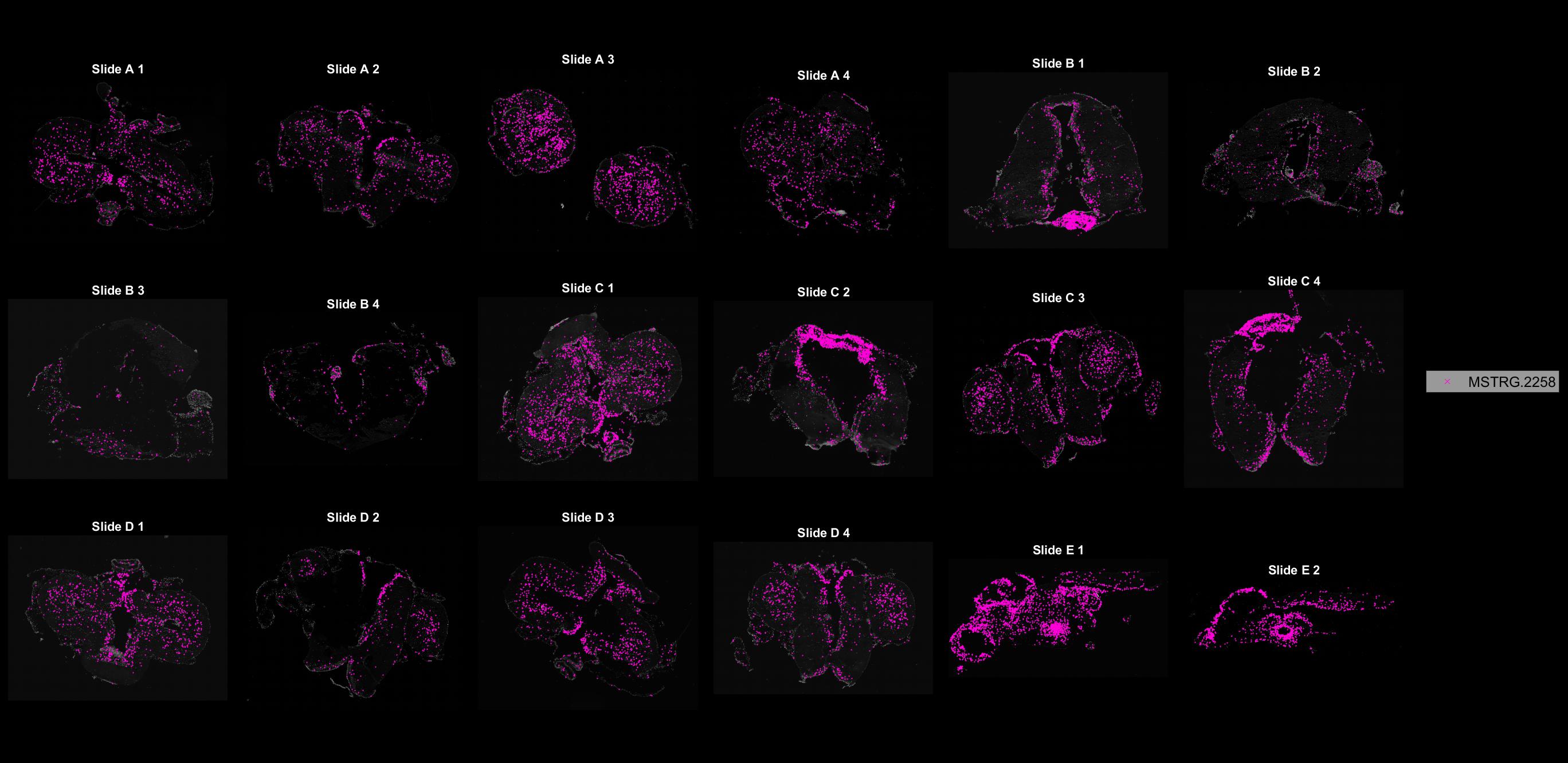

Supplement: Supplementary file 6 — In situ images produced in this study. [file 41559_2023_2170_MOESM6_ESM.zip › ISS/MSTRG.2258.jpg]

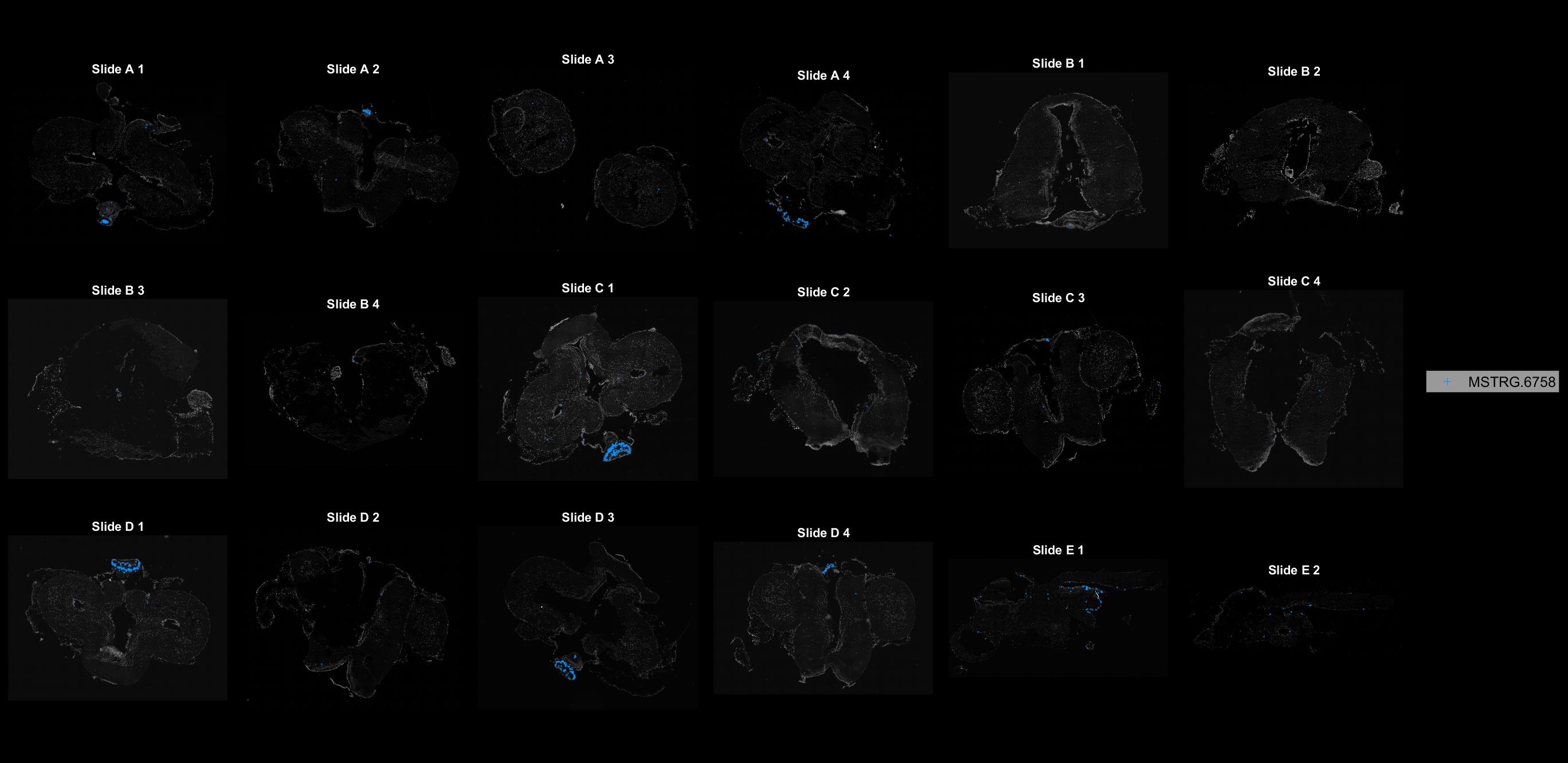

Supplement: Supplementary file 6 — In situ images produced in this study. [file 41559_2023_2170_MOESM6_ESM.zip › ISS/MSTRG.6758.jpg]

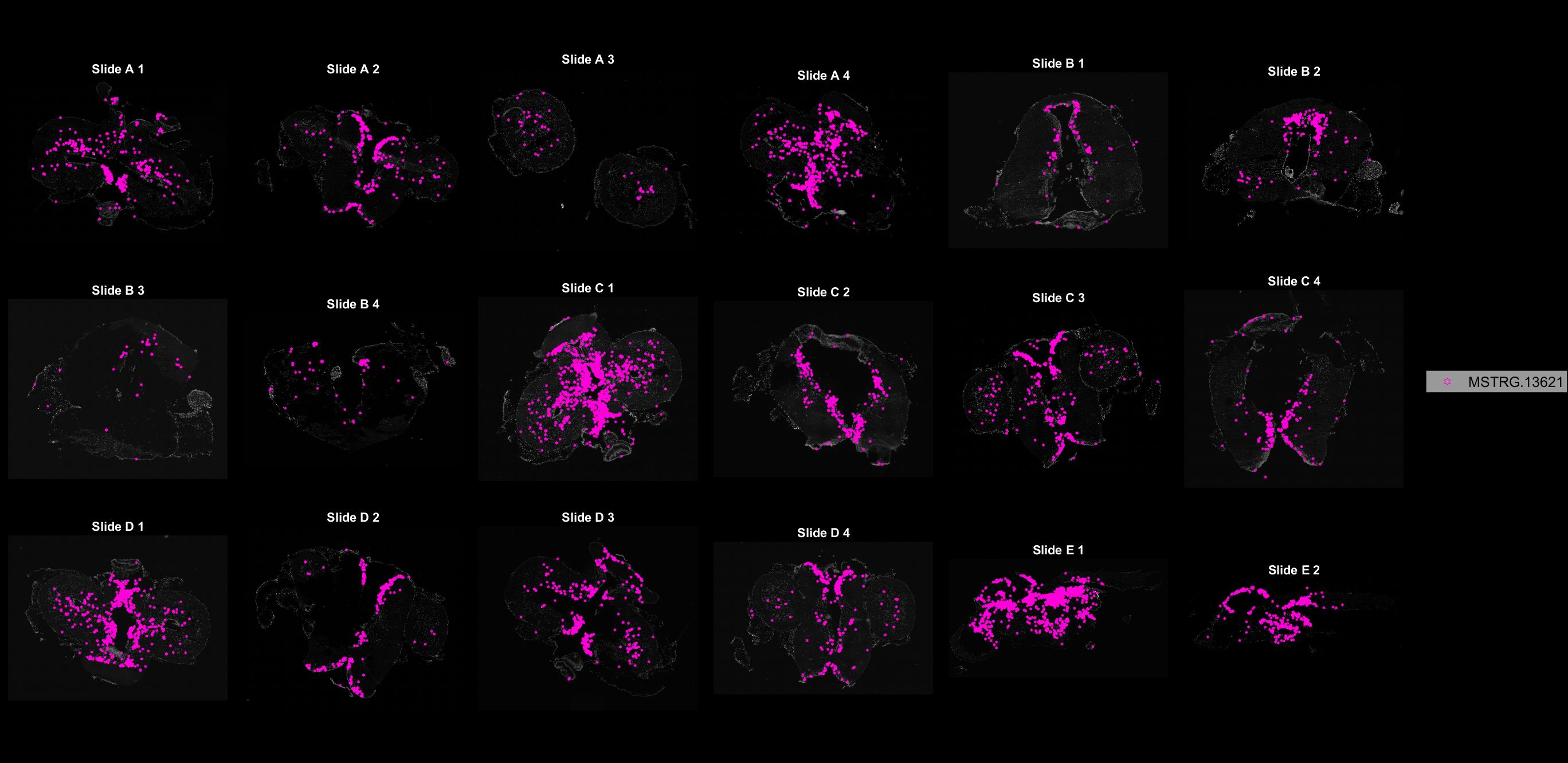

Supplement: Supplementary file 6 — In situ images produced in this study. [file 41559_2023_2170_MOESM6_ESM.zip › ISS/MSTRG.13621.jpg]

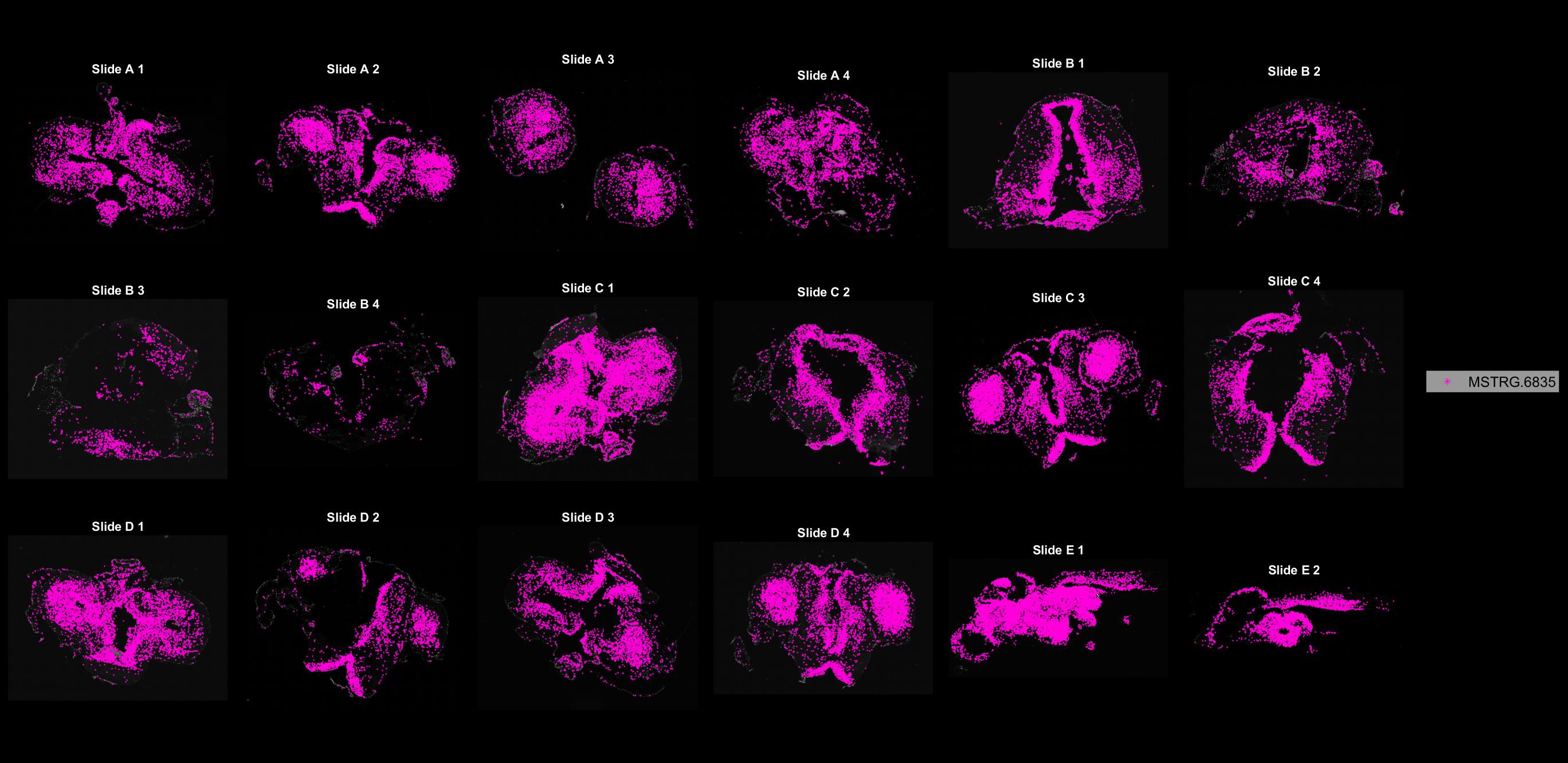

Supplement: Supplementary file 6 — In situ images produced in this study. [file 41559_2023_2170_MOESM6_ESM.zip › ISS/MSTRG.6835.jpg]

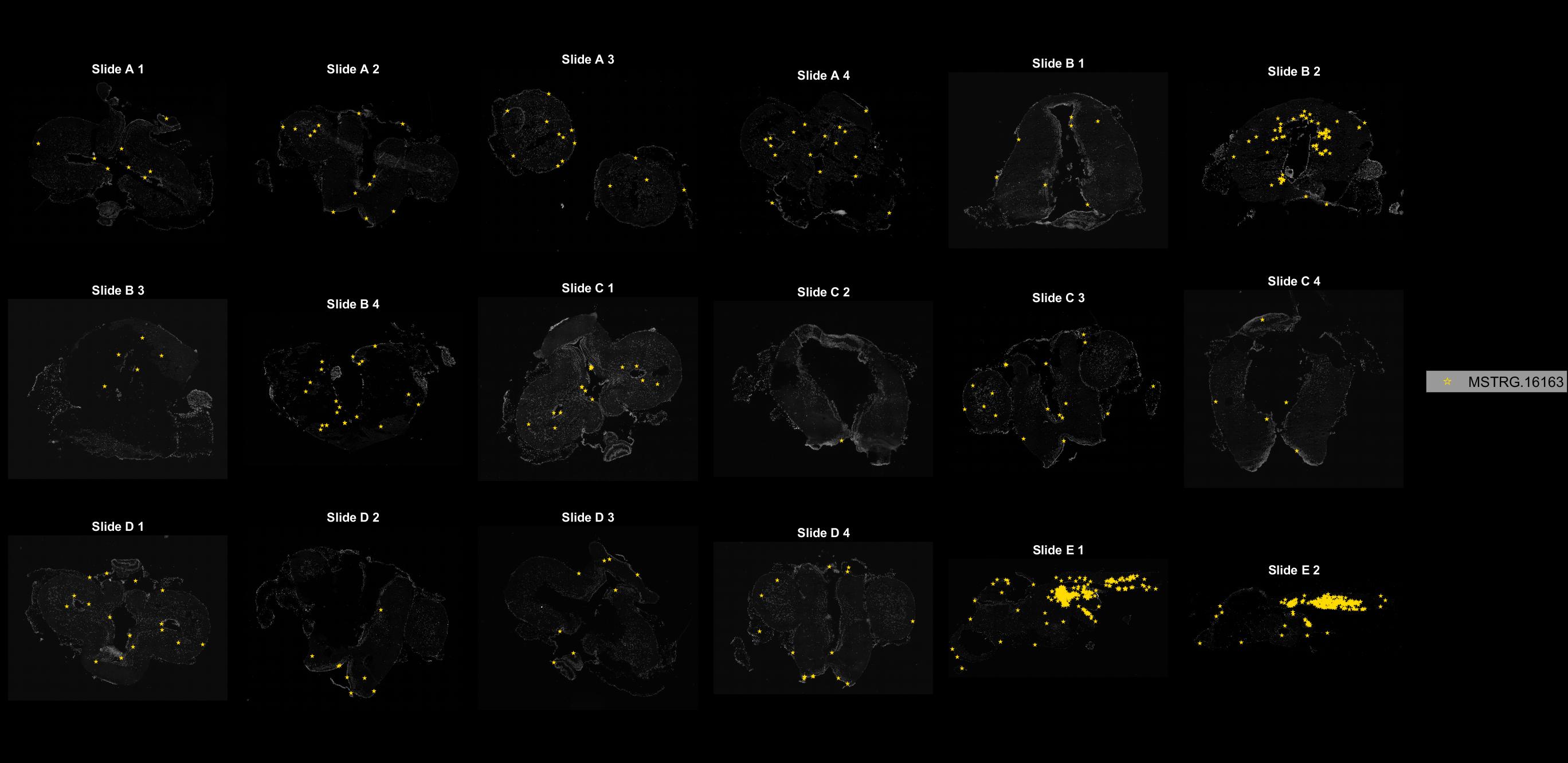

Supplement: Supplementary file 6 — In situ images produced in this study. [file 41559_2023_2170_MOESM6_ESM.zip › ISS/MSTRG.16163.jpg]

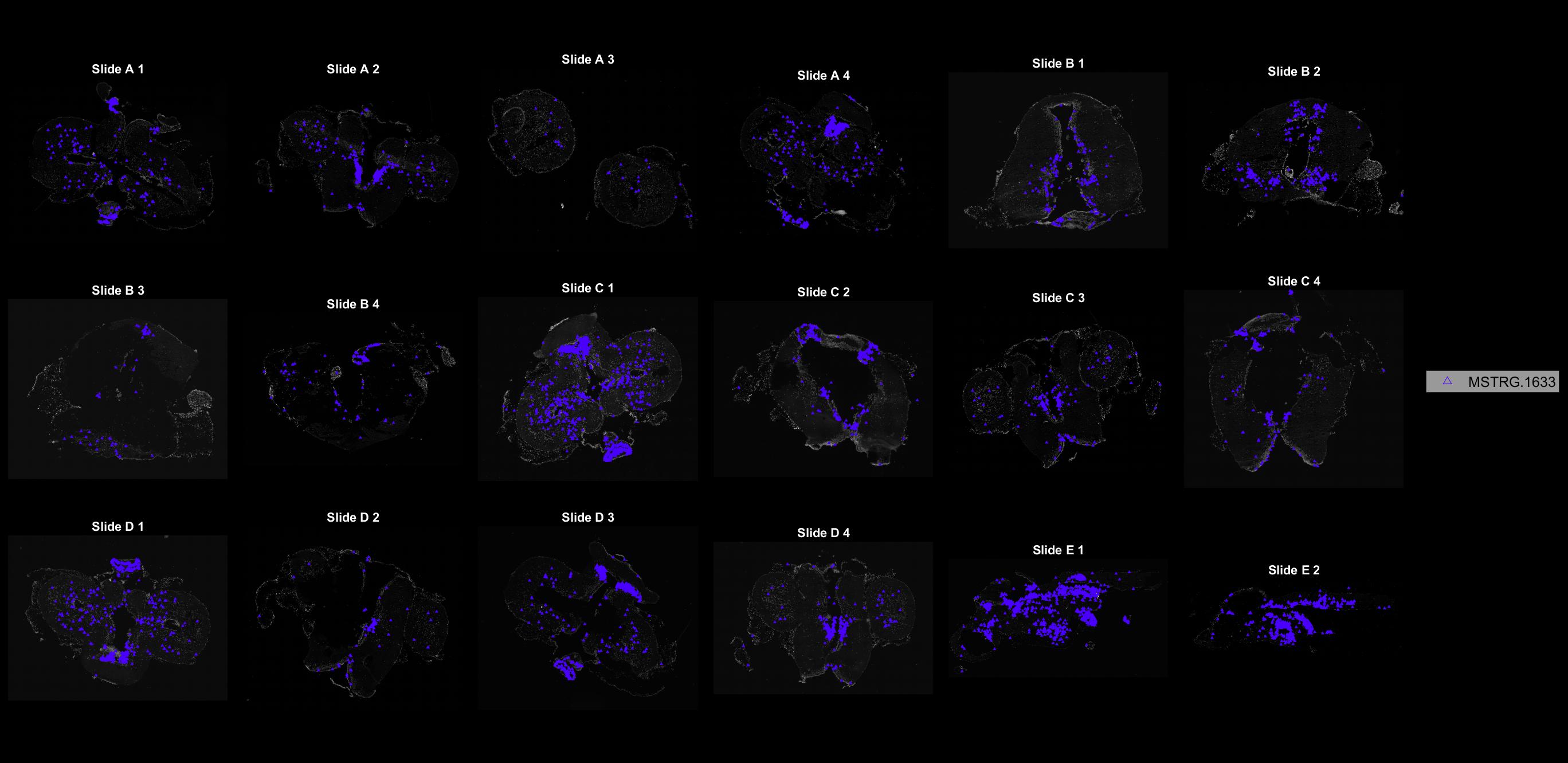

Supplement: Supplementary file 6 — In situ images produced in this study. [file 41559_2023_2170_MOESM6_ESM.zip › ISS/MSTRG.1633.jpg]

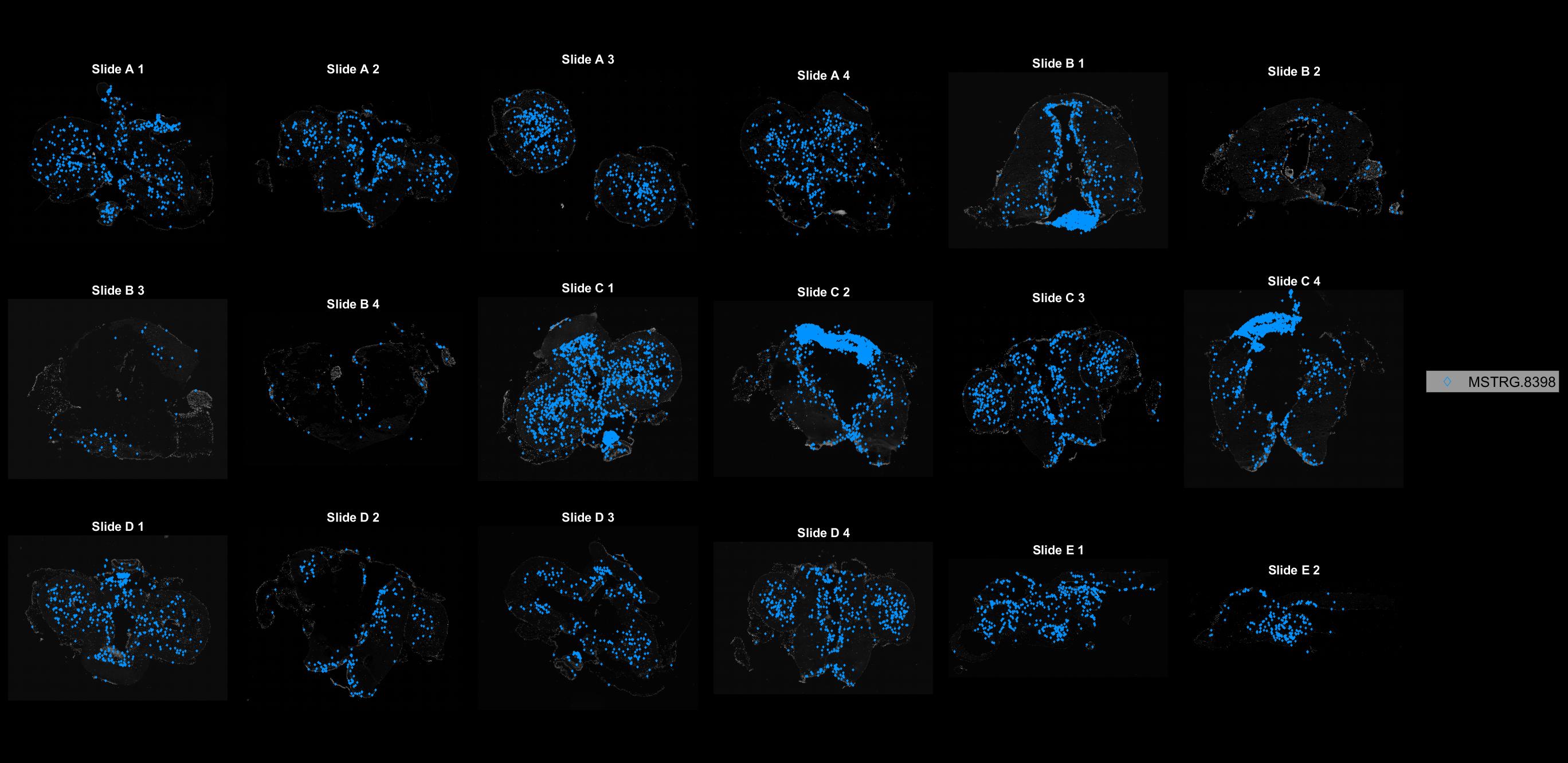

Supplement: Supplementary file 6 — In situ images produced in this study. [file 41559_2023_2170_MOESM6_ESM.zip › ISS/MSTRG.8398.jpg]

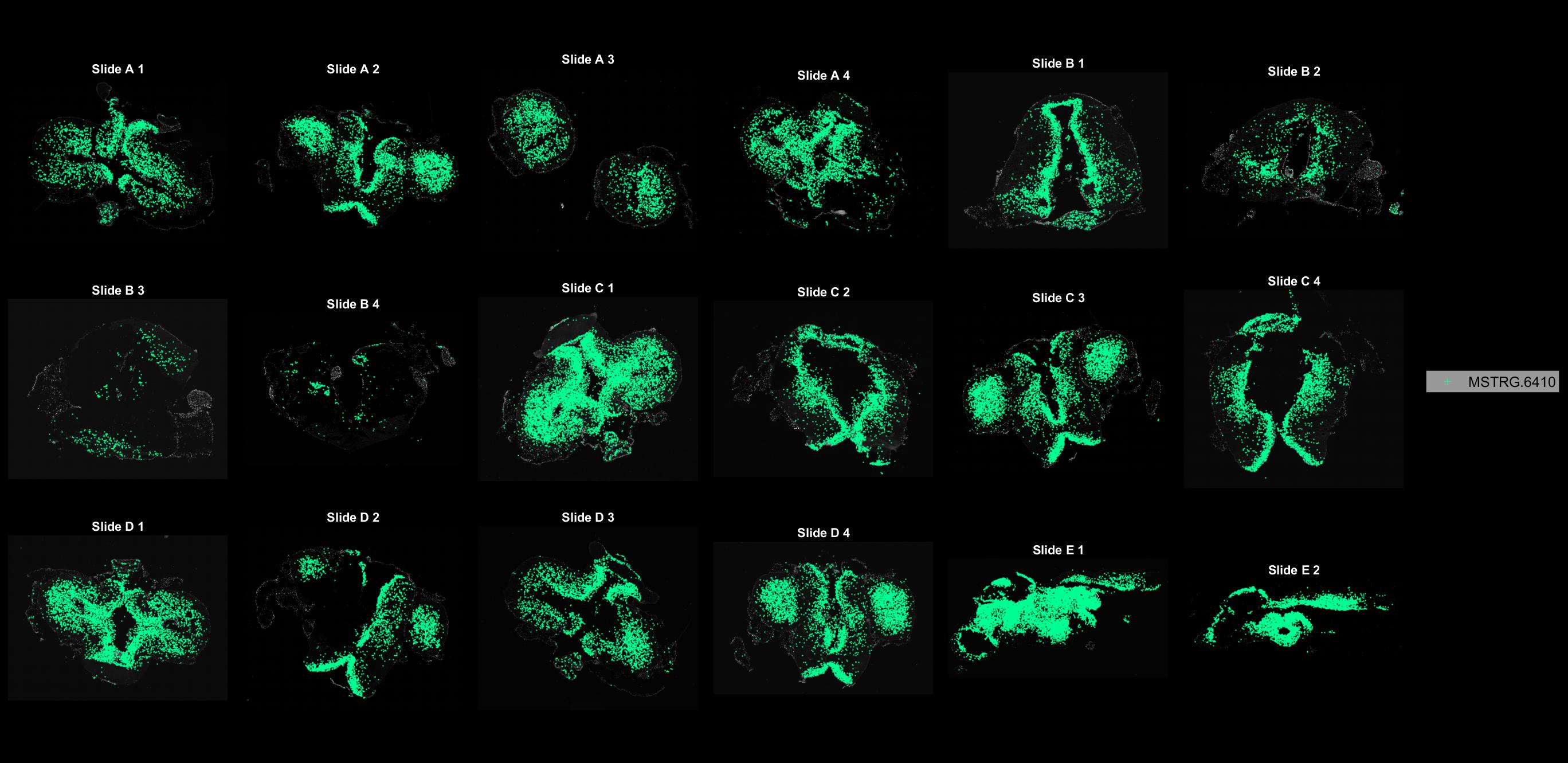

Supplement: Supplementary file 6 — In situ images produced in this study. [file 41559_2023_2170_MOESM6_ESM.zip › ISS/MSTRG.6410.jpg]

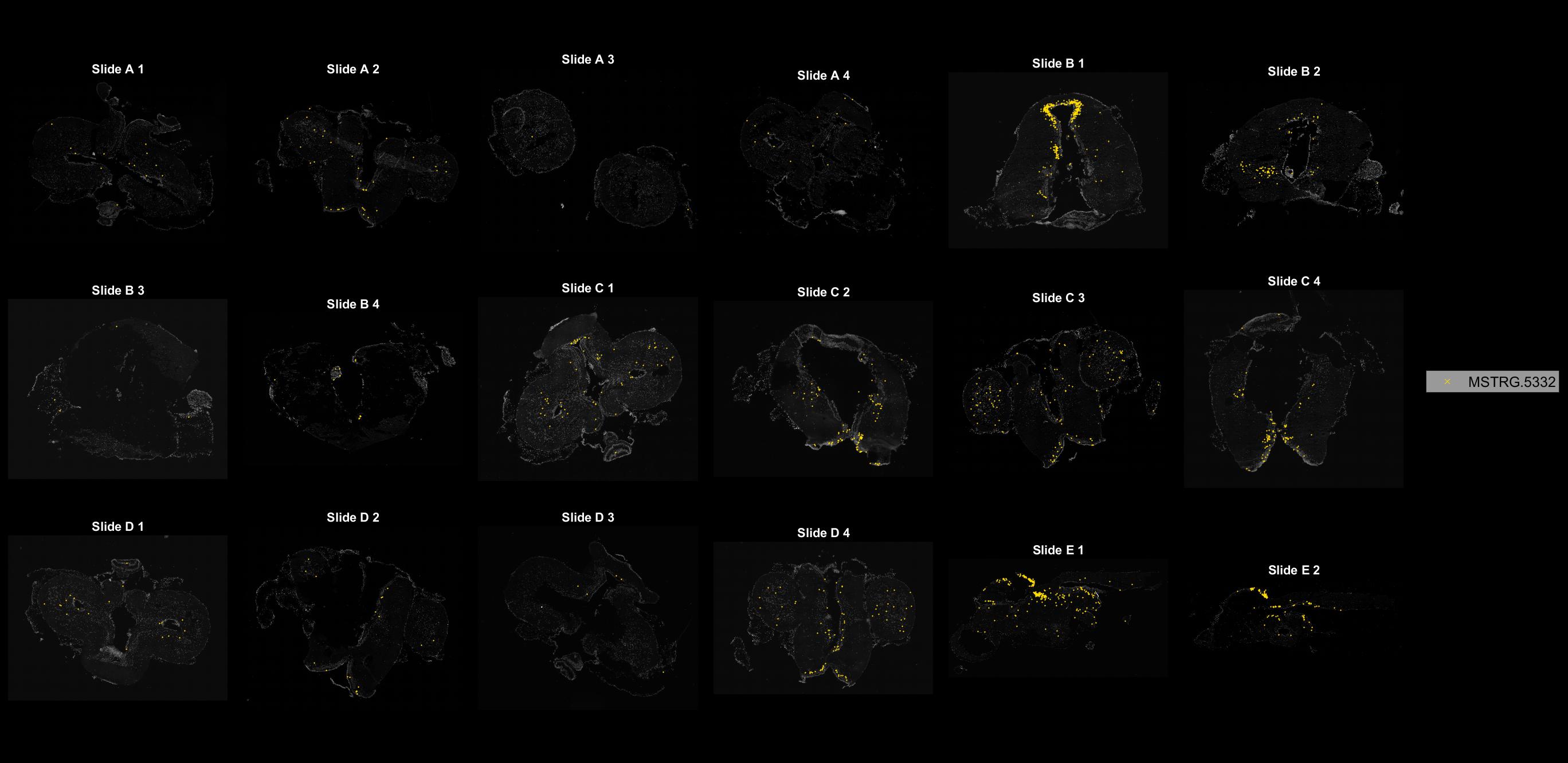

Supplement: Supplementary file 6 — In situ images produced in this study. [file 41559_2023_2170_MOESM6_ESM.zip › ISS/MSTRG.5332.jpg]

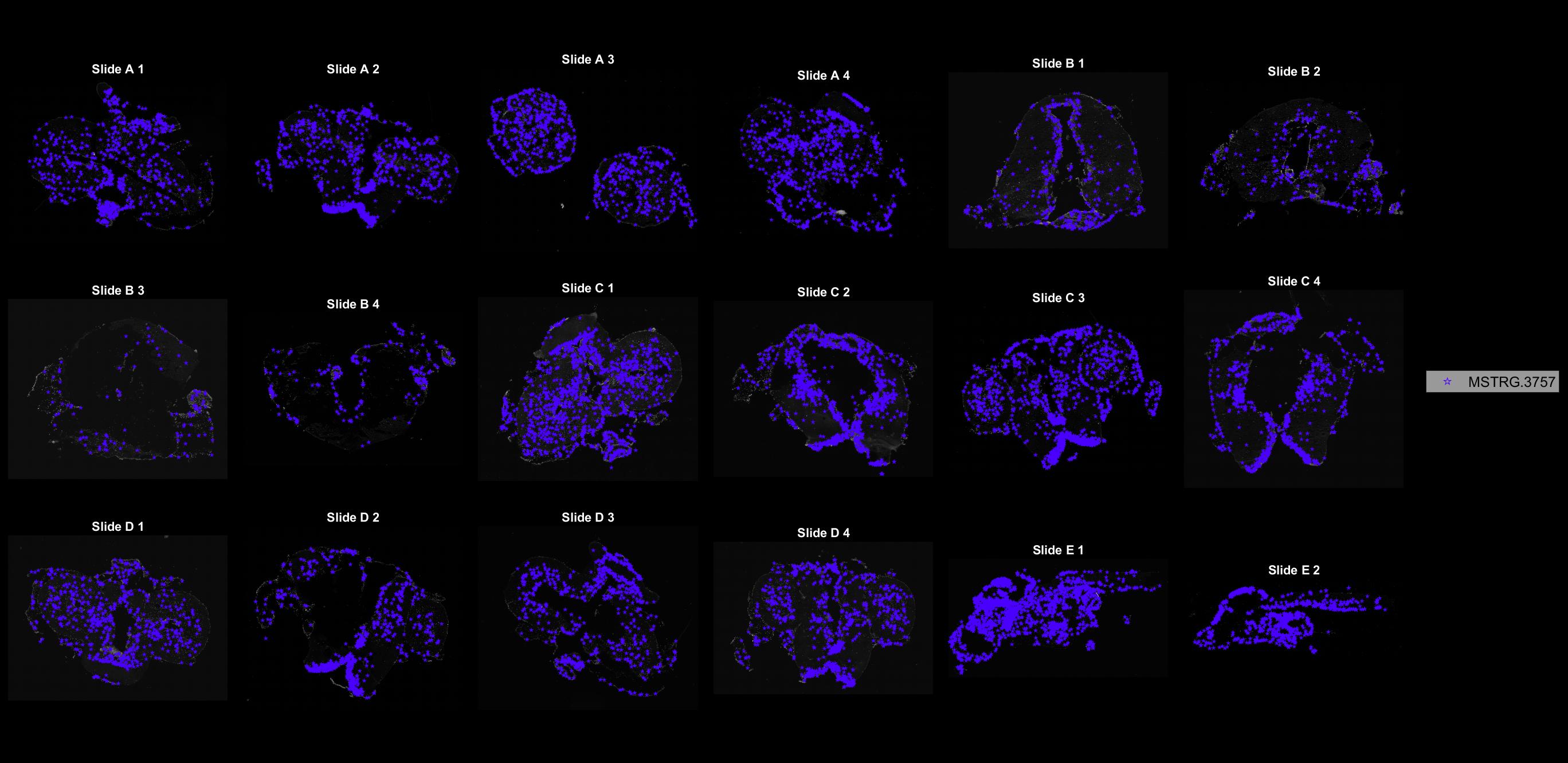

Supplement: Supplementary file 6 — In situ images produced in this study. [file 41559_2023_2170_MOESM6_ESM.zip › ISS/MSTRG.3757.jpg]

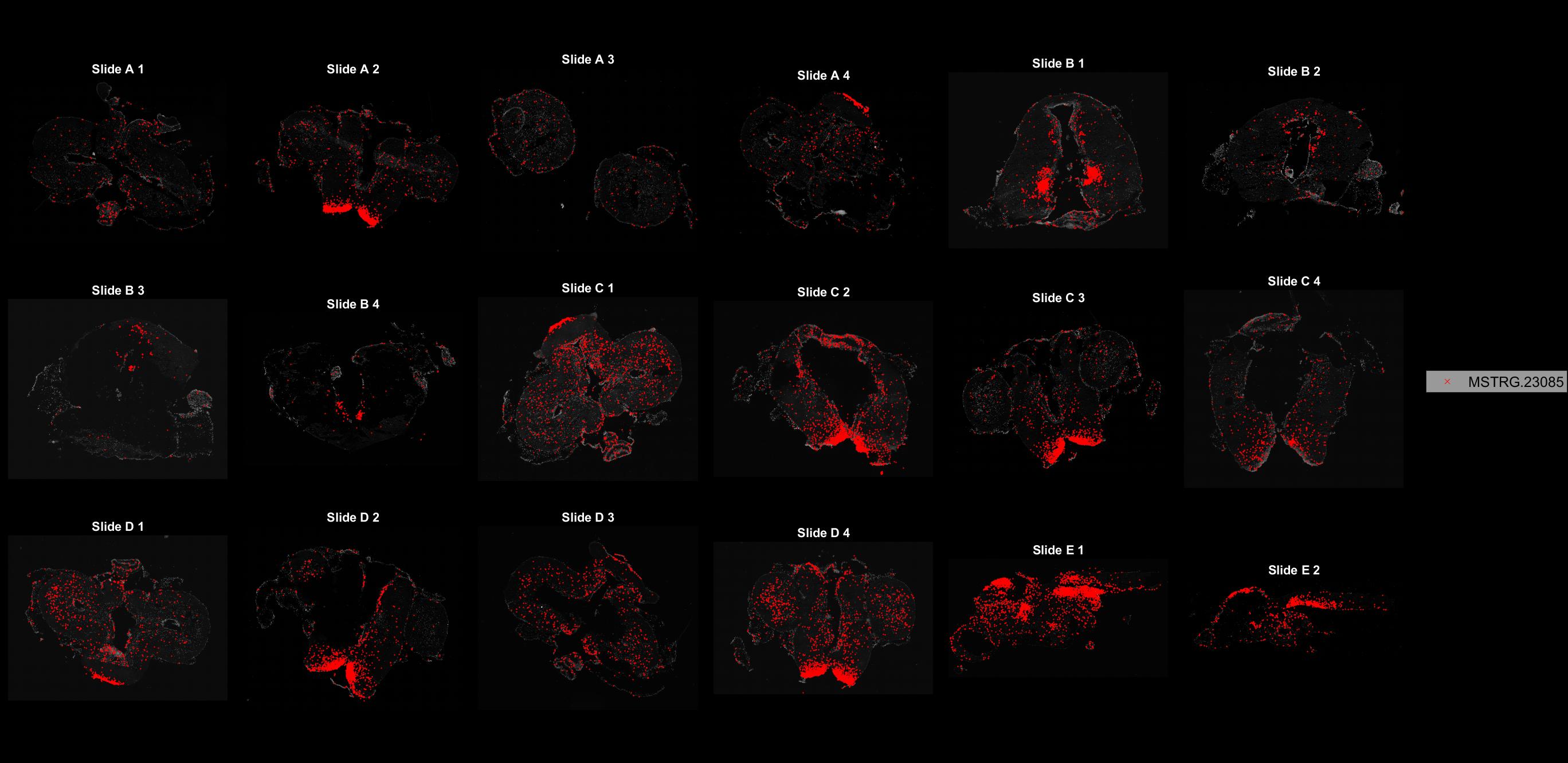

Supplement: Supplementary file 6 — In situ images produced in this study. [file 41559_2023_2170_MOESM6_ESM.zip › ISS/MSTRG.23085.jpg]

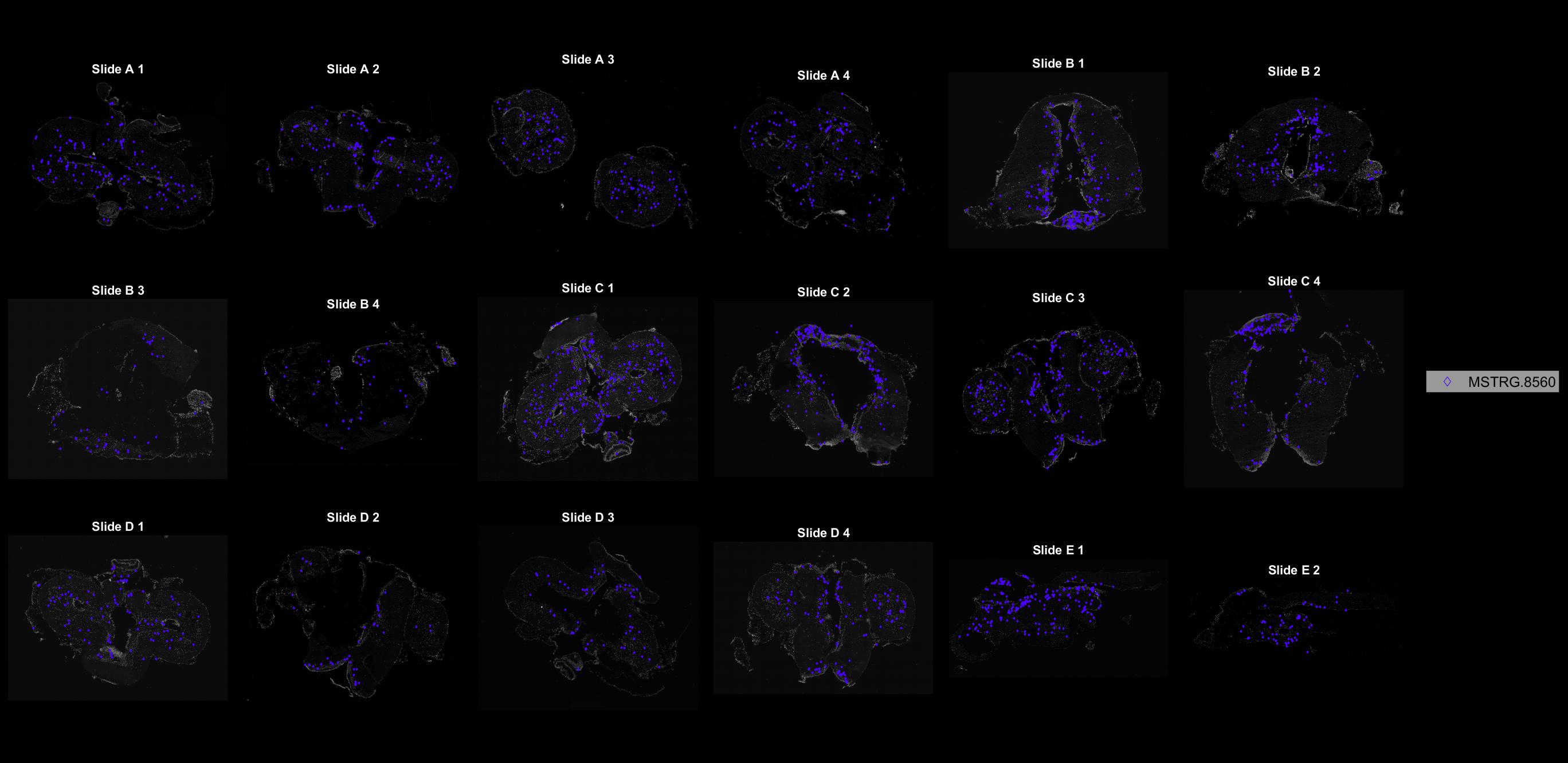

Supplement: Supplementary file 6 — In situ images produced in this study. [file 41559_2023_2170_MOESM6_ESM.zip › ISS/MSTRG.8560.jpg]

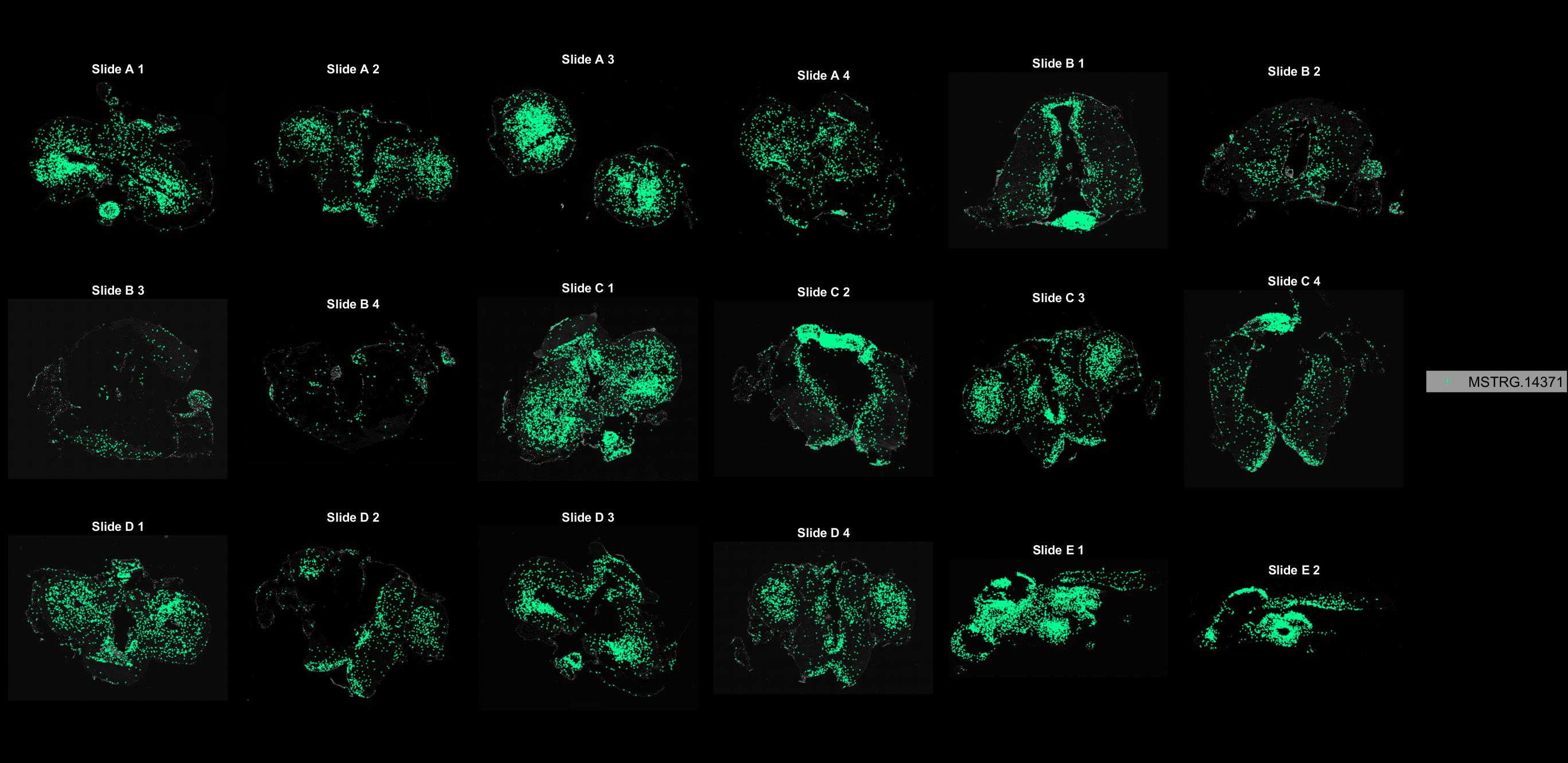

Supplement: Supplementary file 6 — In situ images produced in this study. [file 41559_2023_2170_MOESM6_ESM.zip › ISS/MSTRG.14371.jpg]

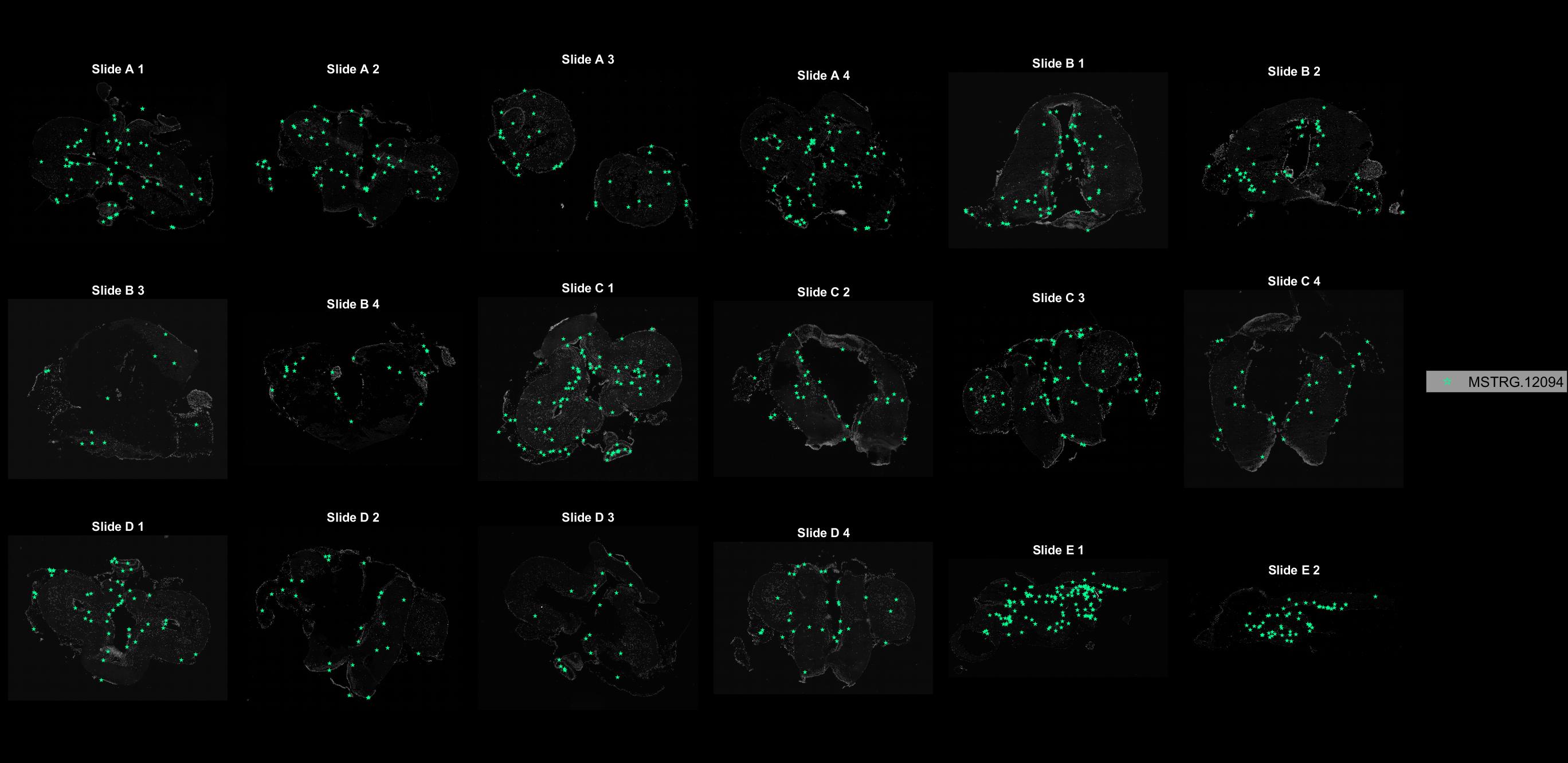

Supplement: Supplementary file 6 — In situ images produced in this study. [file 41559_2023_2170_MOESM6_ESM.zip › ISS/MSTRG.12094.jpg]

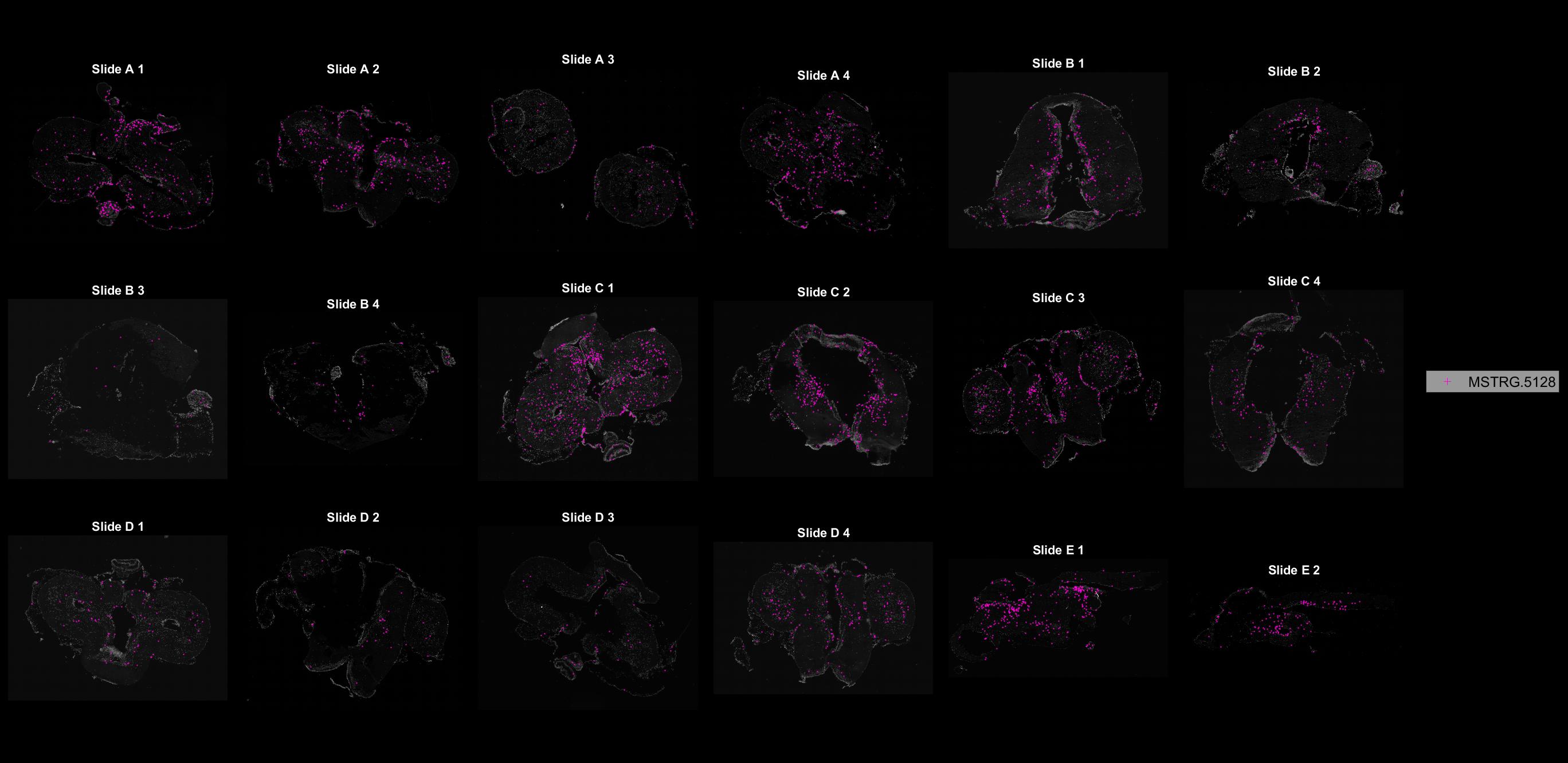

Supplement: Supplementary file 6 — In situ images produced in this study. [file 41559_2023_2170_MOESM6_ESM.zip › ISS/MSTRG.5128.jpg]

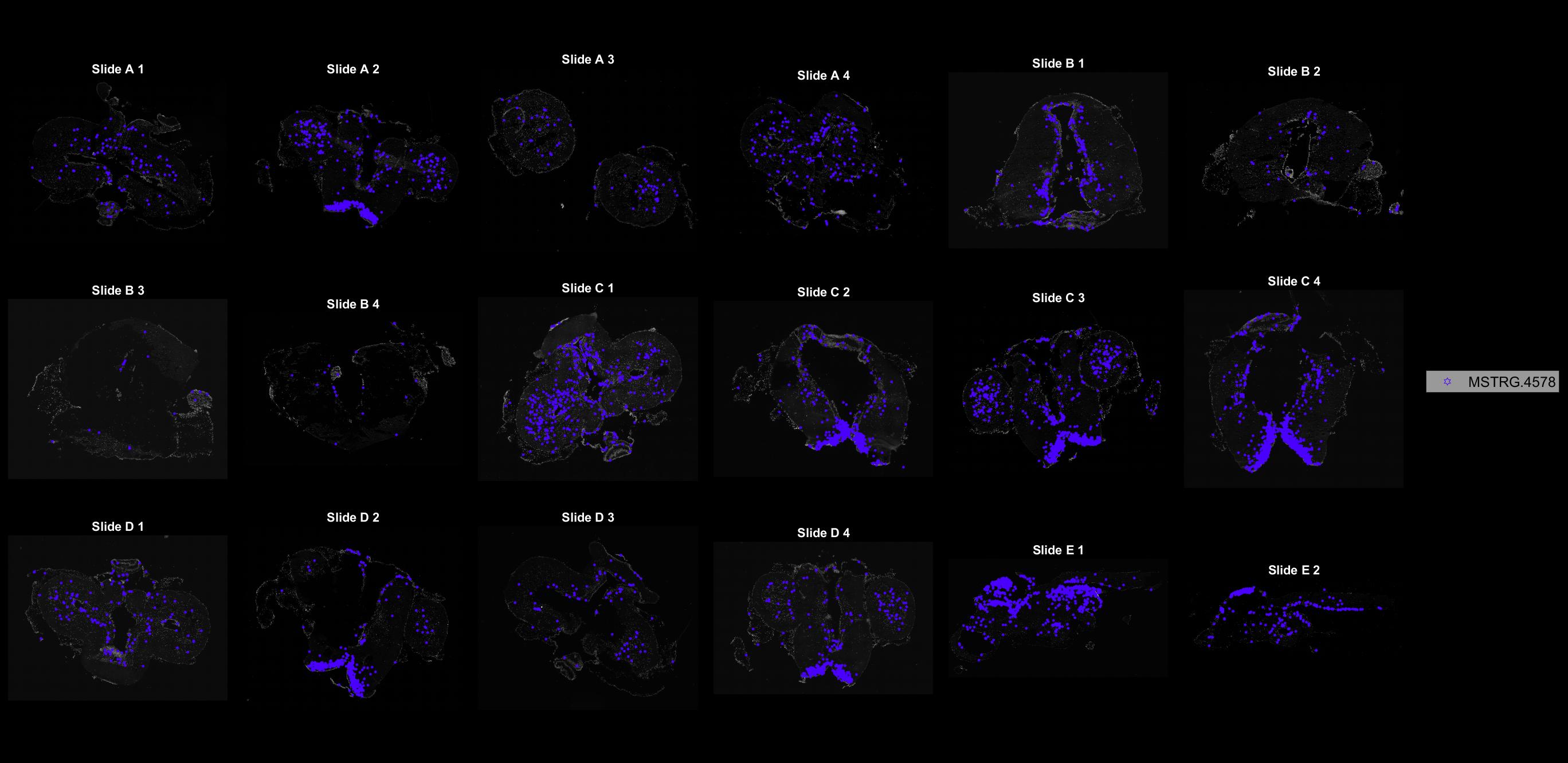

Supplement: Supplementary file 6 — In situ images produced in this study. [file 41559_2023_2170_MOESM6_ESM.zip › ISS/MSTRG.4578.jpg]

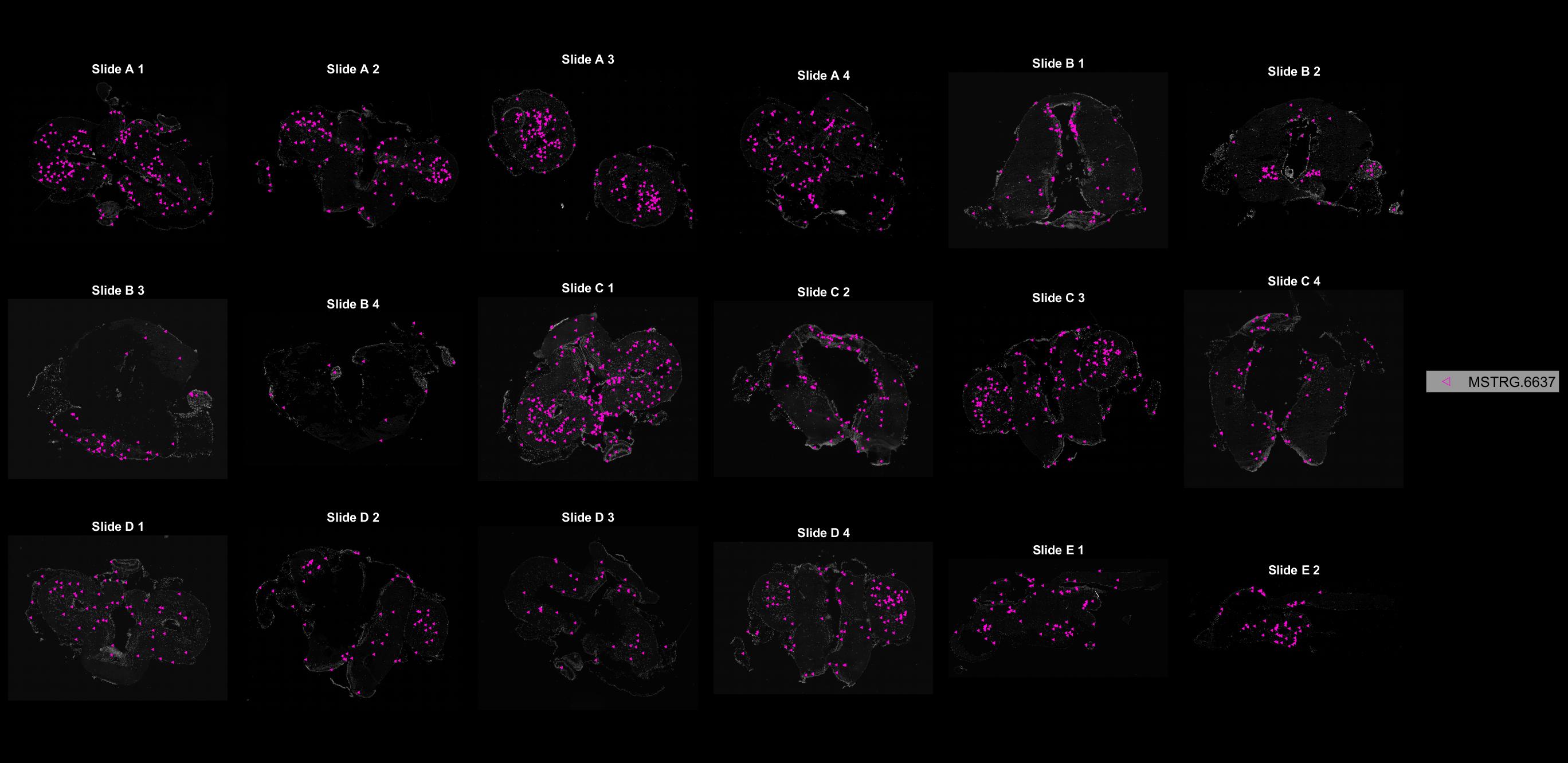

Supplement: Supplementary file 6 — In situ images produced in this study. [file 41559_2023_2170_MOESM6_ESM.zip › ISS/MSTRG.6637.jpg]

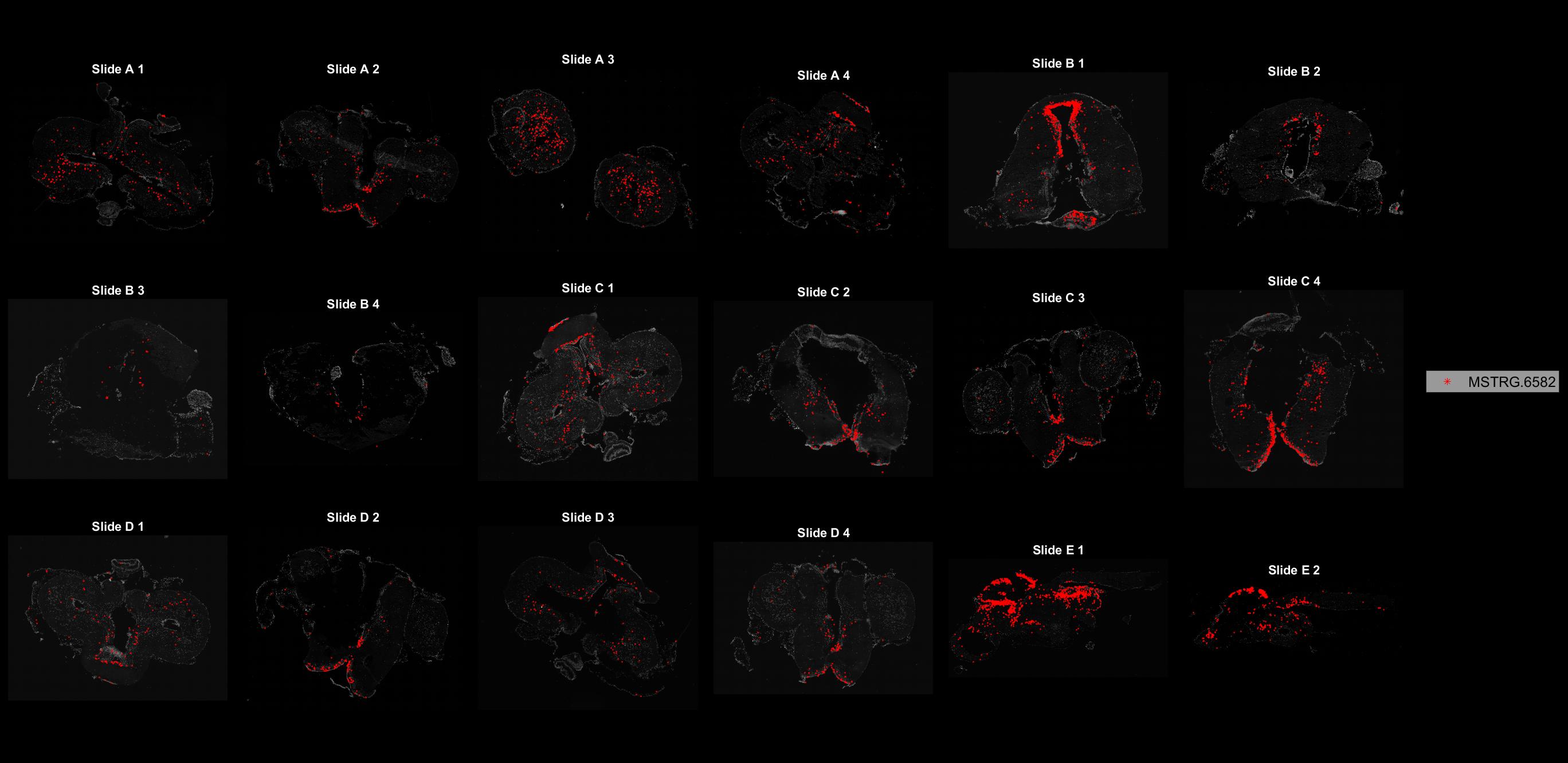

Supplement: Supplementary file 6 — In situ images produced in this study. [file 41559_2023_2170_MOESM6_ESM.zip › ISS/MSTRG.6582.jpg]

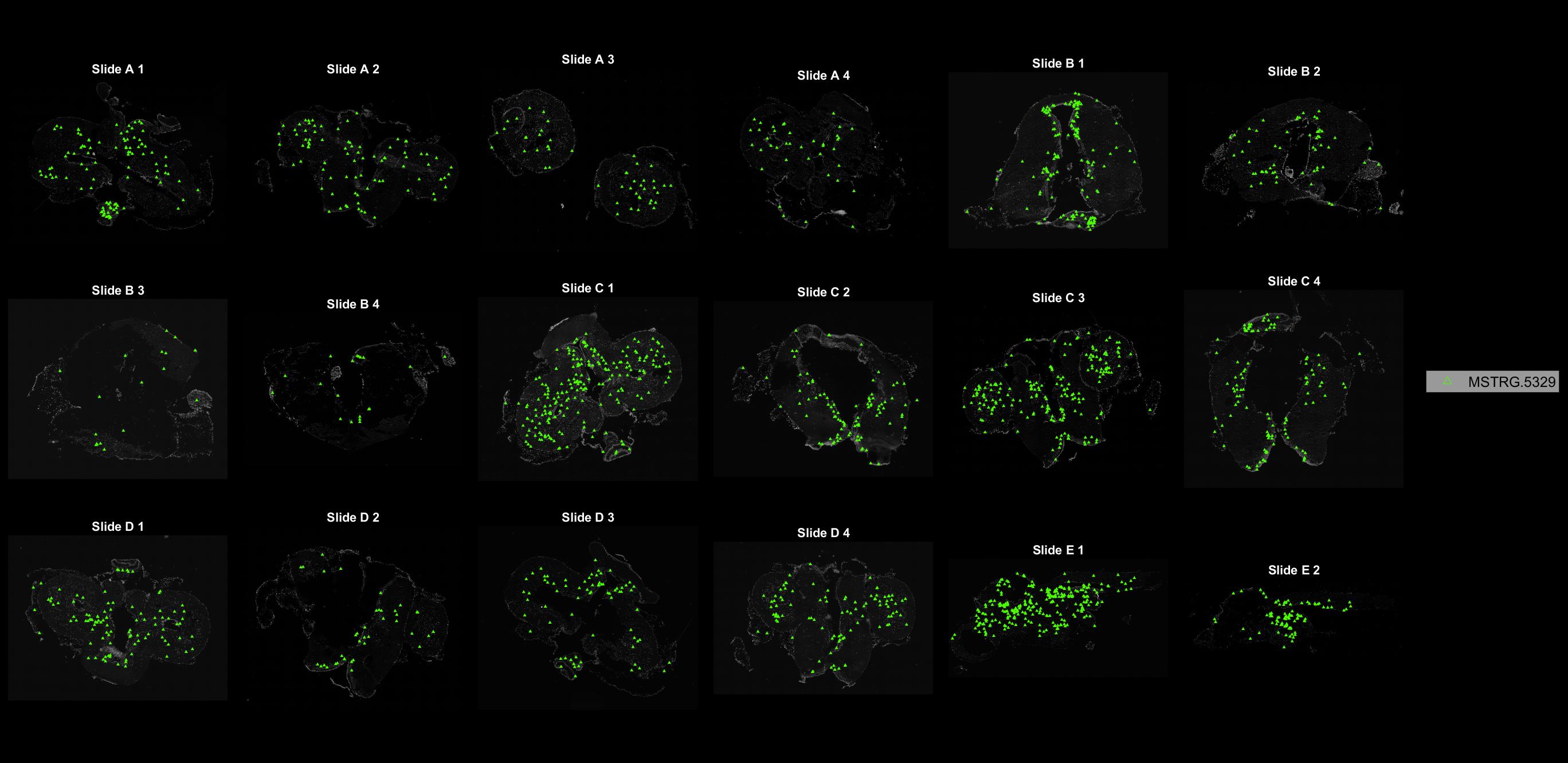

Supplement: Supplementary file 6 — In situ images produced in this study. [file 41559_2023_2170_MOESM6_ESM.zip › ISS/MSTRG.5329.jpg]

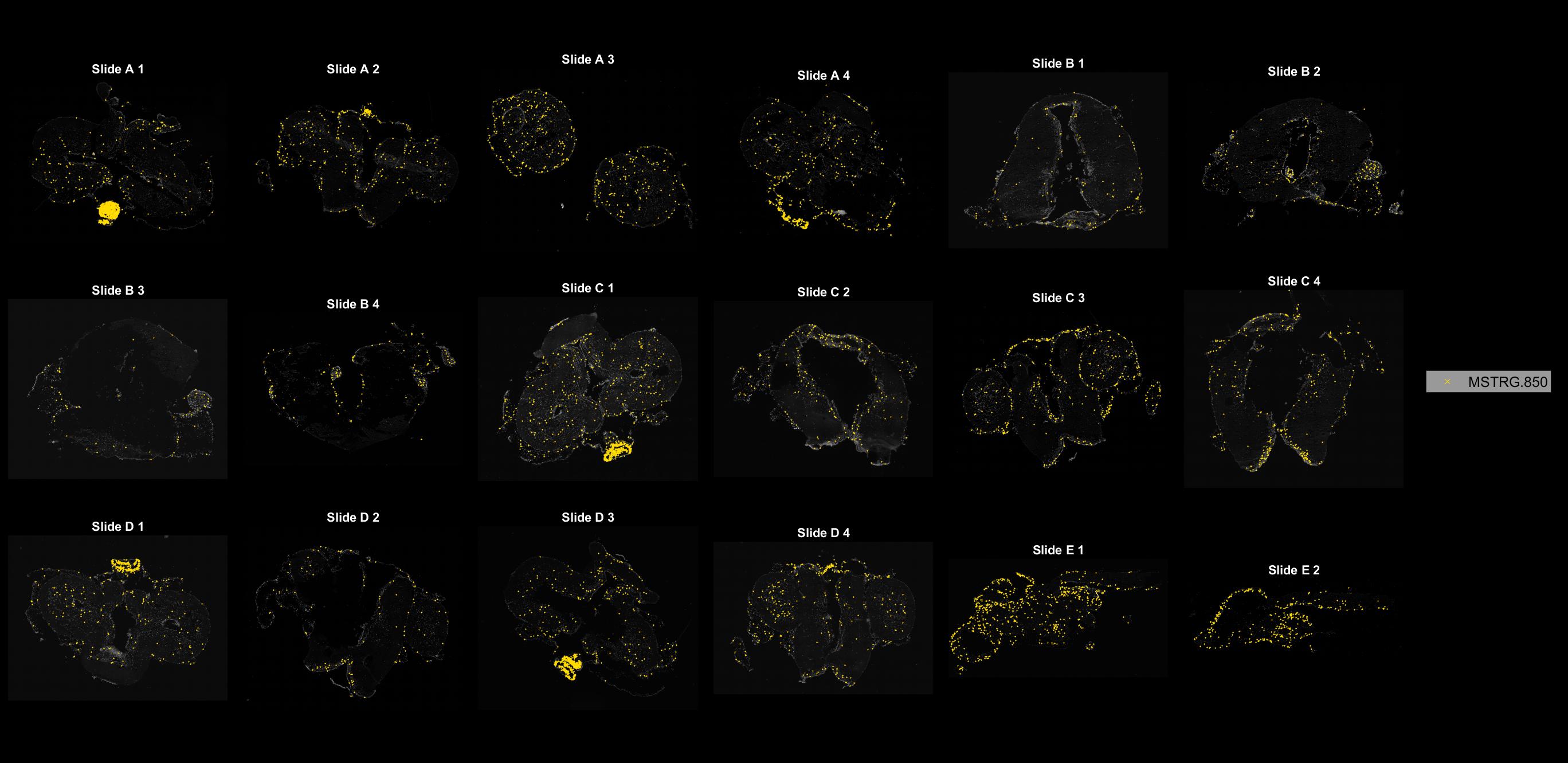

Supplement: Supplementary file 6 — In situ images produced in this study. [file 41559_2023_2170_MOESM6_ESM.zip › ISS/MSTRG.850.jpg]

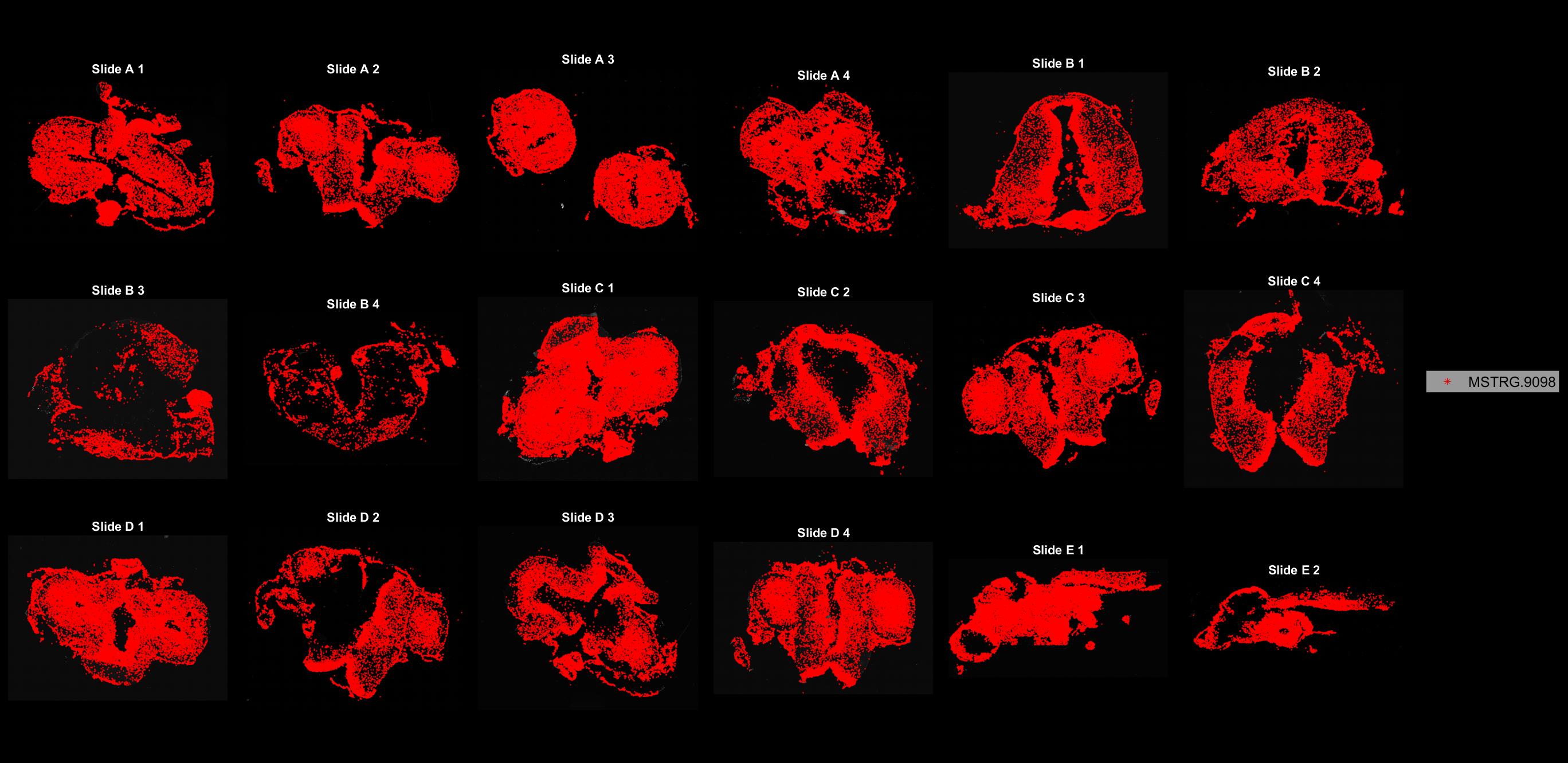

Supplement: Supplementary file 6 — In situ images produced in this study. [file 41559_2023_2170_MOESM6_ESM.zip › ISS/MSTRG.9098.jpg]

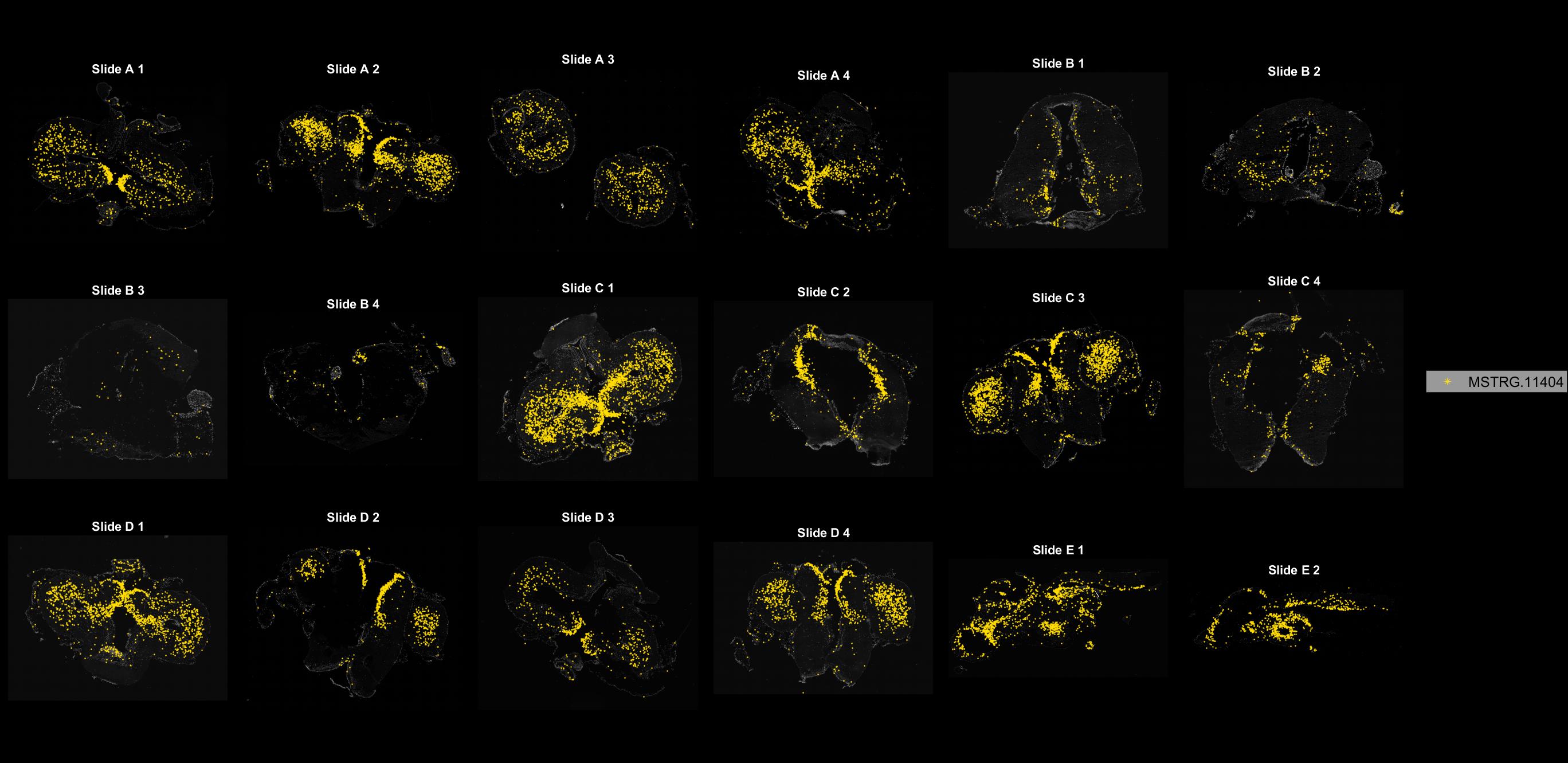

Supplement: Supplementary file 6 — In situ images produced in this study. [file 41559_2023_2170_MOESM6_ESM.zip › ISS/MSTRG.11404.jpg]

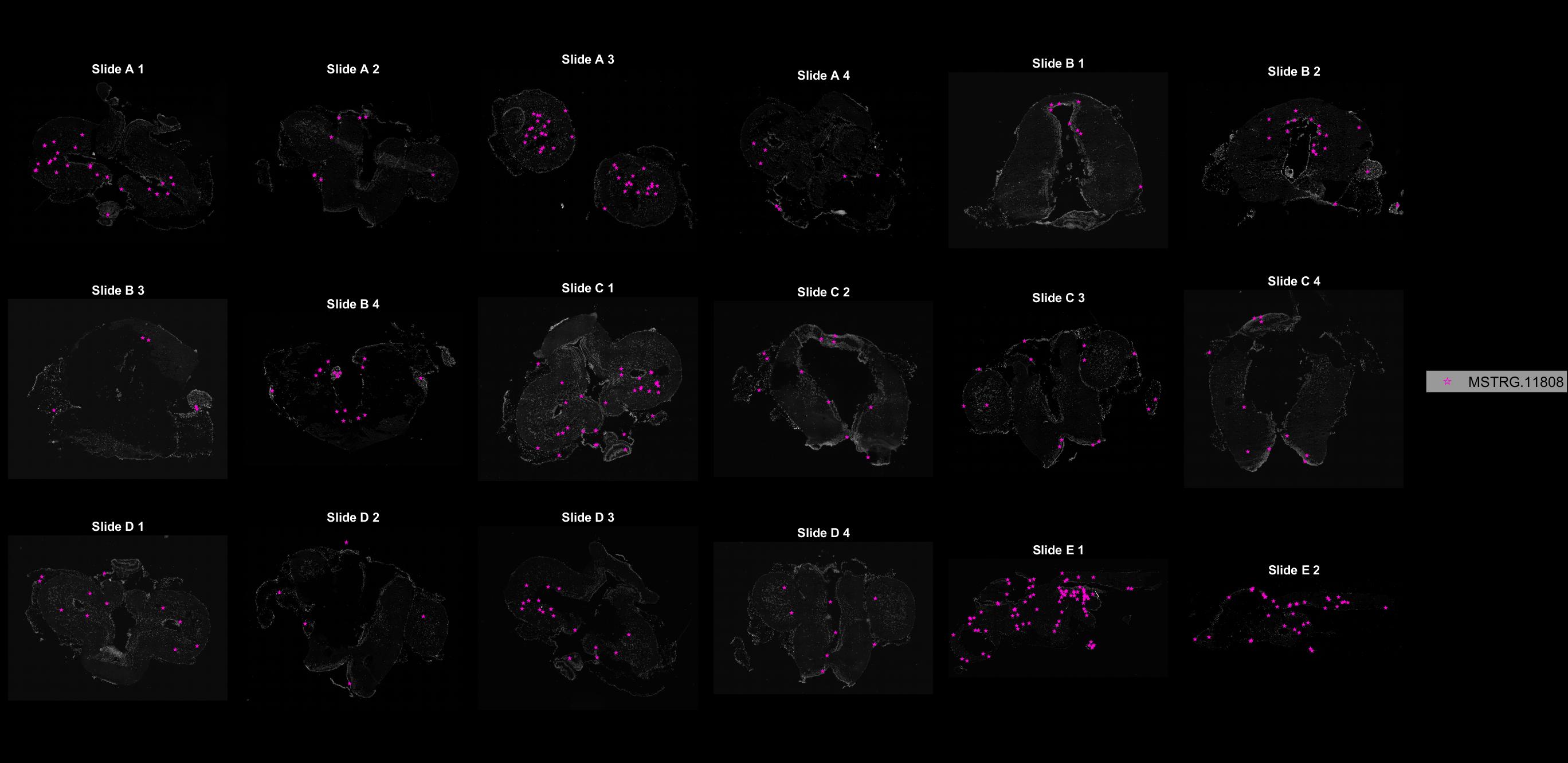

Supplement: Supplementary file 6 — In situ images produced in this study. [file 41559_2023_2170_MOESM6_ESM.zip › ISS/MSTRG.11808.jpg]

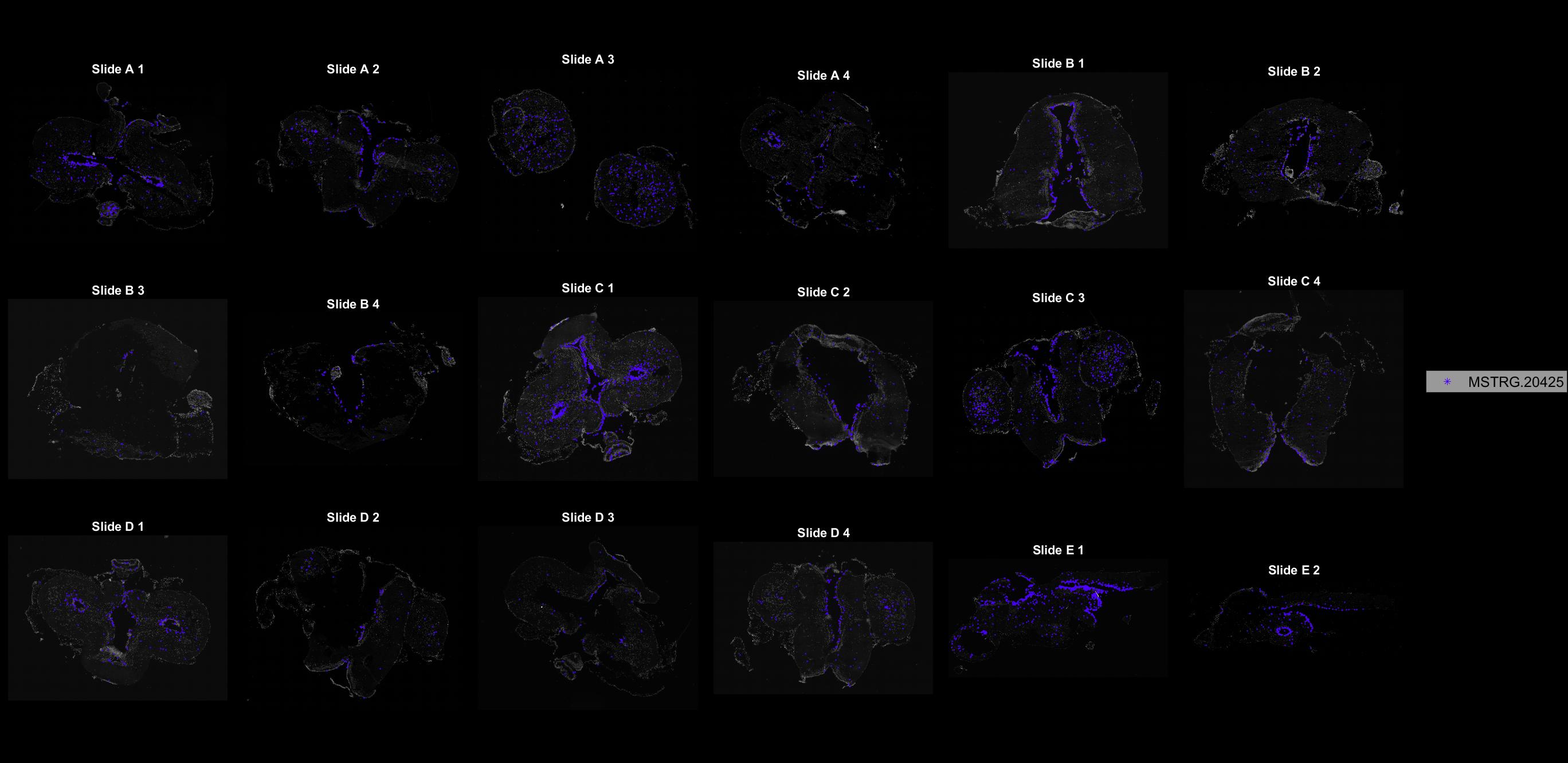

Supplement: Supplementary file 6 — In situ images produced in this study. [file 41559_2023_2170_MOESM6_ESM.zip › ISS/MSTRG.20425.jpg]

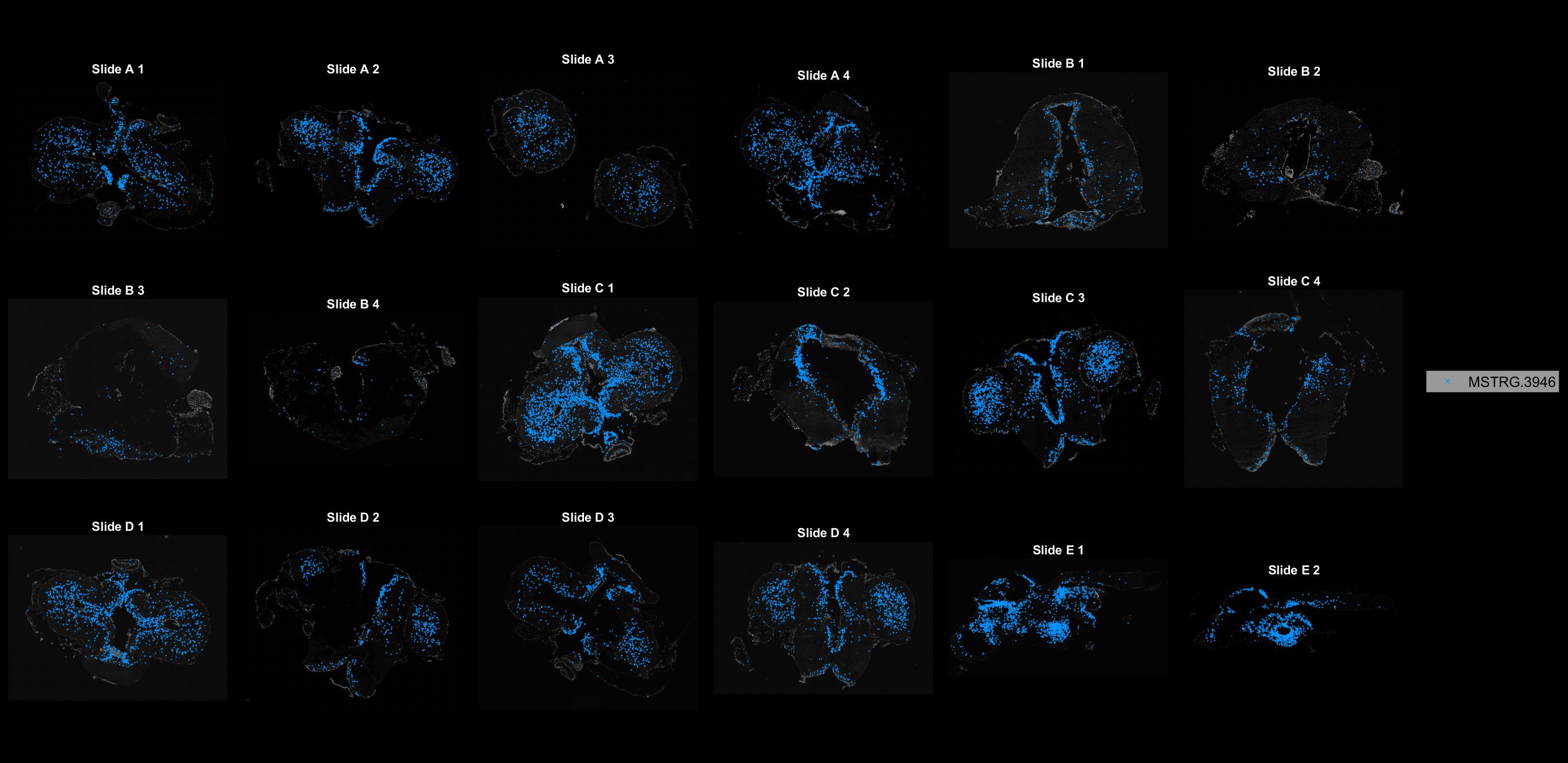

Supplement: Supplementary file 6 — In situ images produced in this study. [file 41559_2023_2170_MOESM6_ESM.zip › ISS/MSTRG.3946.jpg]

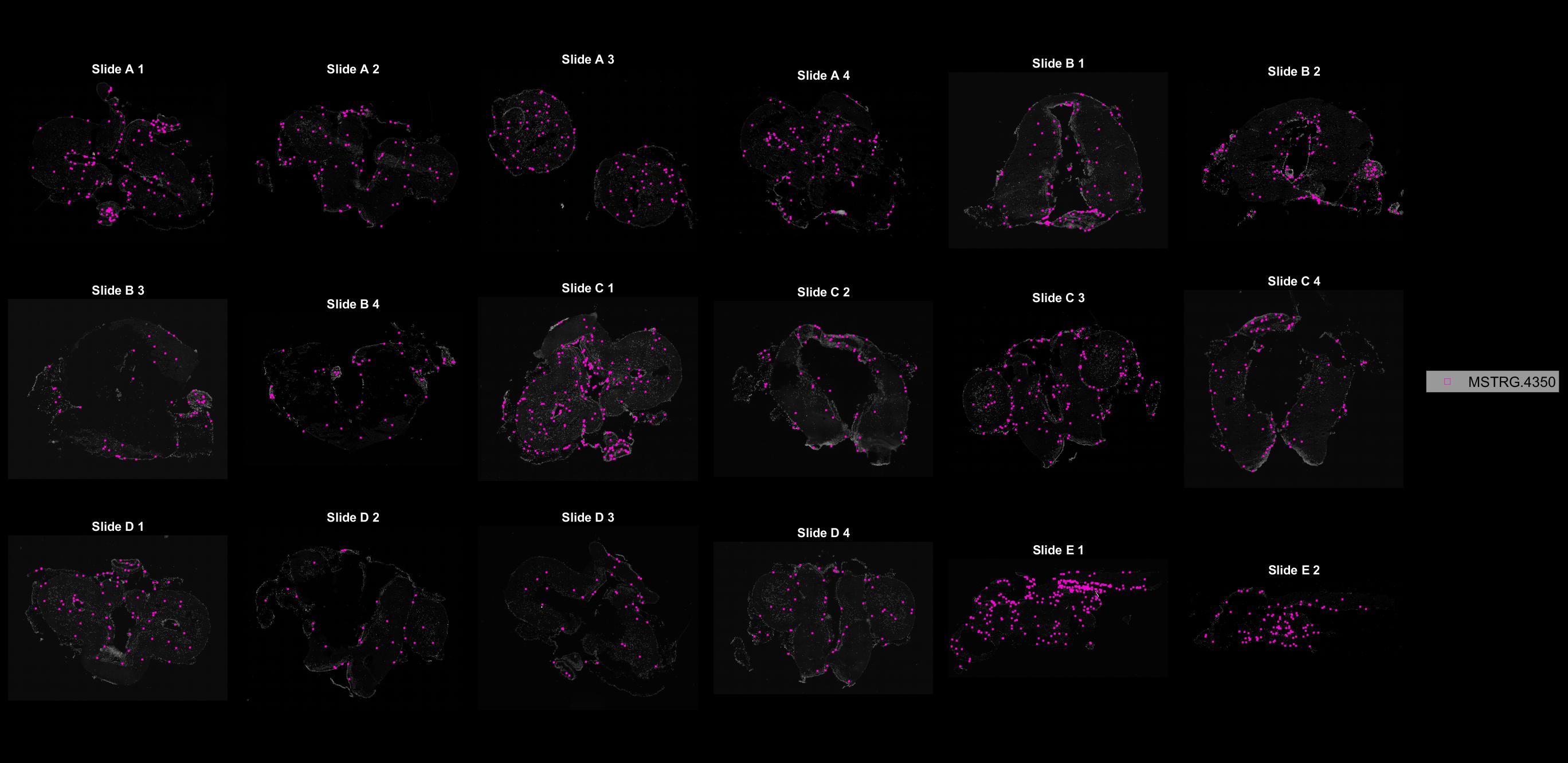

Supplement: Supplementary file 6 — In situ images produced in this study. [file 41559_2023_2170_MOESM6_ESM.zip › ISS/MSTRG.4350.jpg]

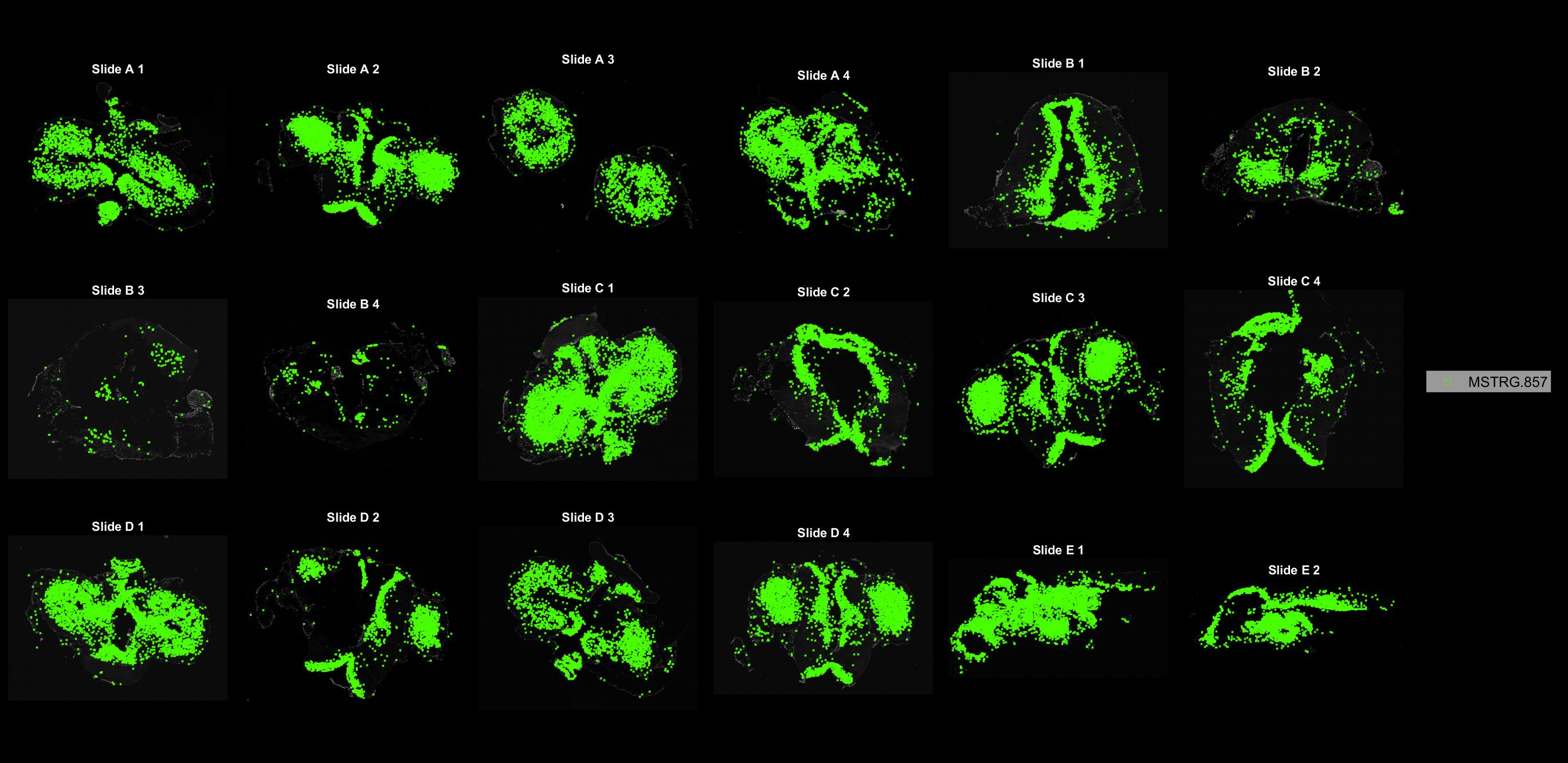

Supplement: Supplementary file 6 — In situ images produced in this study. [file 41559_2023_2170_MOESM6_ESM.zip › ISS/MSTRG.857.jpg]

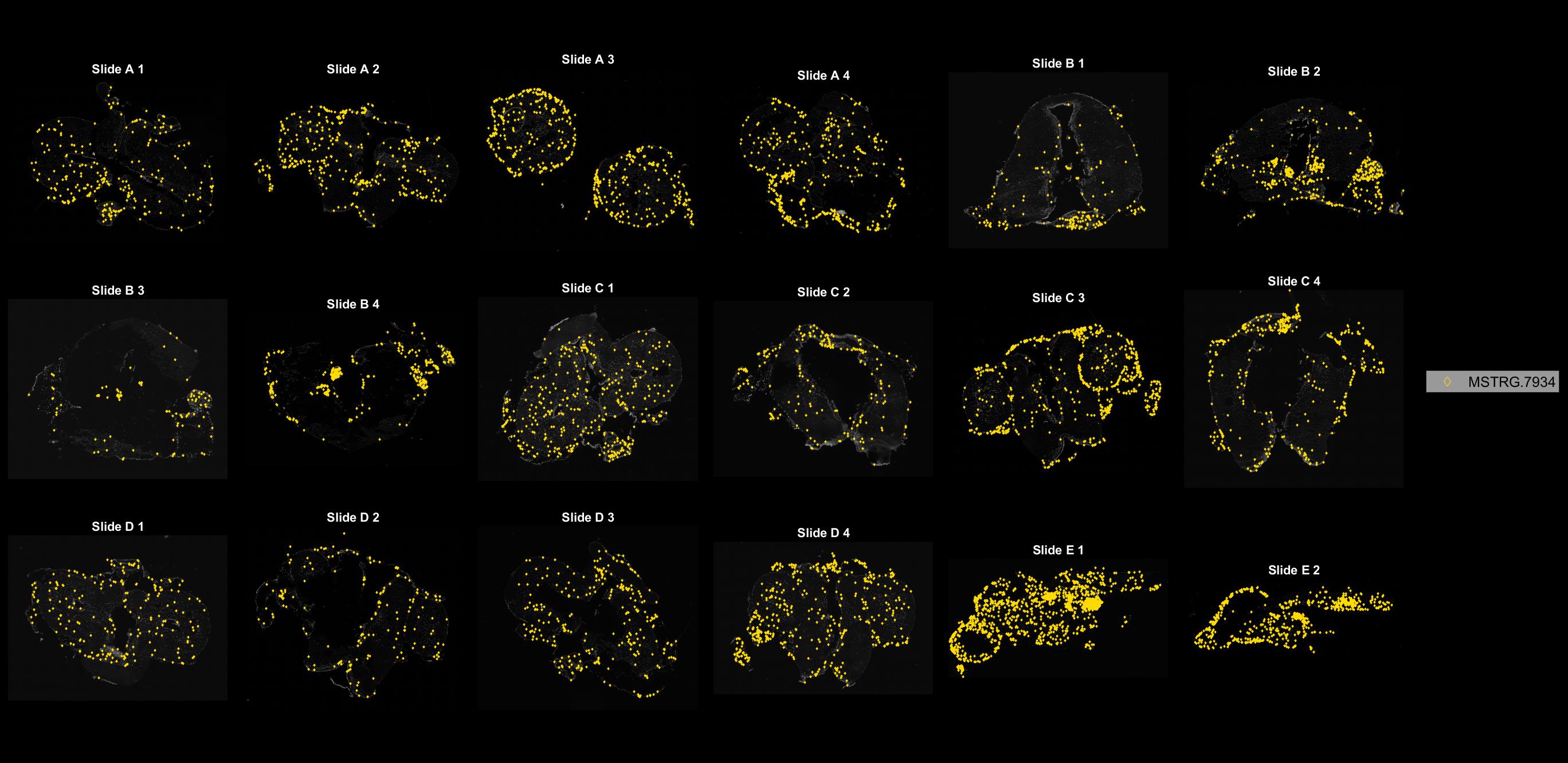

Supplement: Supplementary file 6 — In situ images produced in this study. [file 41559_2023_2170_MOESM6_ESM.zip › ISS/MSTRG.7934.jpg]

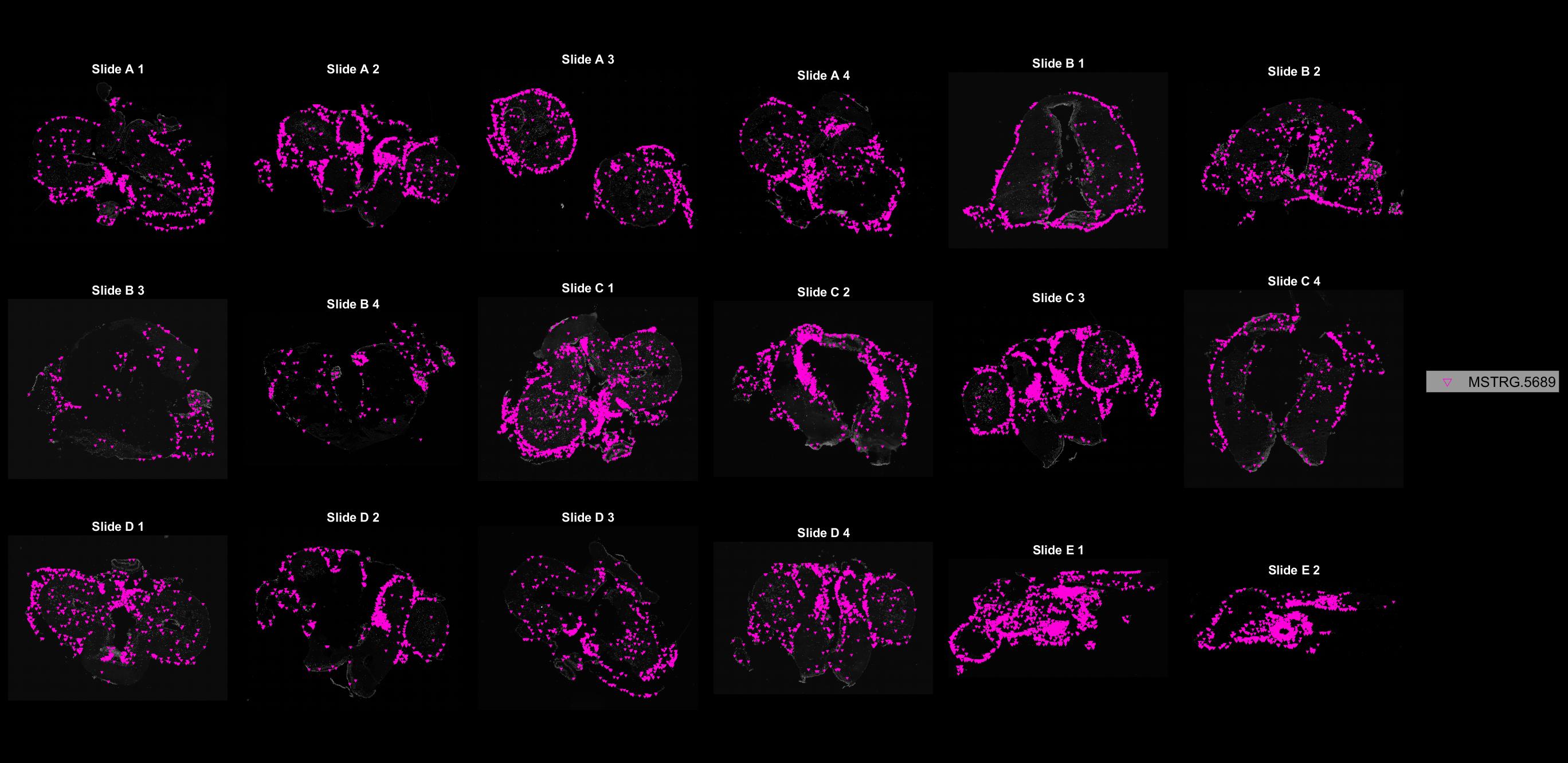

Supplement: Supplementary file 6 — In situ images produced in this study. [file 41559_2023_2170_MOESM6_ESM.zip › ISS/MSTRG.5689.jpg]

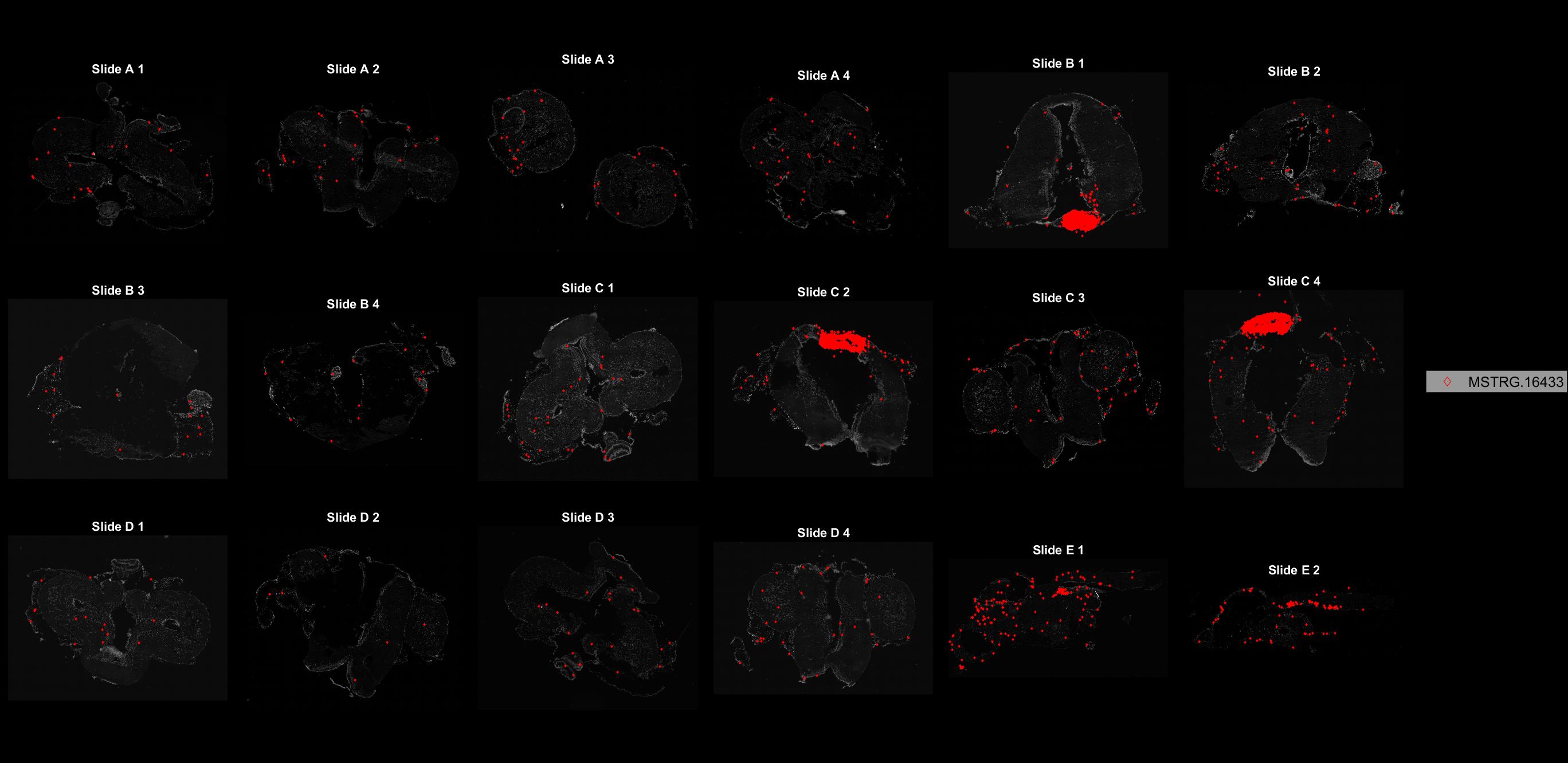

Supplement: Supplementary file 6 — In situ images produced in this study. [file 41559_2023_2170_MOESM6_ESM.zip › ISS/MSTRG.16433.jpg]

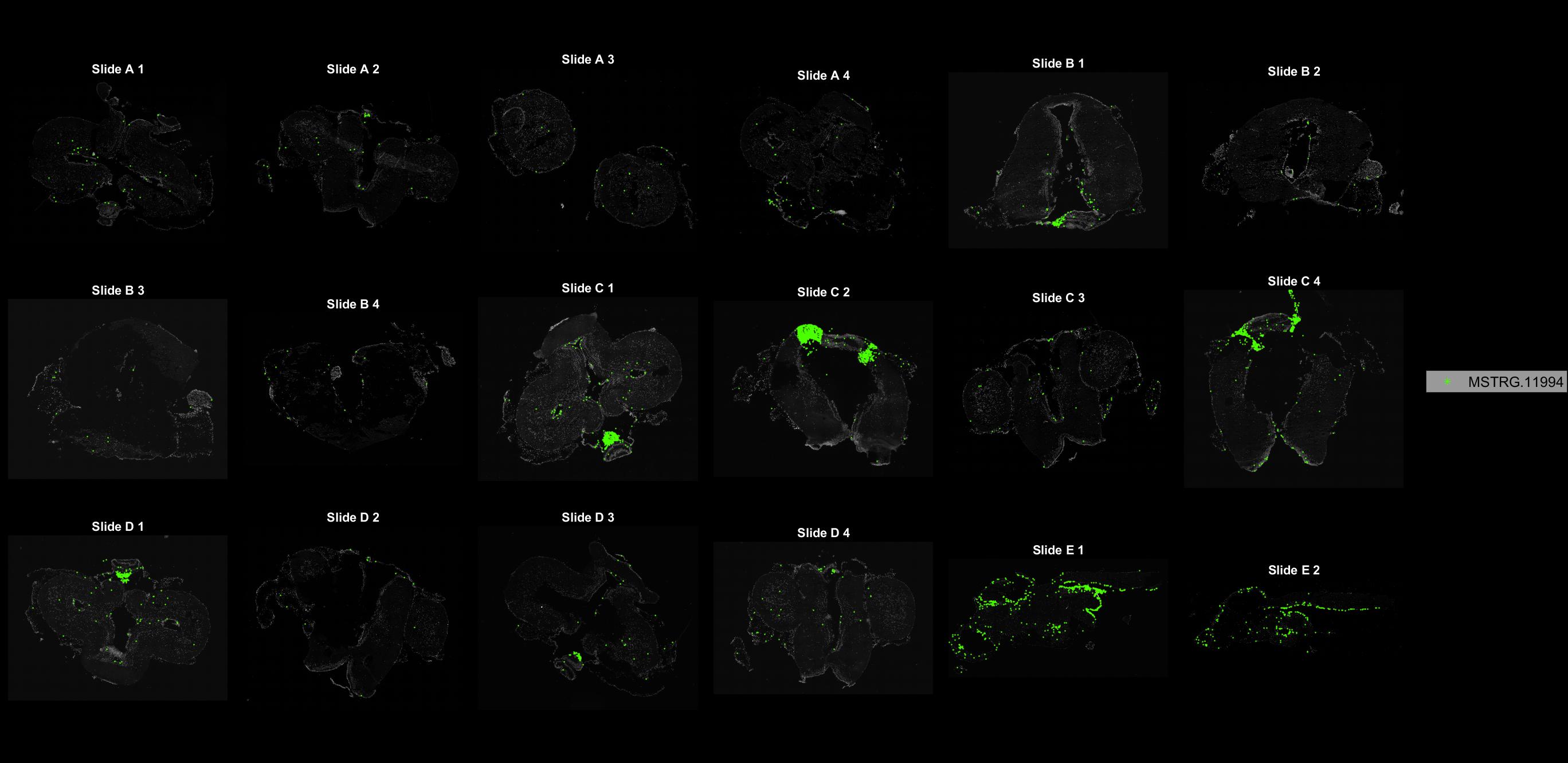

Supplement: Supplementary file 6 — In situ images produced in this study. [file 41559_2023_2170_MOESM6_ESM.zip › ISS/MSTRG.11994.jpg]

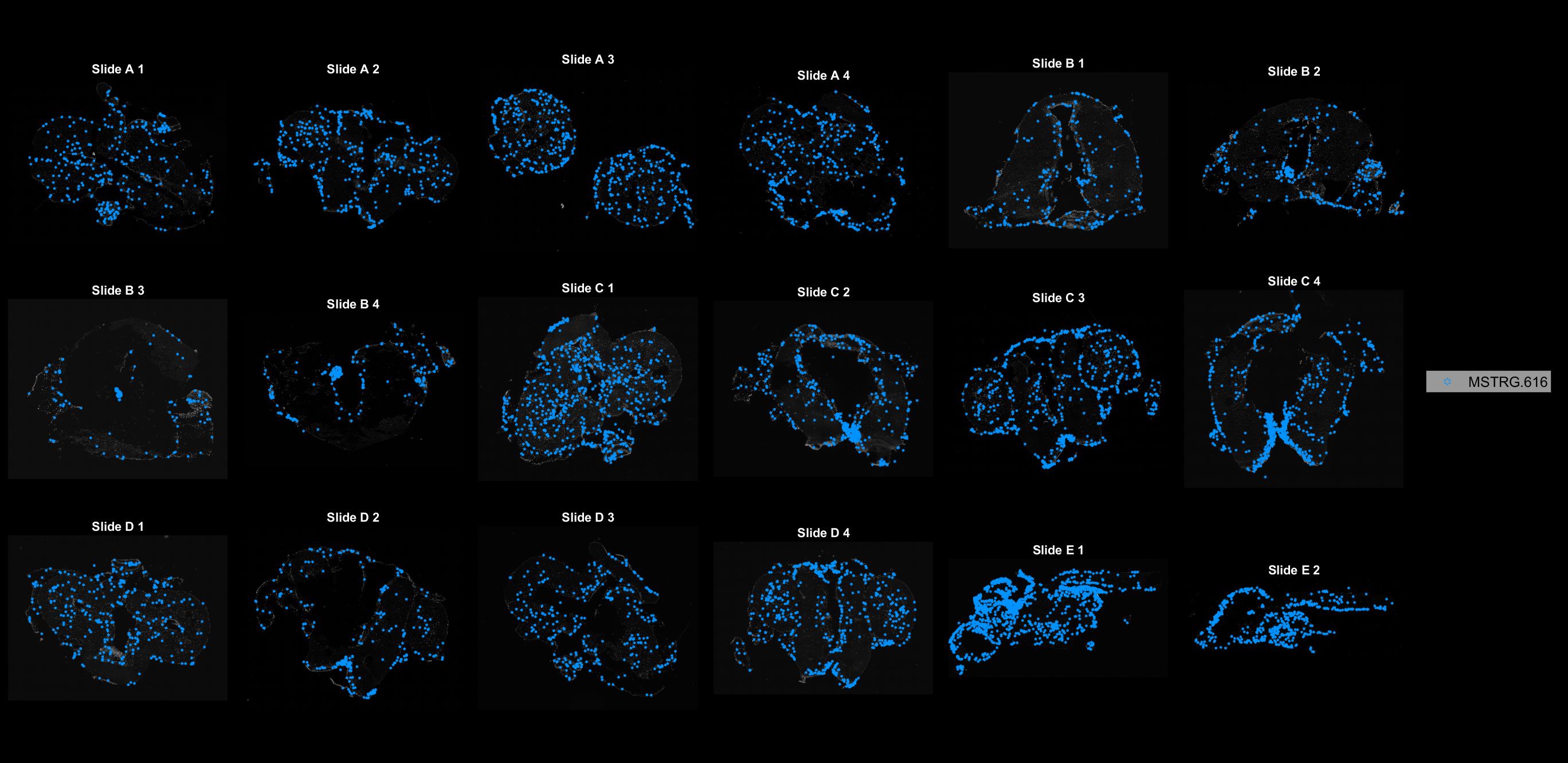

Supplement: Supplementary file 6 — In situ images produced in this study. [file 41559_2023_2170_MOESM6_ESM.zip › ISS/MSTRG.616.jpg]

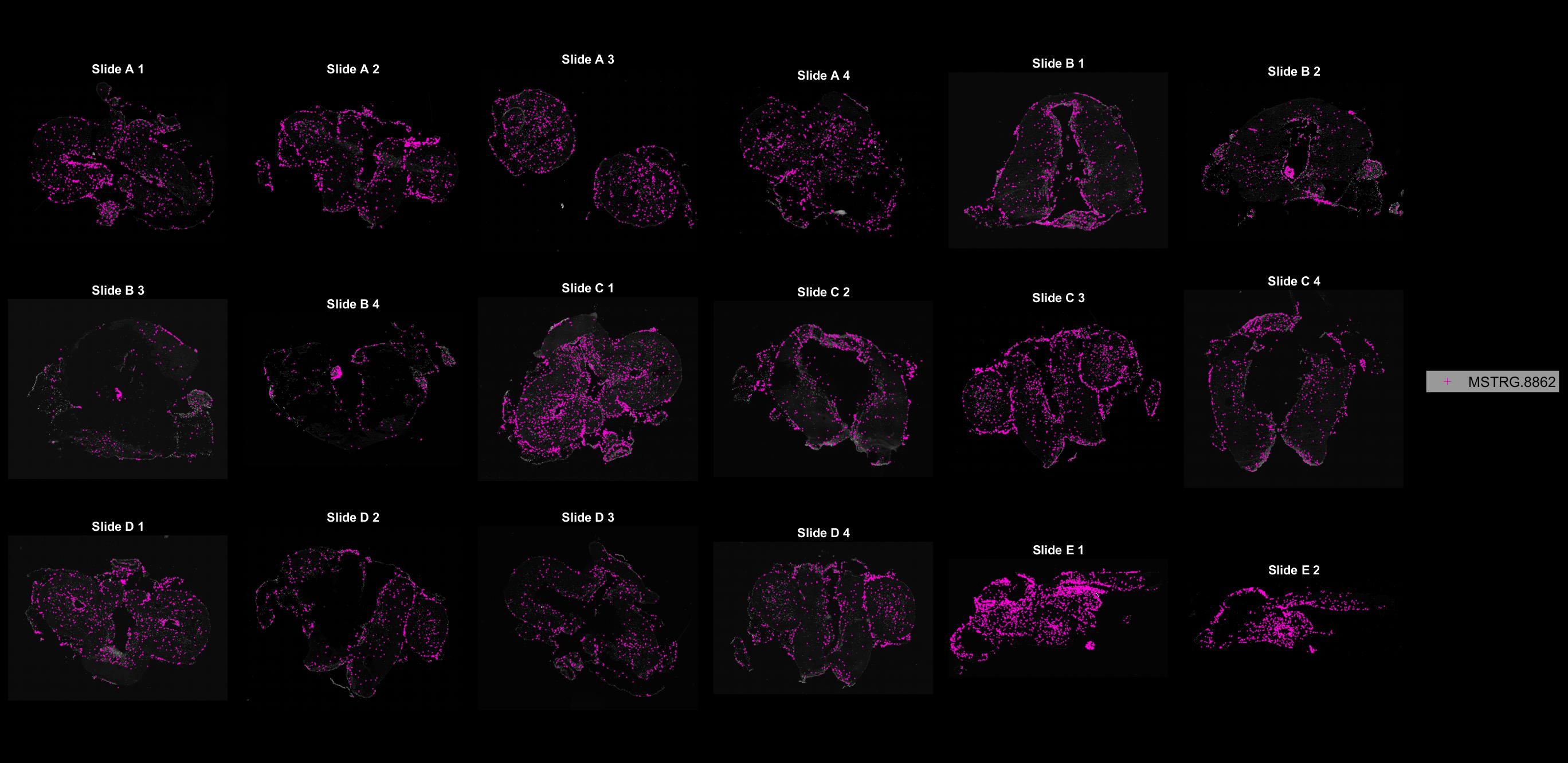

Supplement: Supplementary file 6 — In situ images produced in this study. [file 41559_2023_2170_MOESM6_ESM.zip › ISS/MSTRG.8862.jpg]

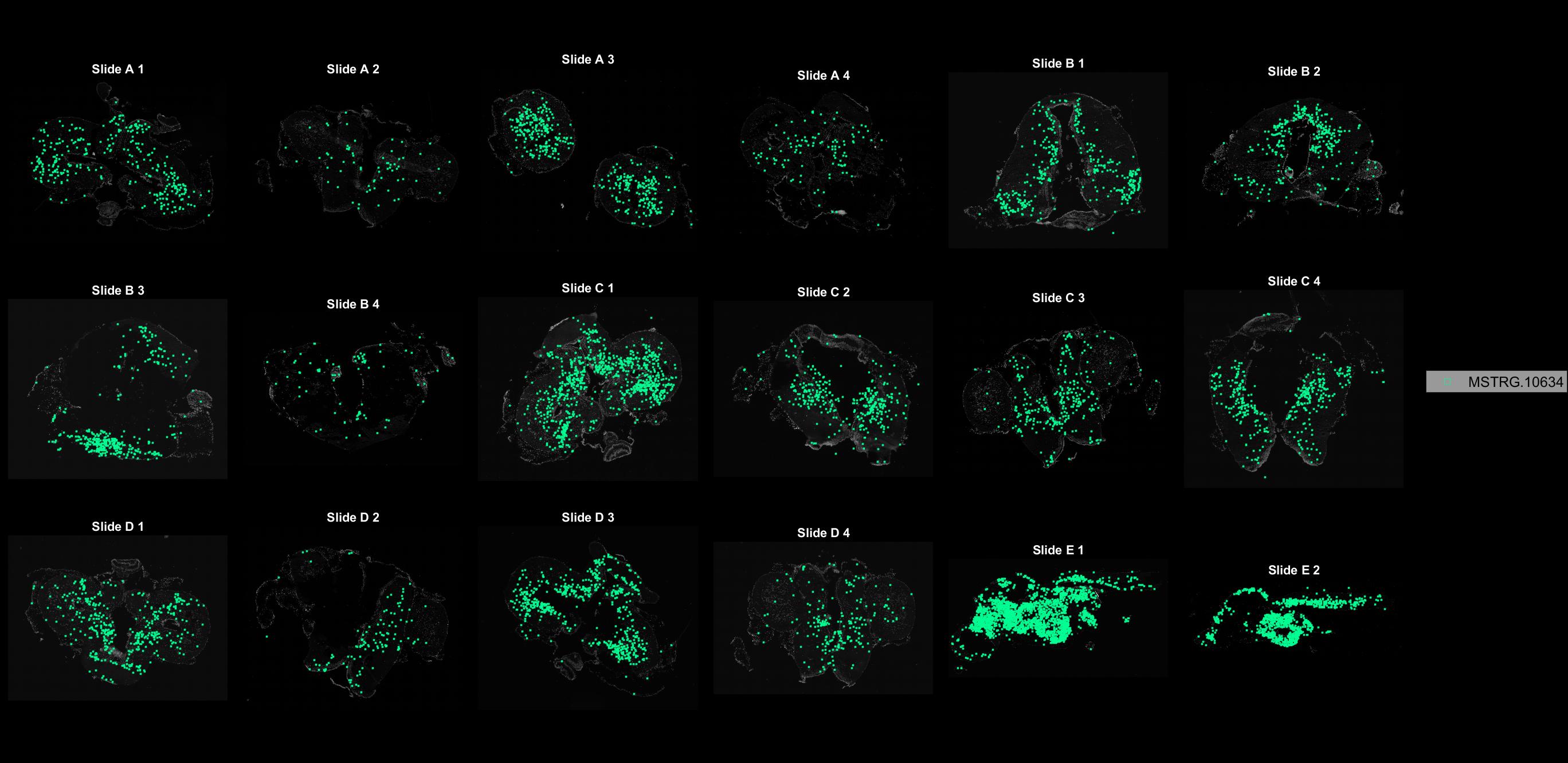

Supplement: Supplementary file 6 — In situ images produced in this study. [file 41559_2023_2170_MOESM6_ESM.zip › ISS/MSTRG.10634.jpg]

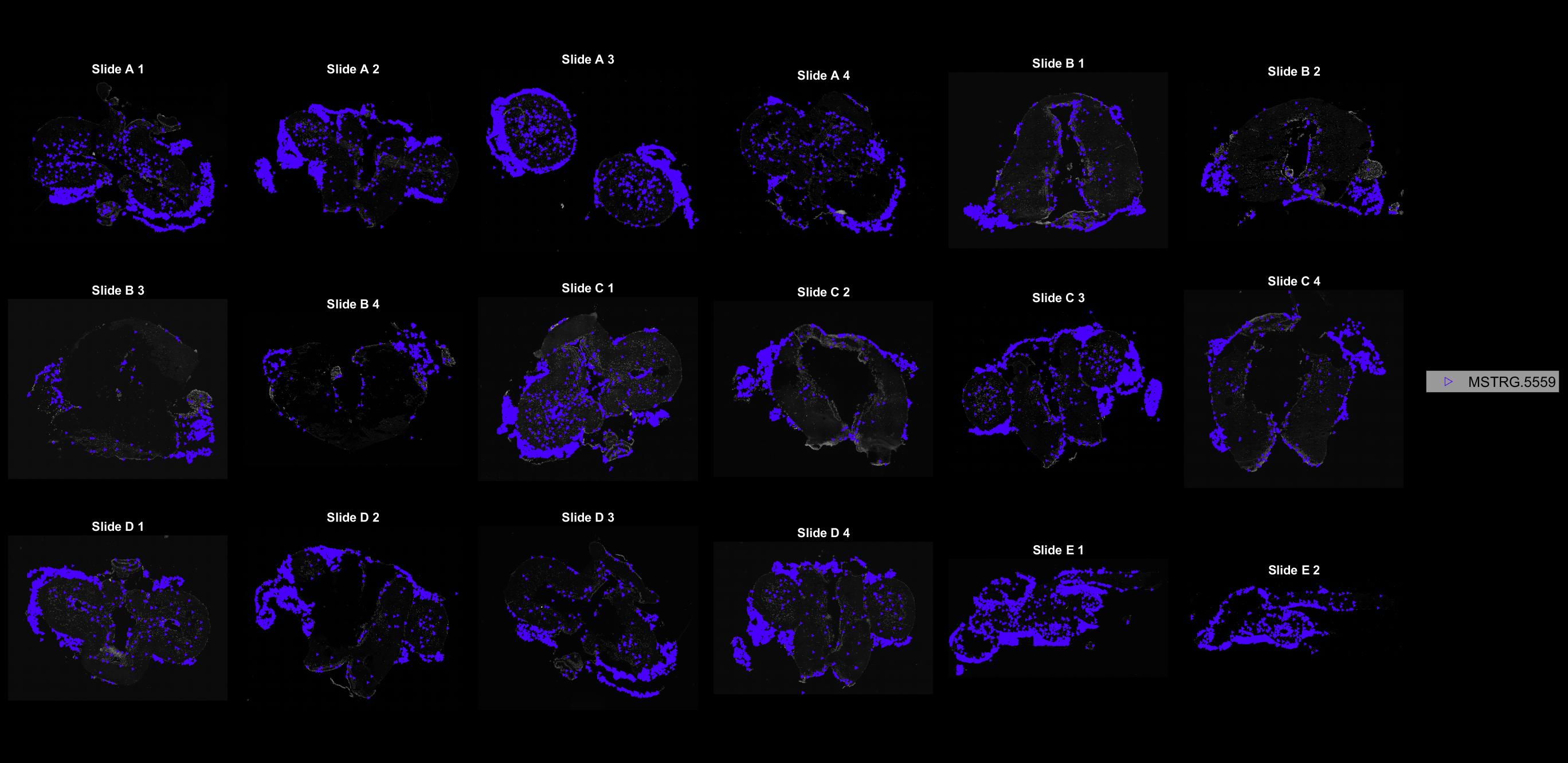

Supplement: Supplementary file 6 — In situ images produced in this study. [file 41559_2023_2170_MOESM6_ESM.zip › ISS/MSTRG.5559.jpg]

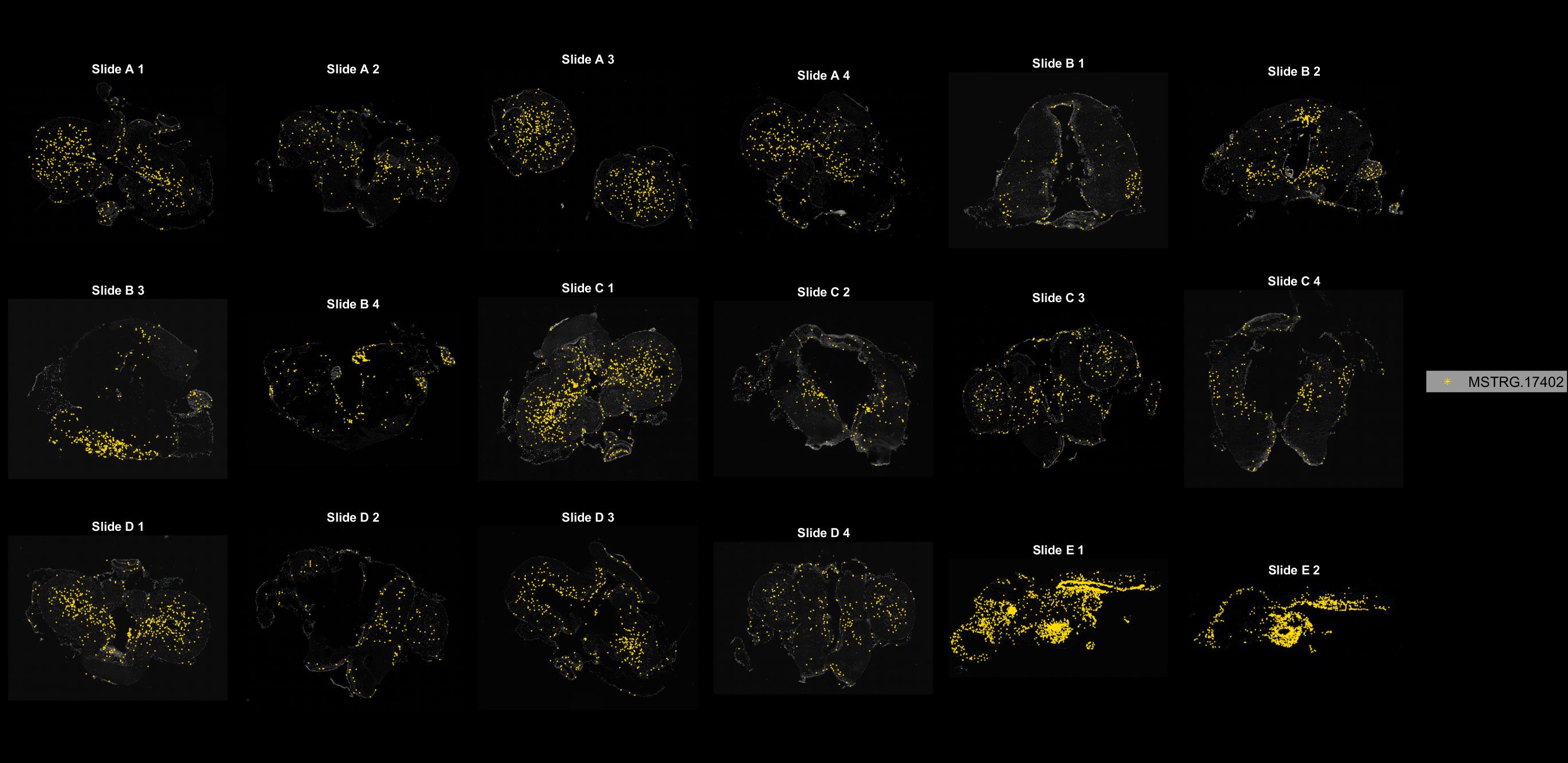

Supplement: Supplementary file 6 — In situ images produced in this study. [file 41559_2023_2170_MOESM6_ESM.zip › ISS/MSTRG.17402.jpg]

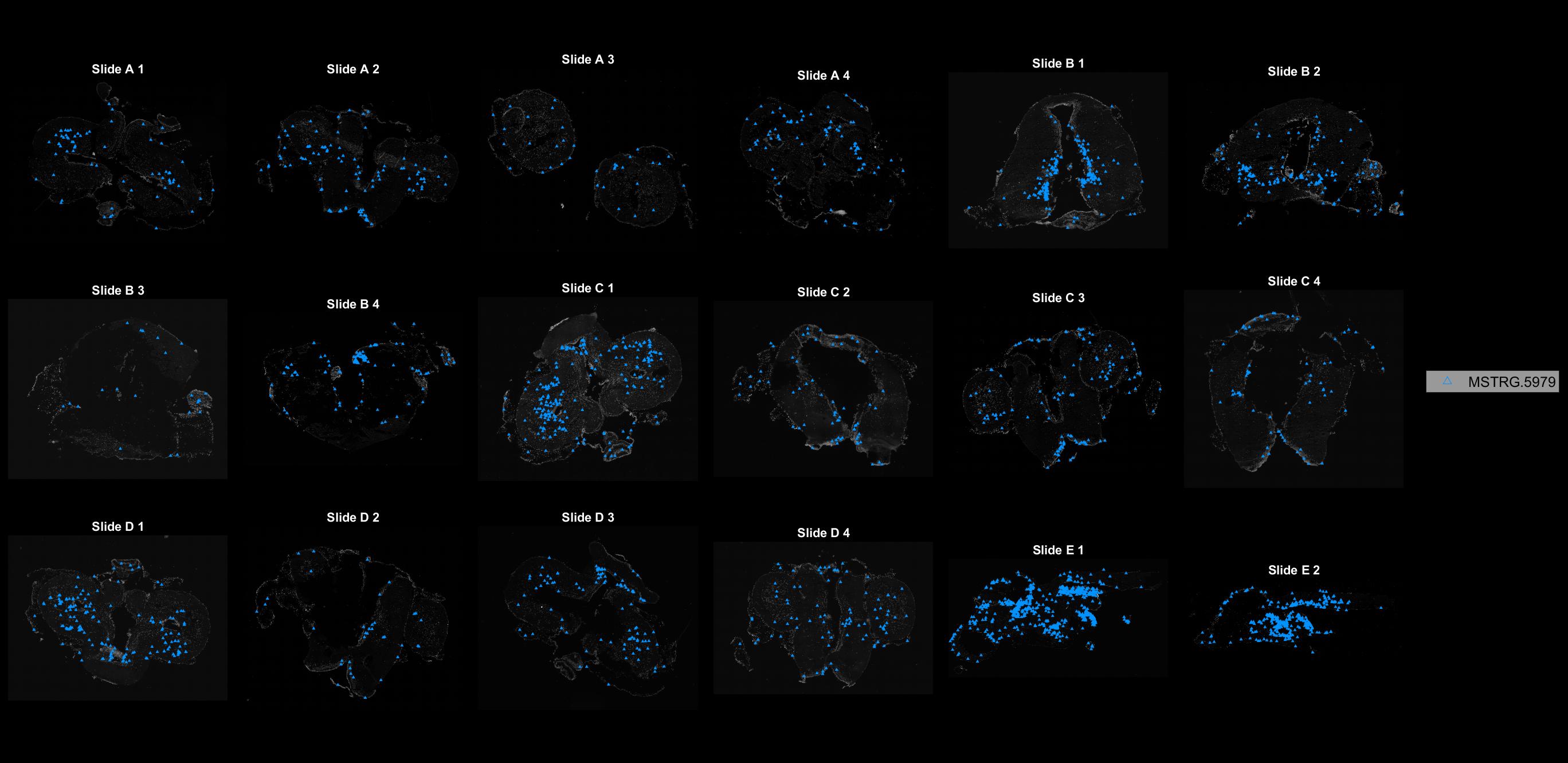

Supplement: Supplementary file 6 — In situ images produced in this study. [file 41559_2023_2170_MOESM6_ESM.zip › ISS/MSTRG.5979.jpg]

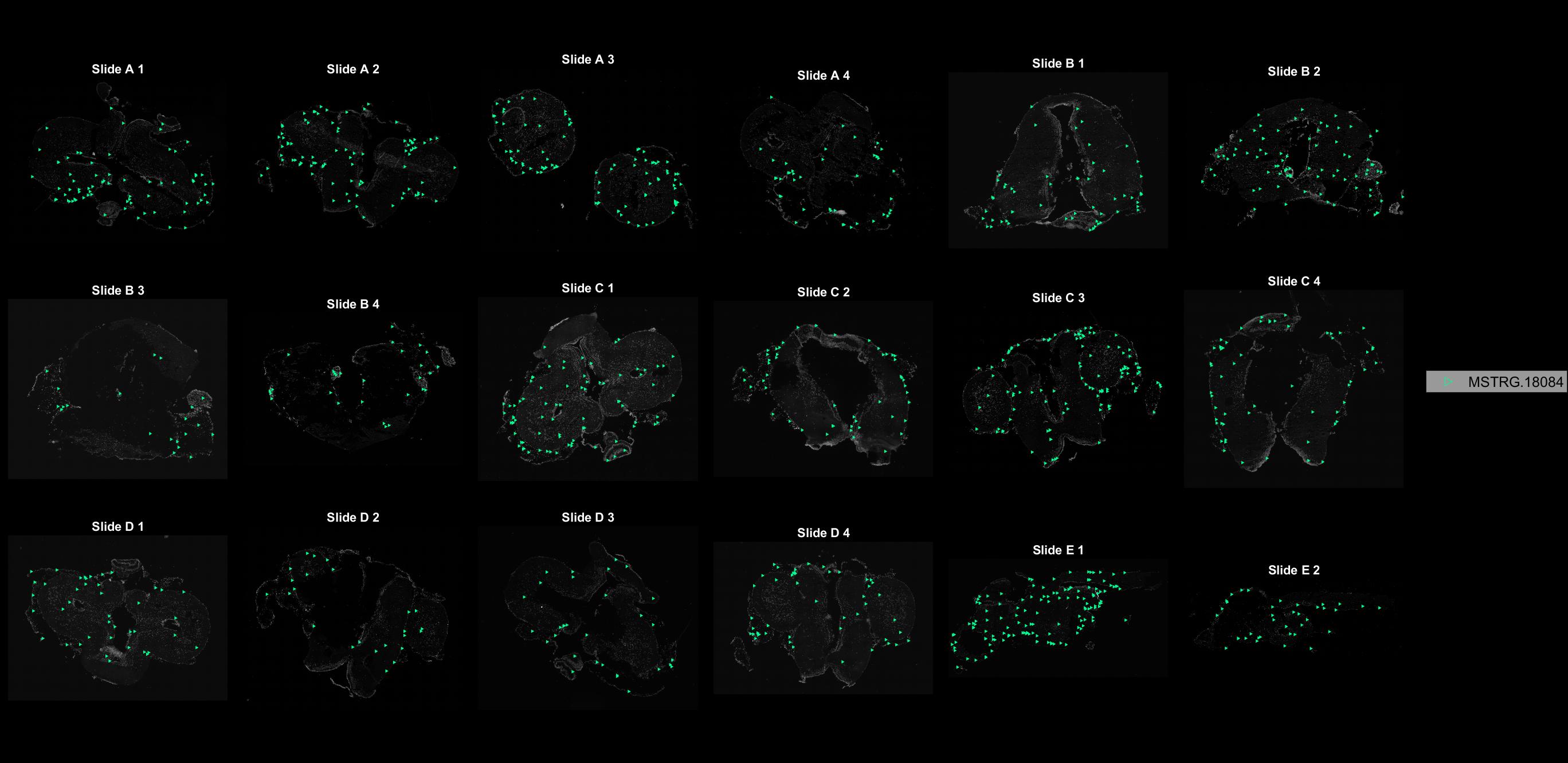

Supplement: Supplementary file 6 — In situ images produced in this study. [file 41559_2023_2170_MOESM6_ESM.zip › ISS/MSTRG.18084.jpg]

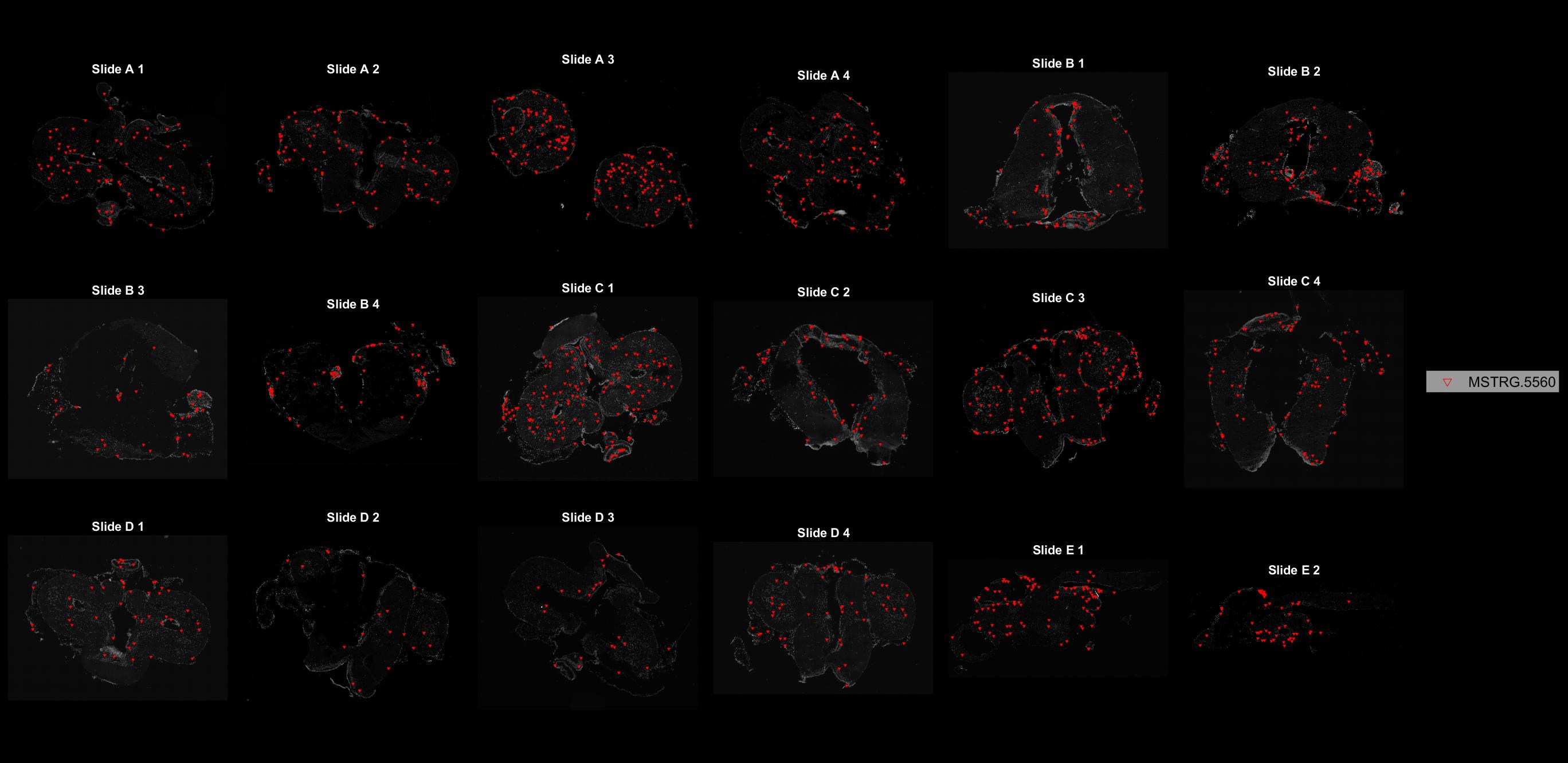

Supplement: Supplementary file 6 — In situ images produced in this study. [file 41559_2023_2170_MOESM6_ESM.zip › ISS/MSTRG.5560.jpg]

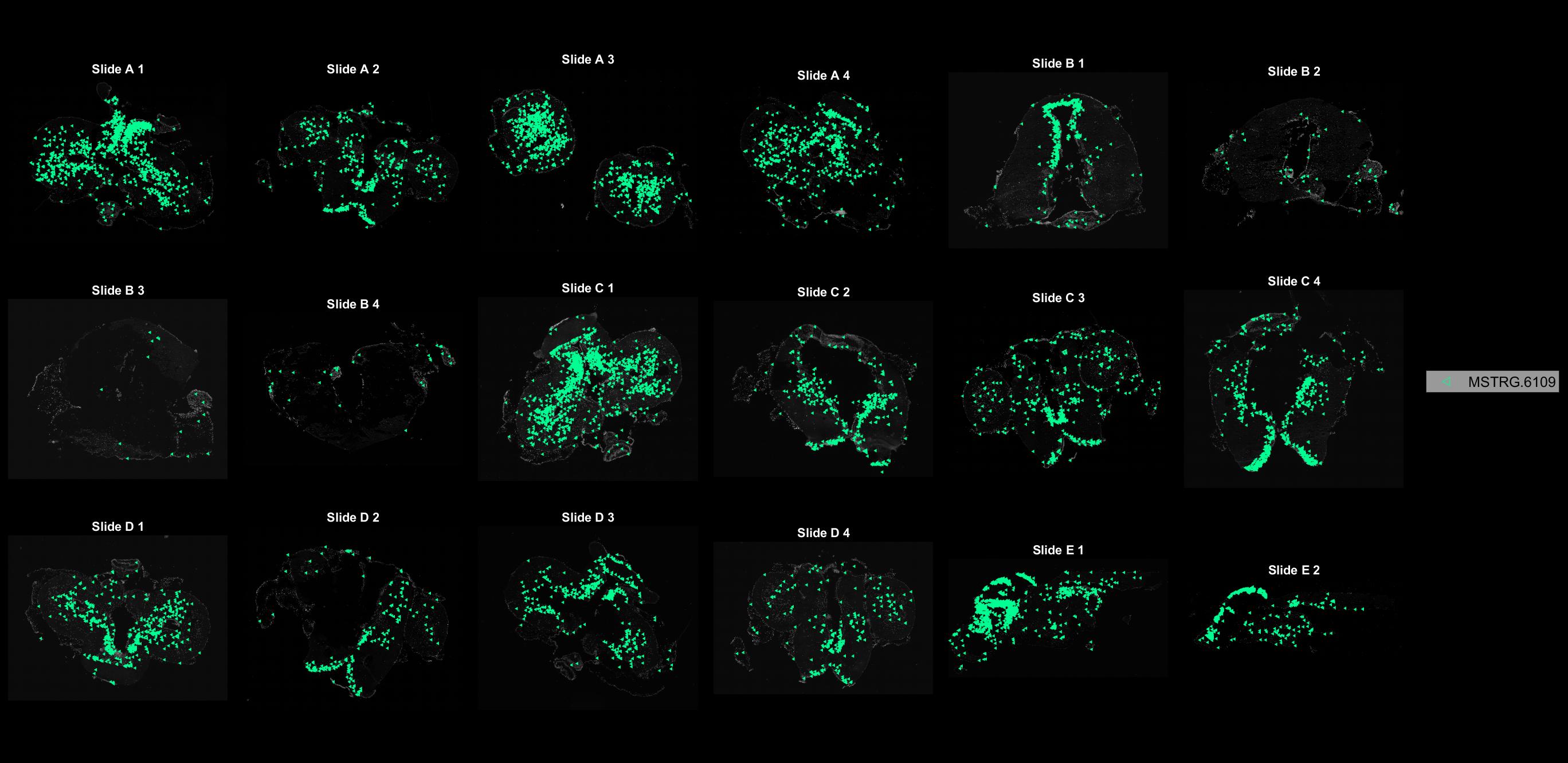

Supplement: Supplementary file 6 — In situ images produced in this study. [file 41559_2023_2170_MOESM6_ESM.zip › ISS/MSTRG.6109.jpg]

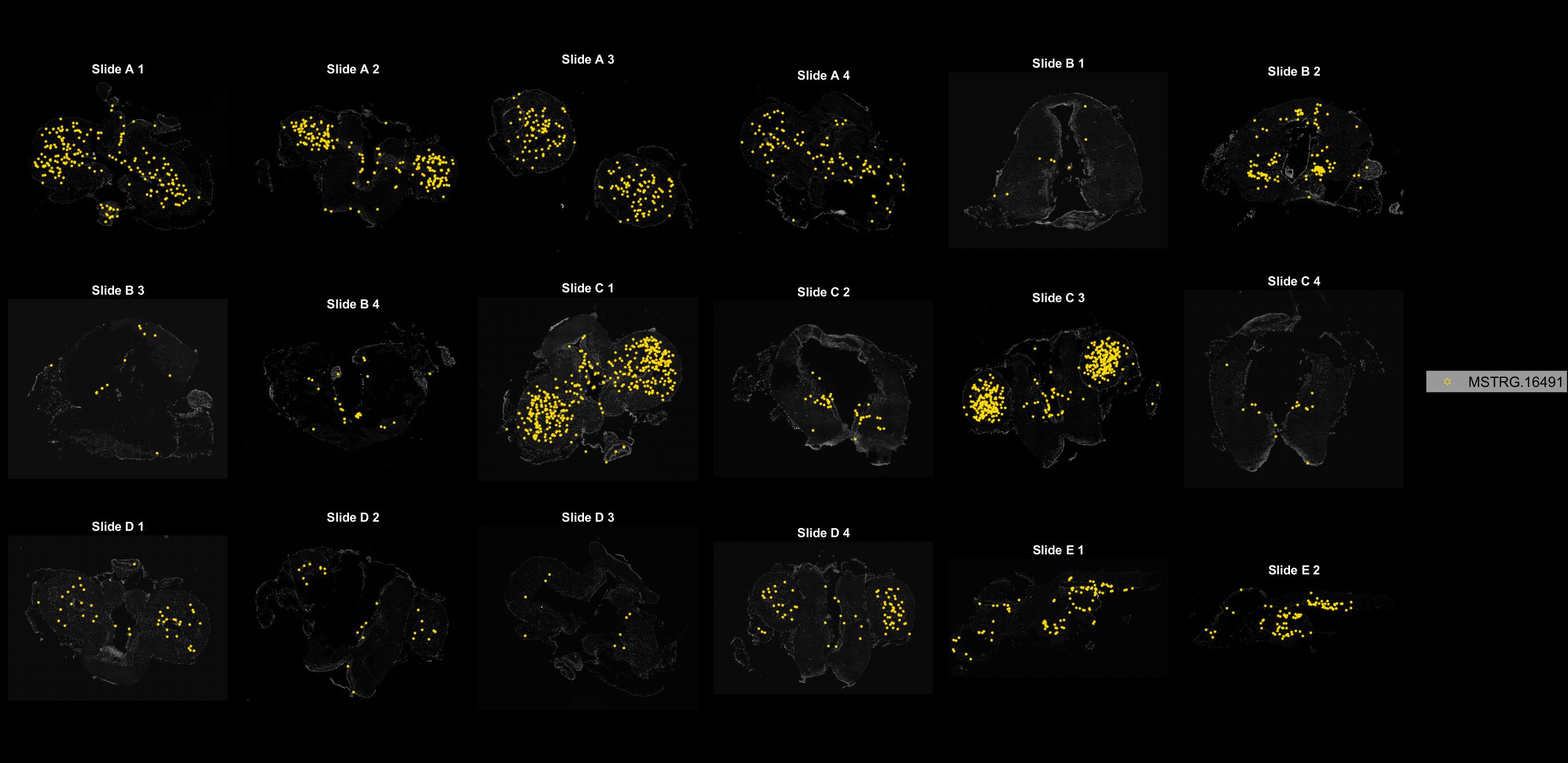

Supplement: Supplementary file 6 — In situ images produced in this study. [file 41559_2023_2170_MOESM6_ESM.zip › ISS/MSTRG.16491.jpg]

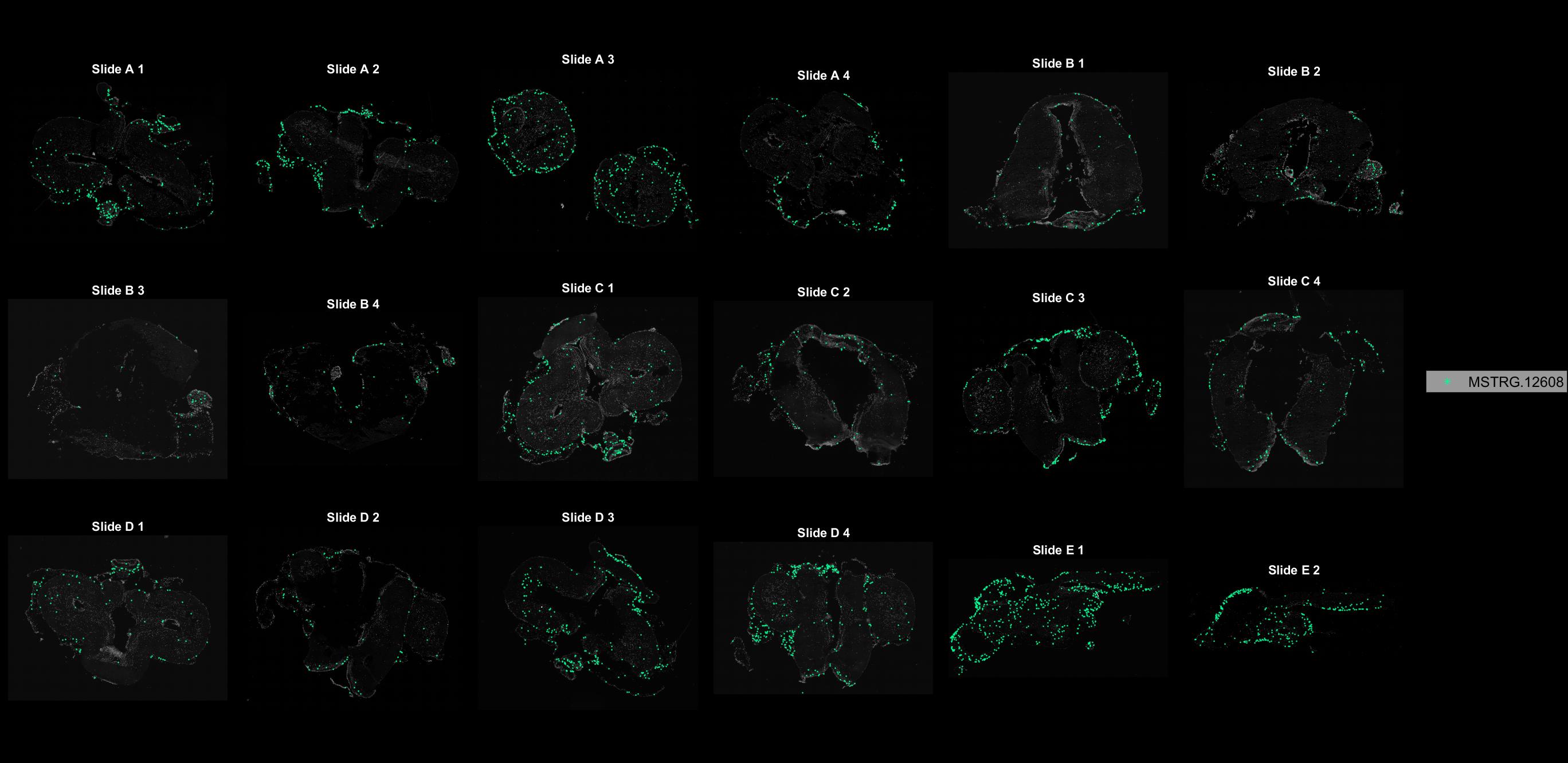

Supplement: Supplementary file 6 — In situ images produced in this study. [file 41559_2023_2170_MOESM6_ESM.zip › ISS/MSTRG.12608.jpg]

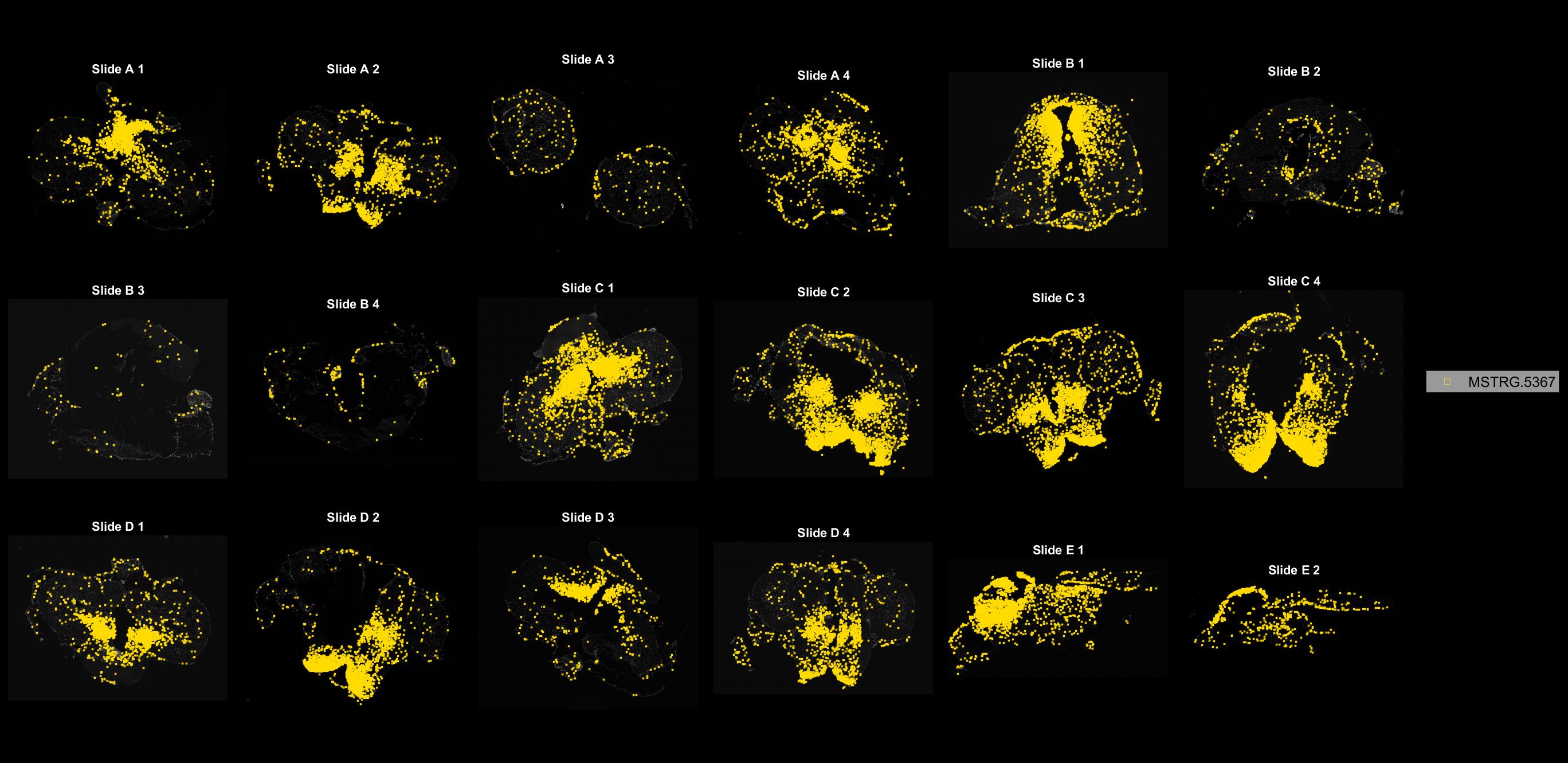

Supplement: Supplementary file 6 — In situ images produced in this study. [file 41559_2023_2170_MOESM6_ESM.zip › ISS/MSTRG.5367.jpg]

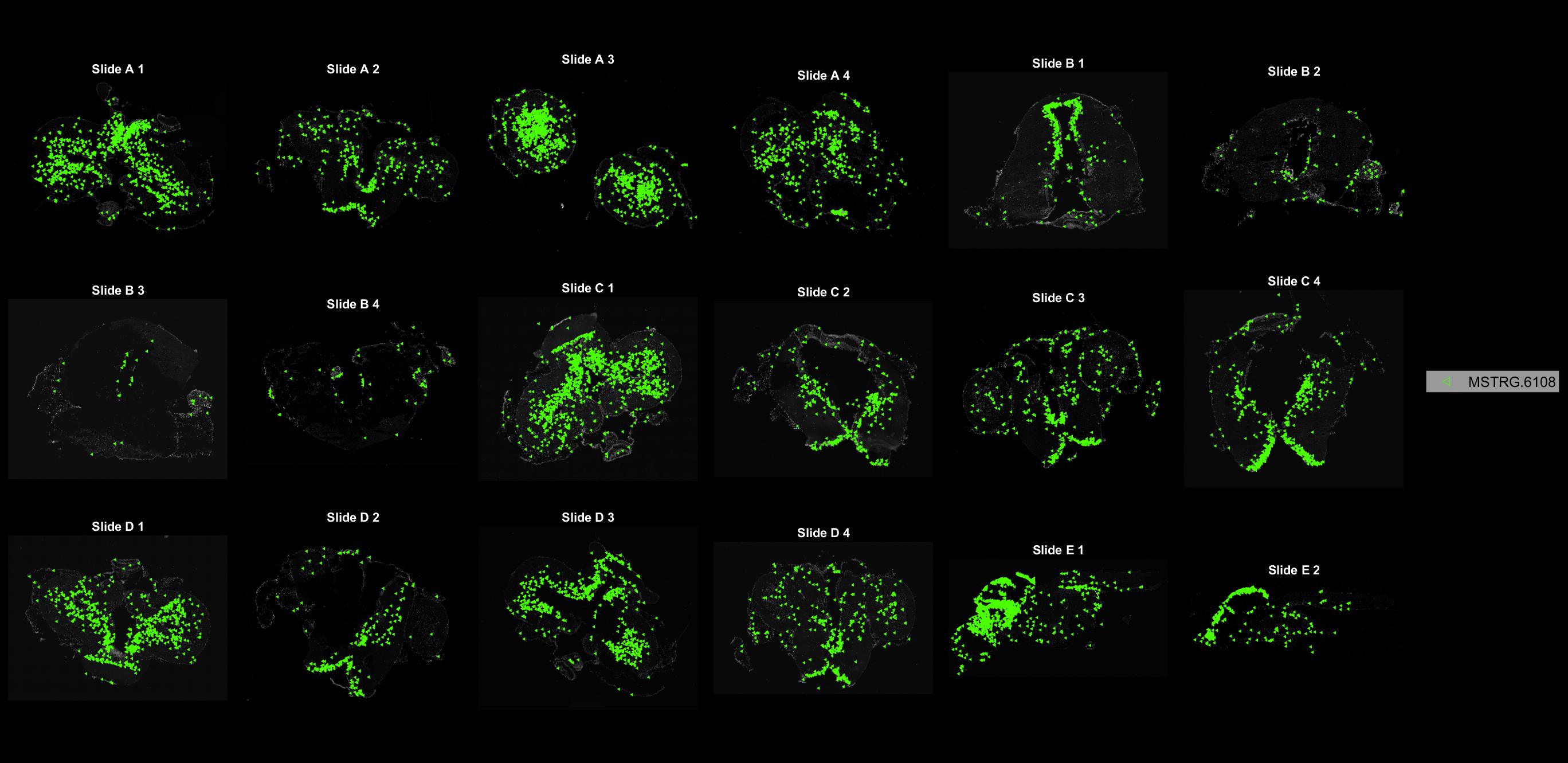

Supplement: Supplementary file 6 — In situ images produced in this study. [file 41559_2023_2170_MOESM6_ESM.zip › ISS/MSTRG.6108.jpg]

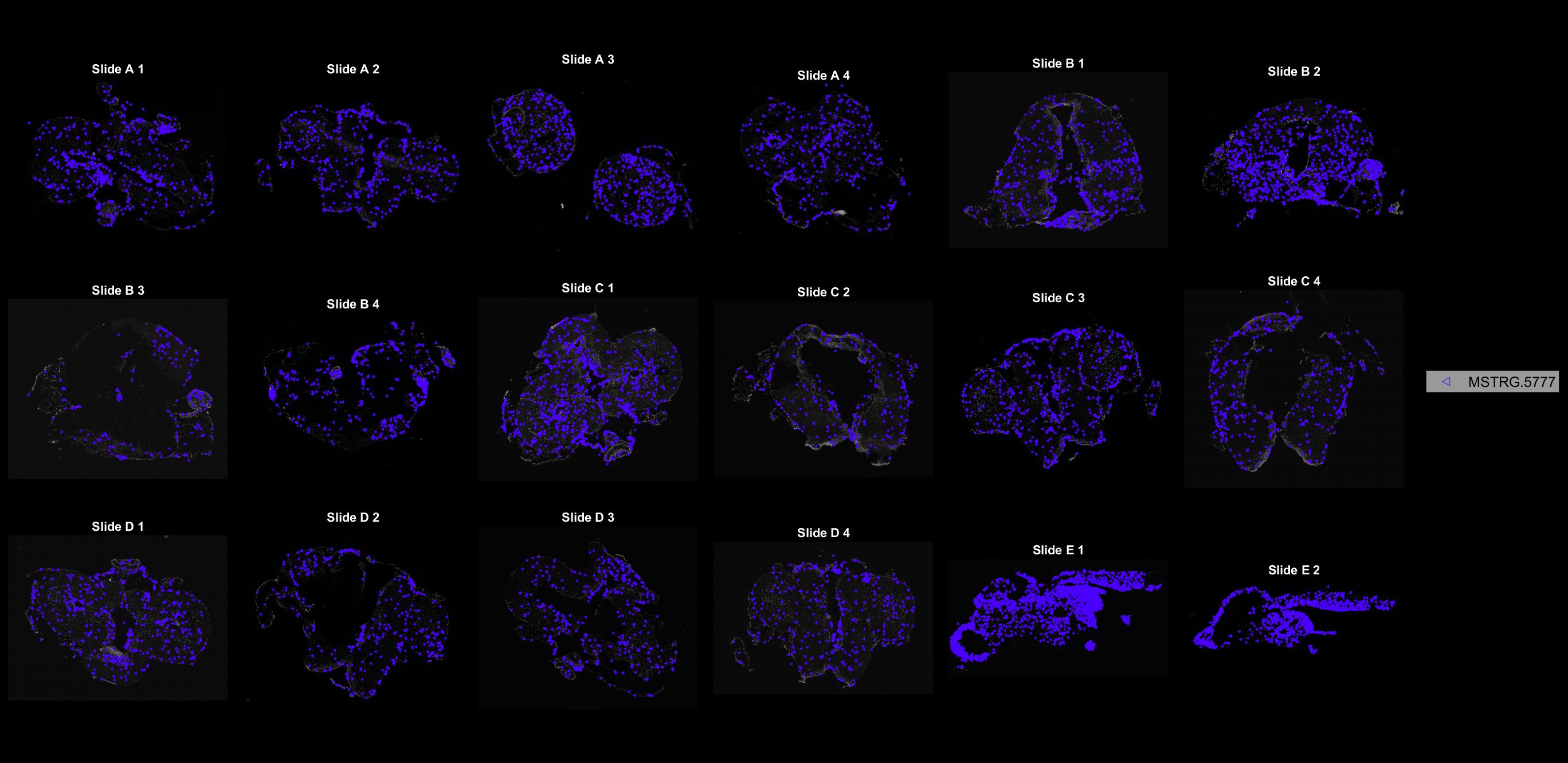

Supplement: Supplementary file 6 — In situ images produced in this study. [file 41559_2023_2170_MOESM6_ESM.zip › ISS/MSTRG.5777.jpg]

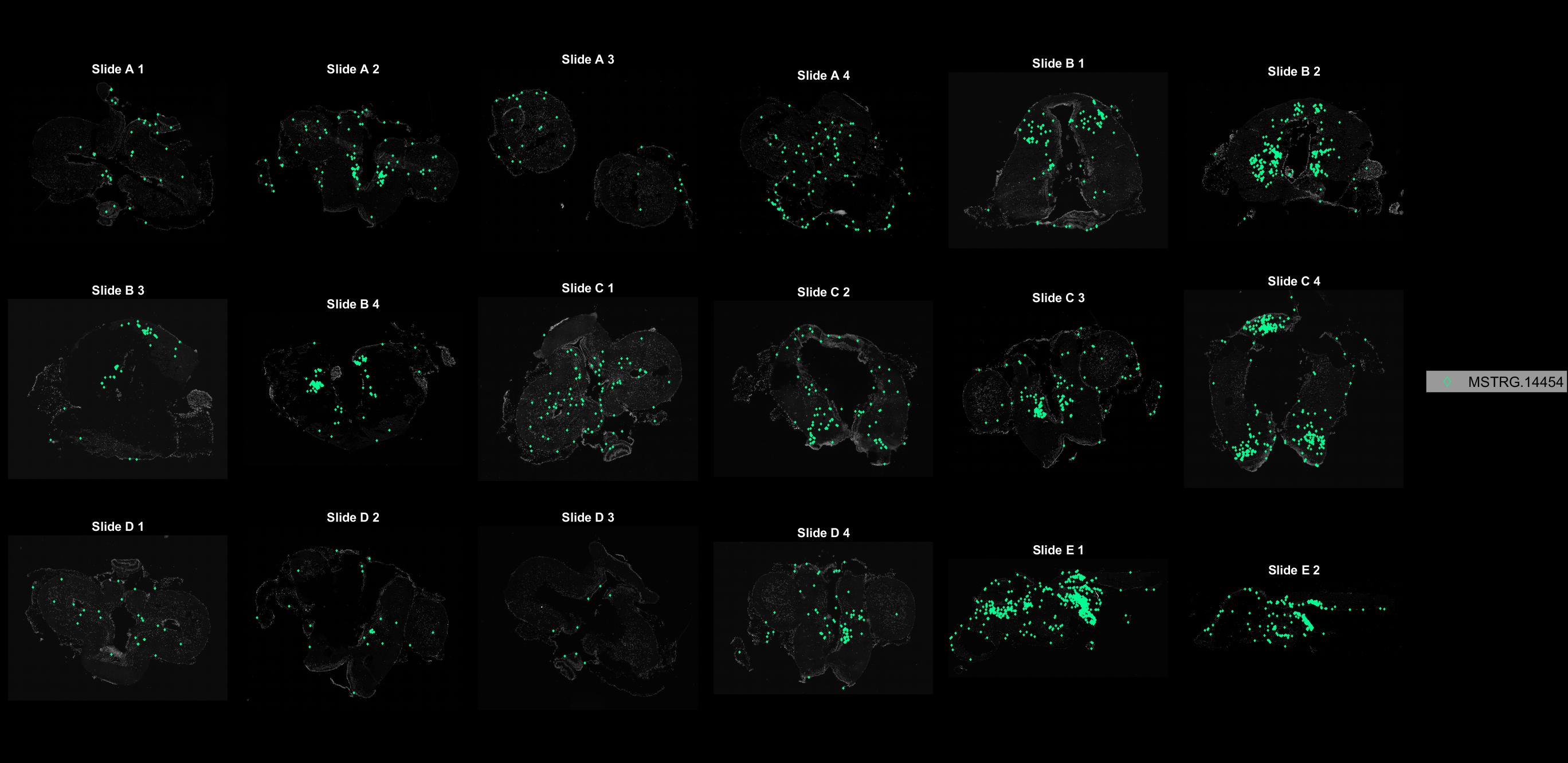

Supplement: Supplementary file 6 — In situ images produced in this study. [file 41559_2023_2170_MOESM6_ESM.zip › ISS/MSTRG.14454.jpg]

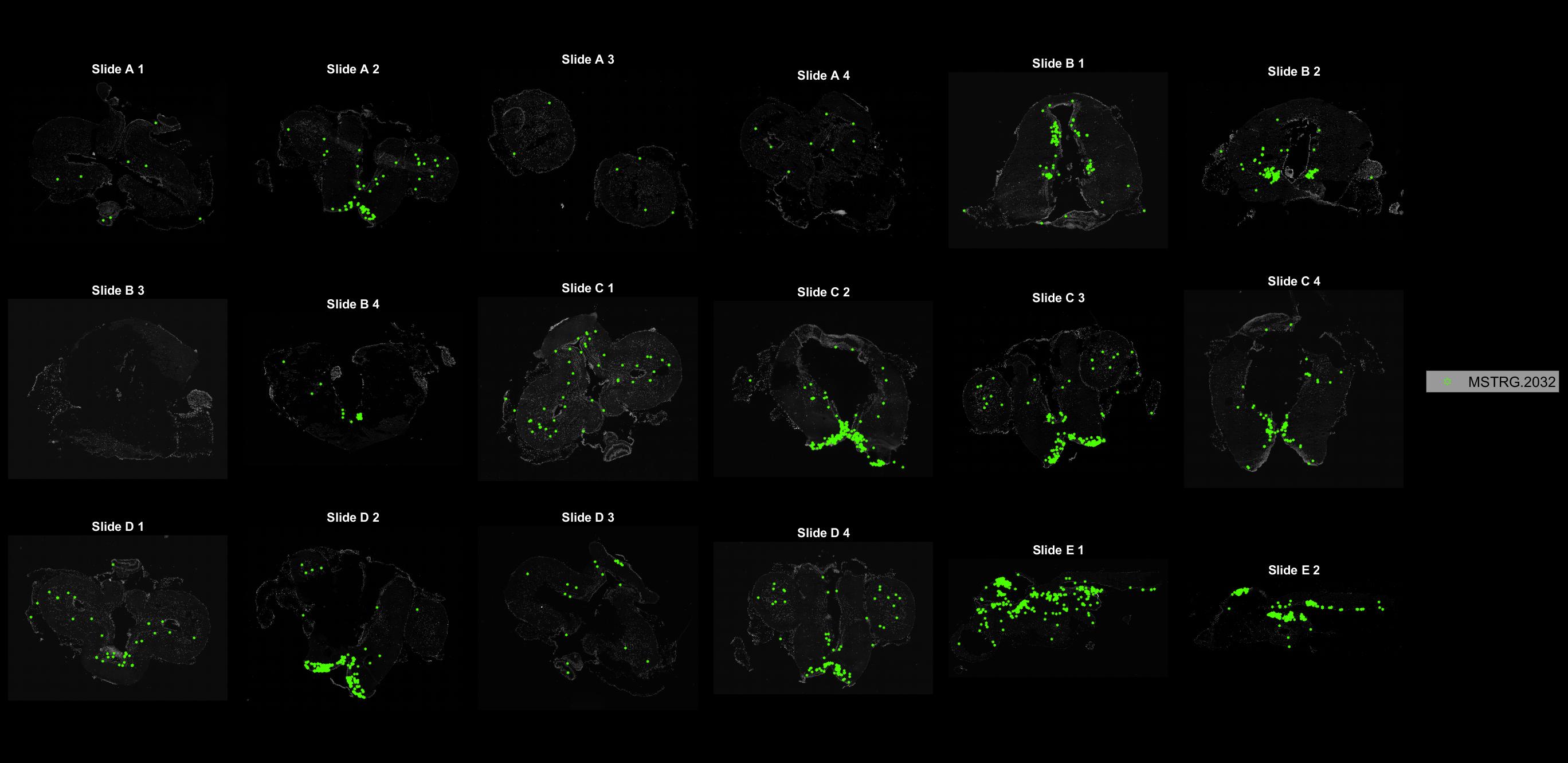

Supplement: Supplementary file 6 — In situ images produced in this study. [file 41559_2023_2170_MOESM6_ESM.zip › ISS/MSTRG.2032.jpg]

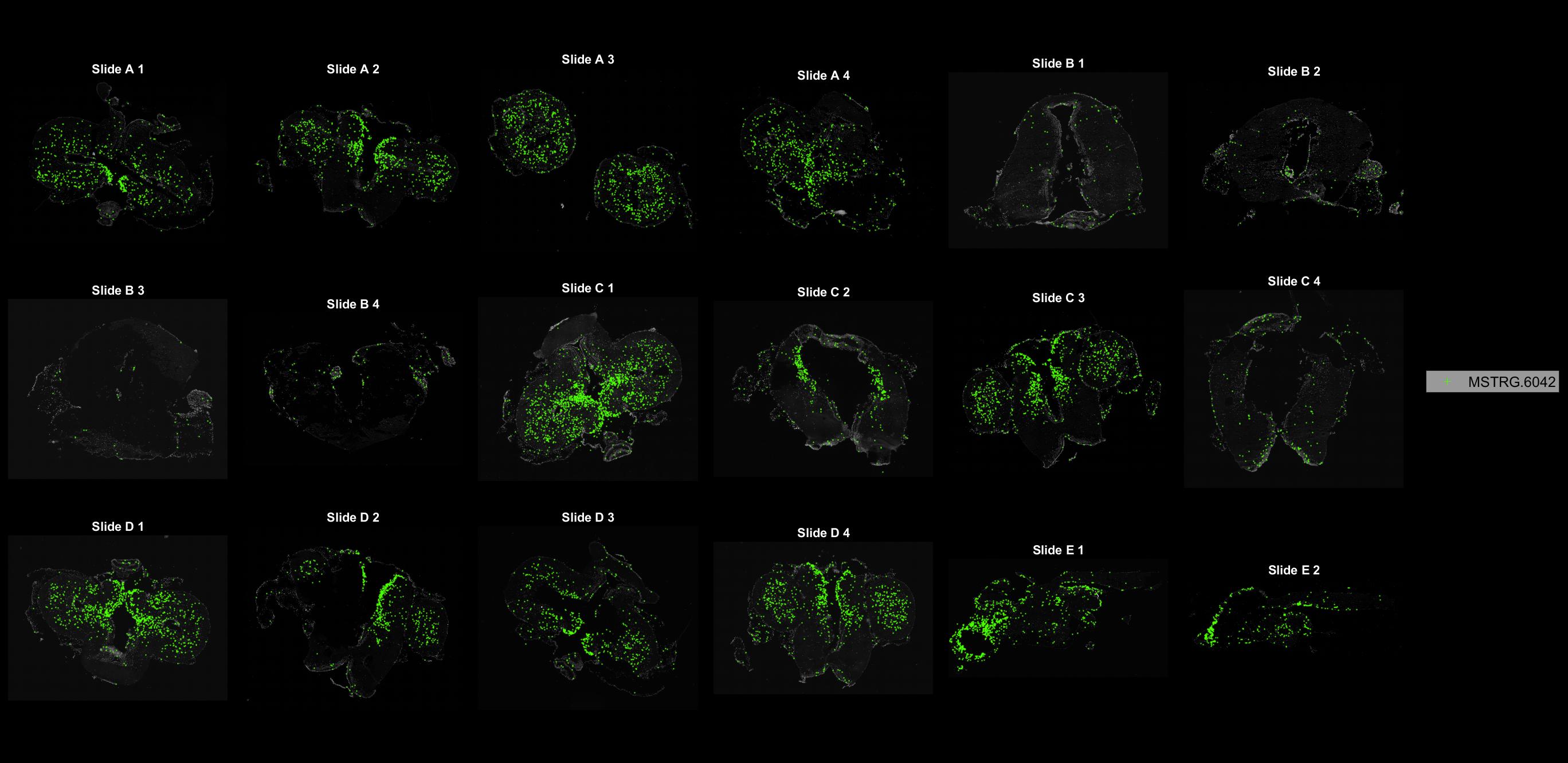

Supplement: Supplementary file 6 — In situ images produced in this study. [file 41559_2023_2170_MOESM6_ESM.zip › ISS/MSTRG.6042.jpg]

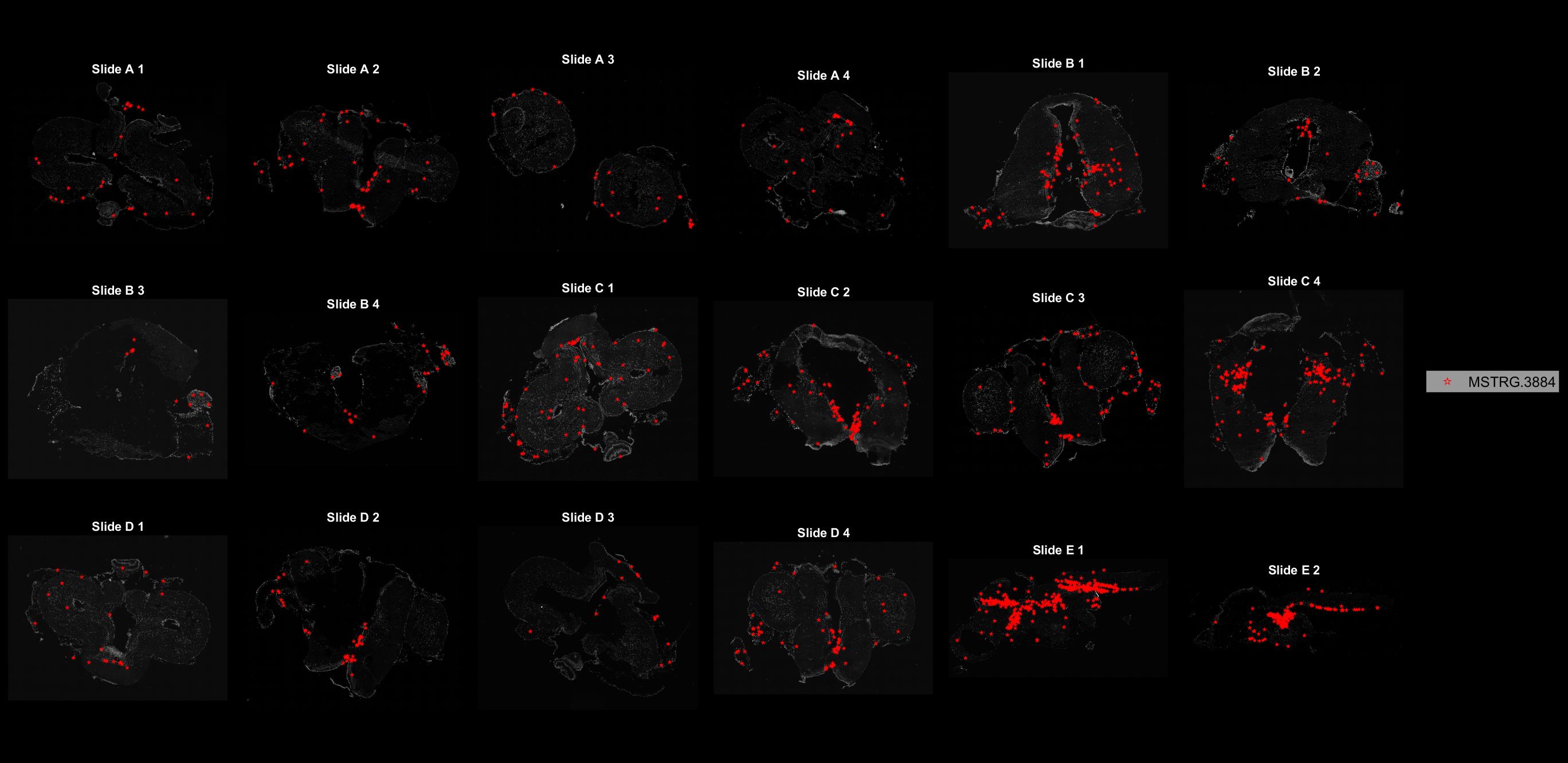

Supplement: Supplementary file 6 — In situ images produced in this study. [file 41559_2023_2170_MOESM6_ESM.zip › ISS/MSTRG.3884.jpg]

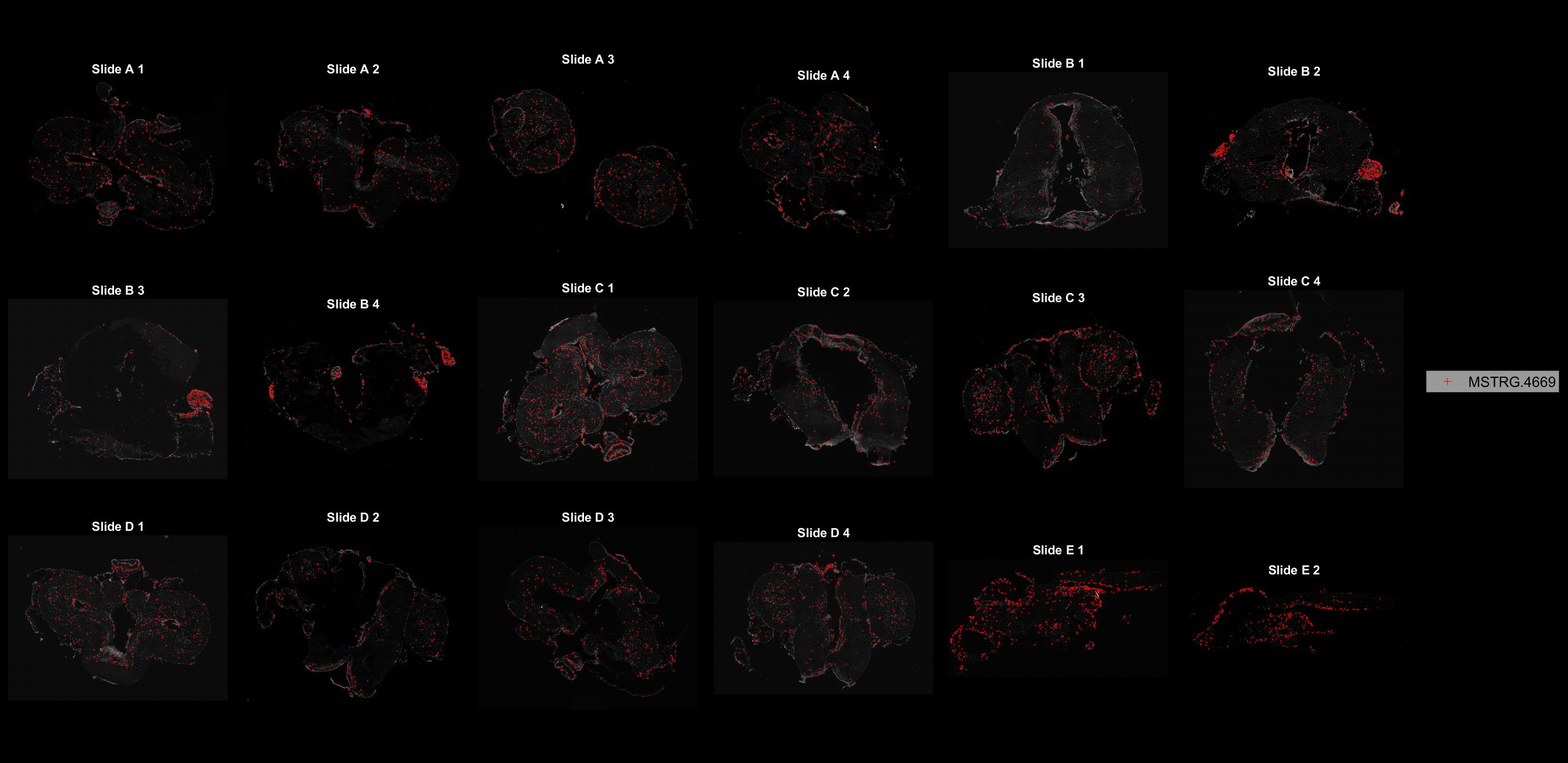

Supplement: Supplementary file 6 — In situ images produced in this study. [file 41559_2023_2170_MOESM6_ESM.zip › ISS/MSTRG.4669.jpg]

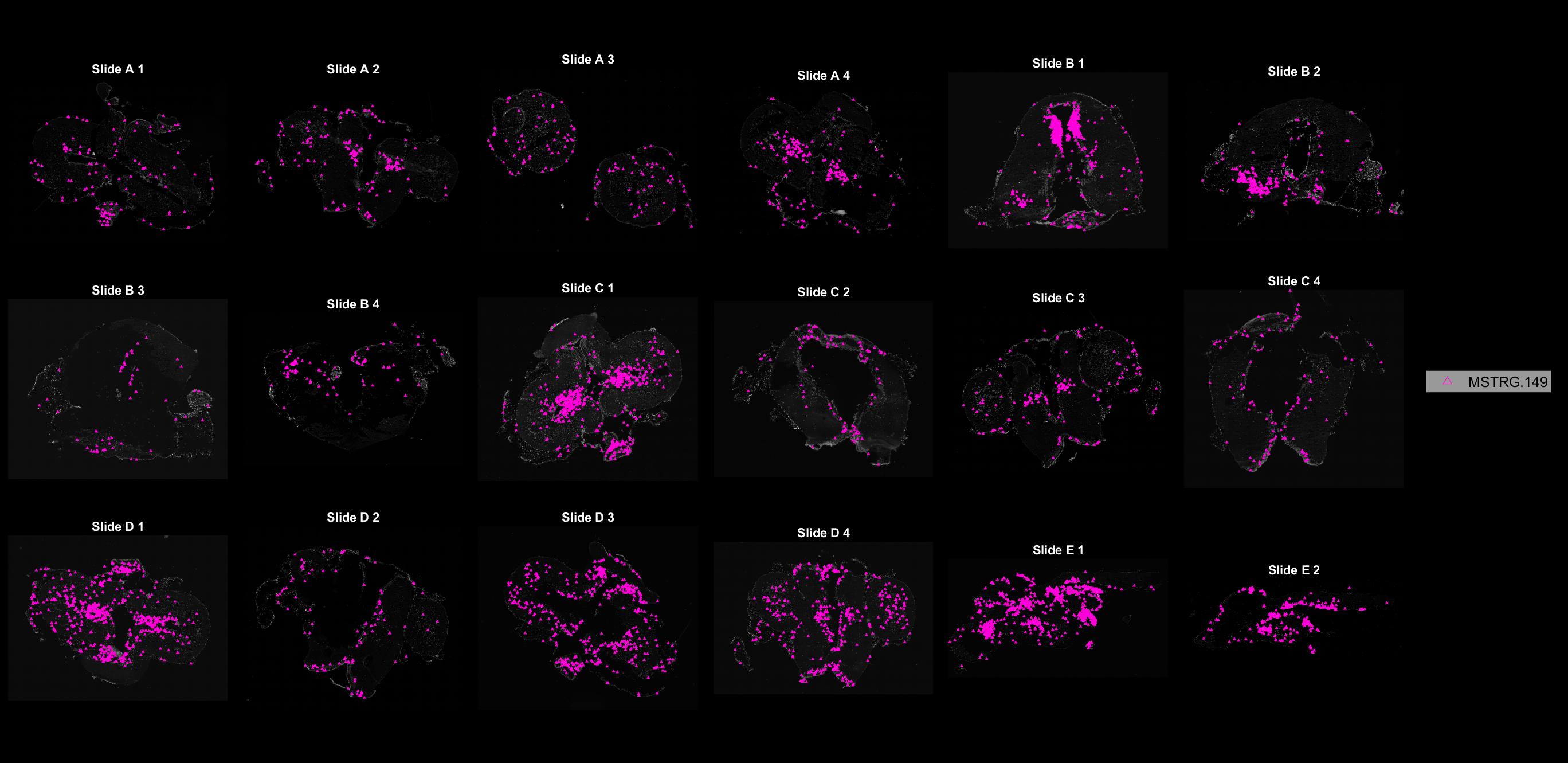

Supplement: Supplementary file 6 — In situ images produced in this study. [file 41559_2023_2170_MOESM6_ESM.zip › ISS/MSTRG.149.jpg]

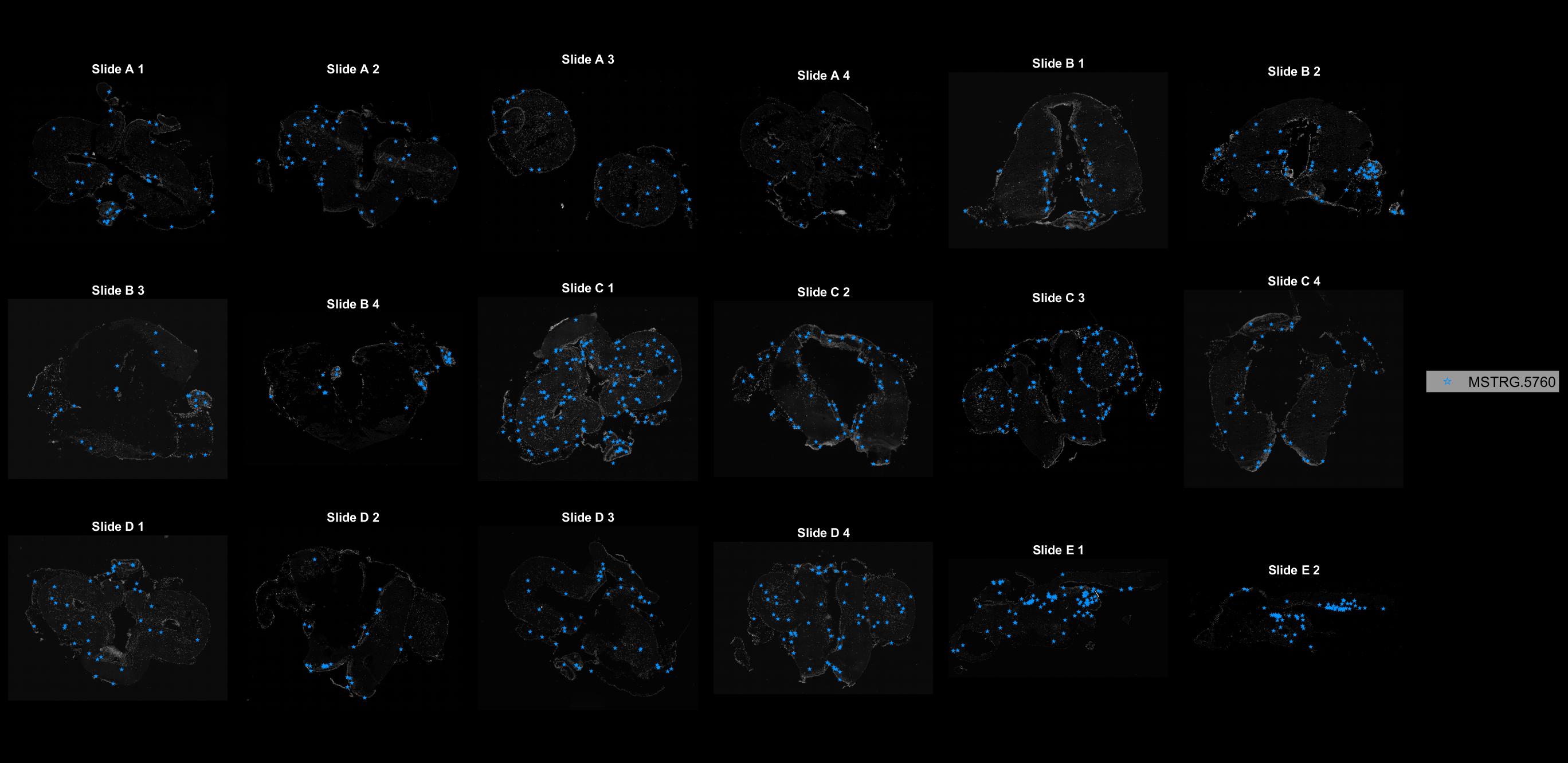

Supplement: Supplementary file 6 — In situ images produced in this study. [file 41559_2023_2170_MOESM6_ESM.zip › ISS/MSTRG.5760.jpg]

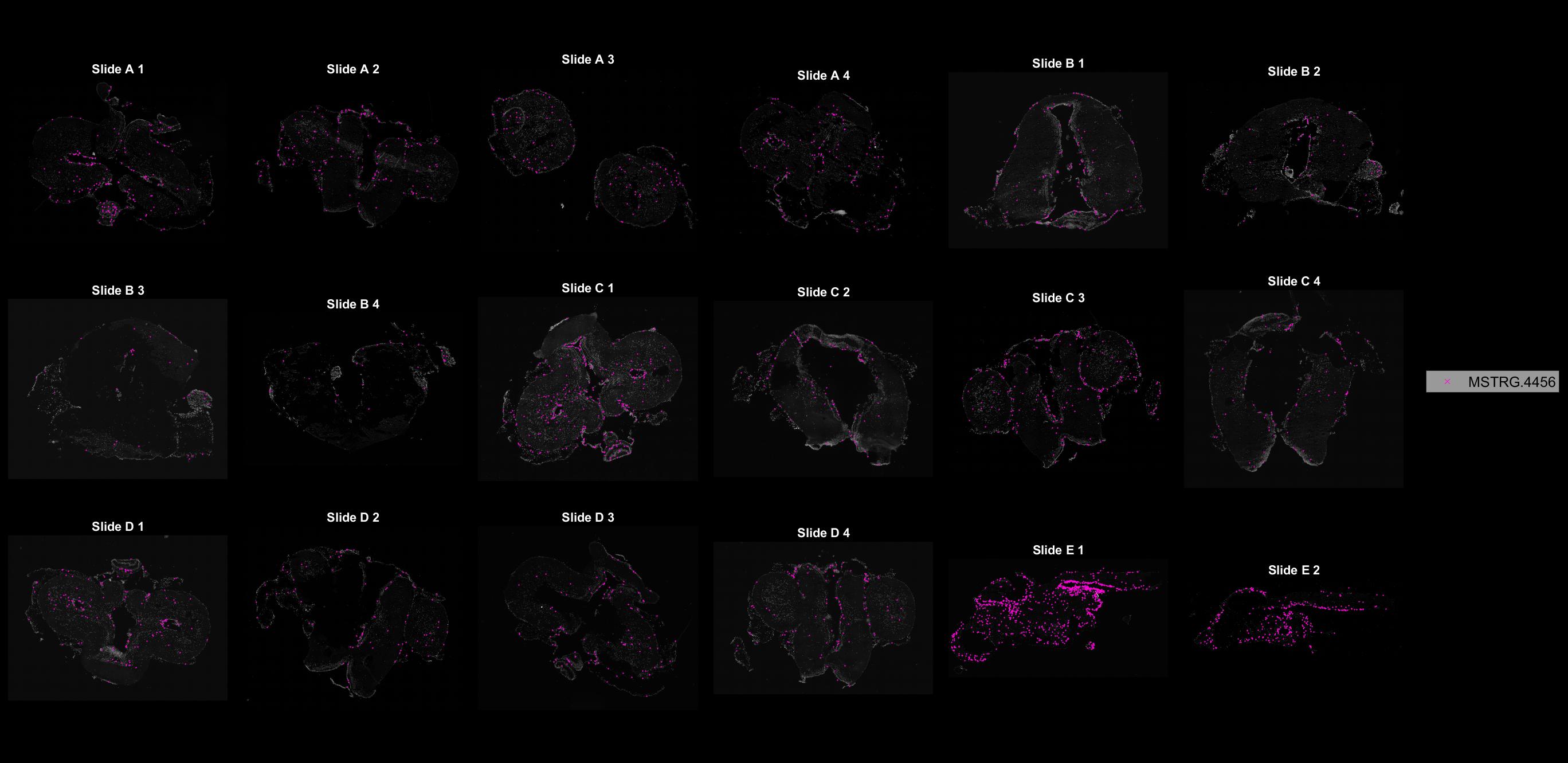

Supplement: Supplementary file 6 — In situ images produced in this study. [file 41559_2023_2170_MOESM6_ESM.zip › ISS/MSTRG.4456.jpg]
